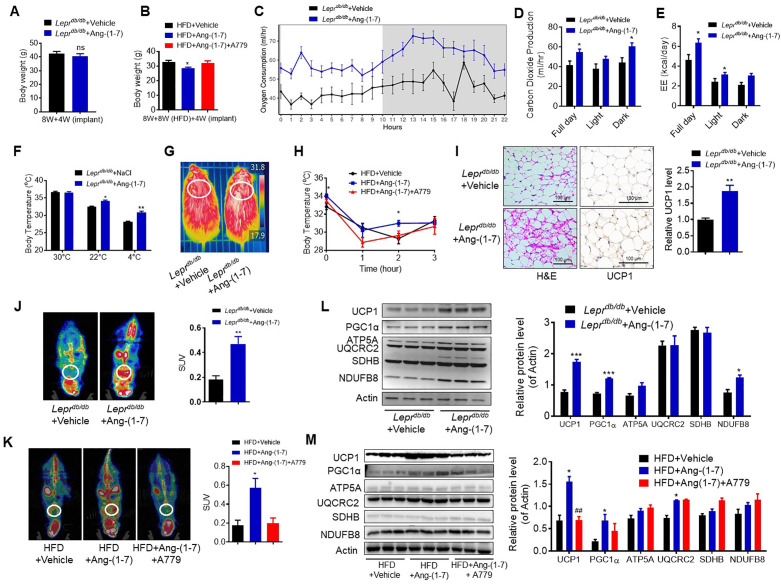# Correction: ACE2 pathway regulates thermogenesis and energy metabolism

**DOI:** 10.7554/eLife.78698

**Published:** 2022-03-22

**Authors:** Xi Cao, Ting-Ting Shi, Chuan-Hai Zhang, Wan-Zhu Jin, Li-Ni Song, Yi-Chen Zhang, Jing-Yi Liu, Fang-Yuan Yang, Charles N Rotimi, Aimin Xu, Jin-Kui Yang

**Keywords:** Mouse

Cao X, Shi T-T, Zhang C-H, Jin W-Z, Song L-N, Zhang Y-C, Liu J-Y, Yang F-Y, Rotimi CN, Xu A, Yang J-K. 2022. ACE2 pathway regulates thermogenesis and energy metabolism. *eLife* **11**:e72266. doi: 10.7554/eLife.72266.

Published 11 January 2022

After publication, it came to our attention that Figure 4L and Figure 2K had the same original gels/blots in the Source data 2 - (PowerPoint of gels or blots). After careful inspection of the original data, we found that we had mistakenly pasted and edited Figure 2K as Figure 4L. The error may be stemmed from incorrect copy-paste of the original data in the editing process, and unfortunately wrongly organized the original bands in Figure 4L. We have now updated Figure 4L with the correct data as described below. We have also updated the associated Excel sheets - (Figure 4-source data 1), Source data 1 - (original files of gels or blots) and Source data 2 - (PowerPoint of gels or blots). No changes are required in the main text of the original paper. The correction does not in any way change our conclusions.

In addition, during preparation of the correction, a production error was discovered that meant most of the source data did not display on the typeset article. This has now been corrected.

The updated Figure 4 (panel L) is shown here:

**Figure 1:**
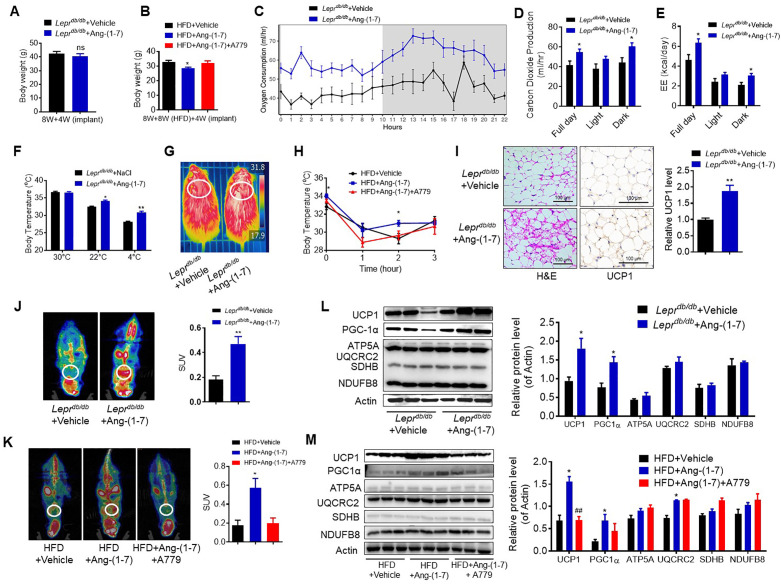


The mistakenly published Figure 4 is also shown for reference:

**Figure 2:**