# Supplementary figures and images for: ACE2 pathway regulates thermogenesis and energy metabolism
Source: eLife. 2022 Jan 11;11:e72266. doi: 10.7554/eLife.72266 (PMC8776250; doi:10.7554/eLife.72266)

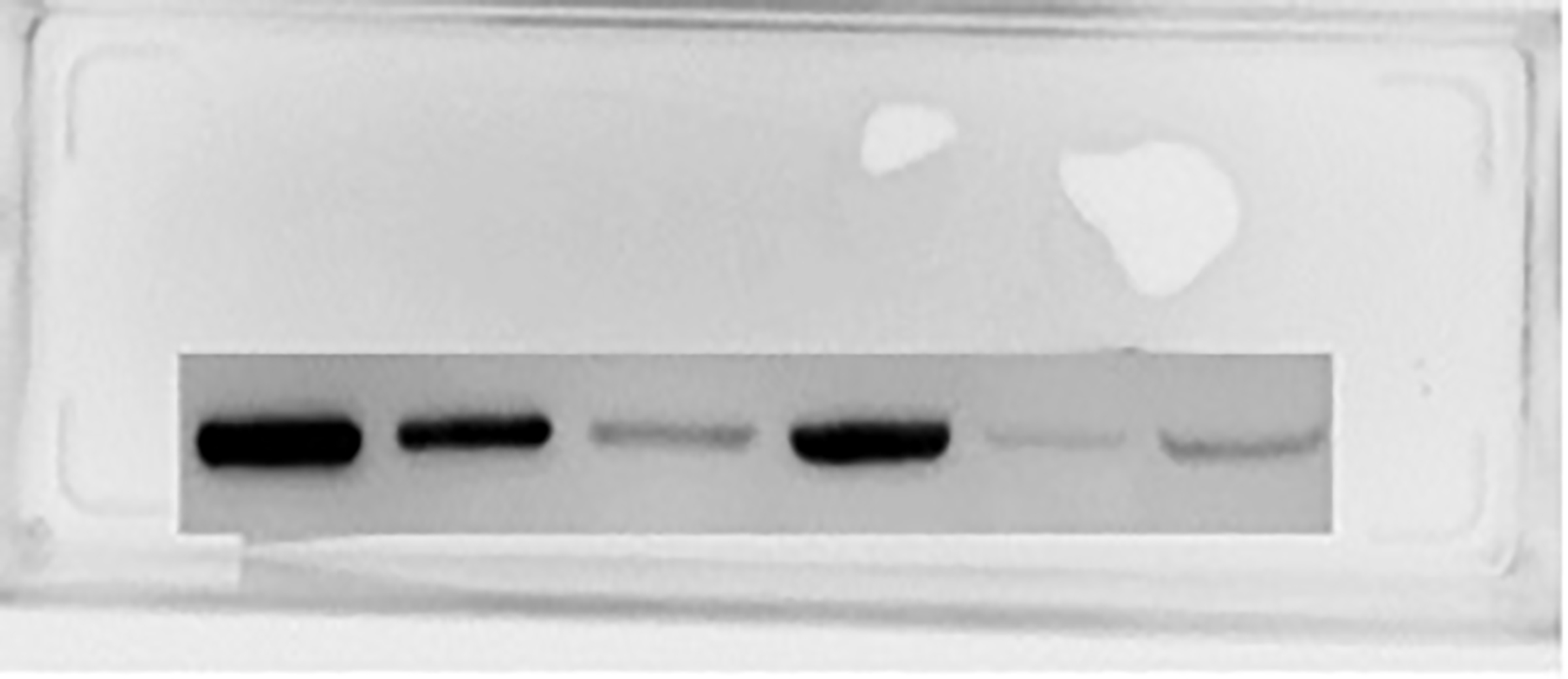

Supplement: Source data 1. [file elife-72266-data1.zip › Source data 1-original files of gels or blots/Figure 1/Figure 1A- ACE2.jpg]

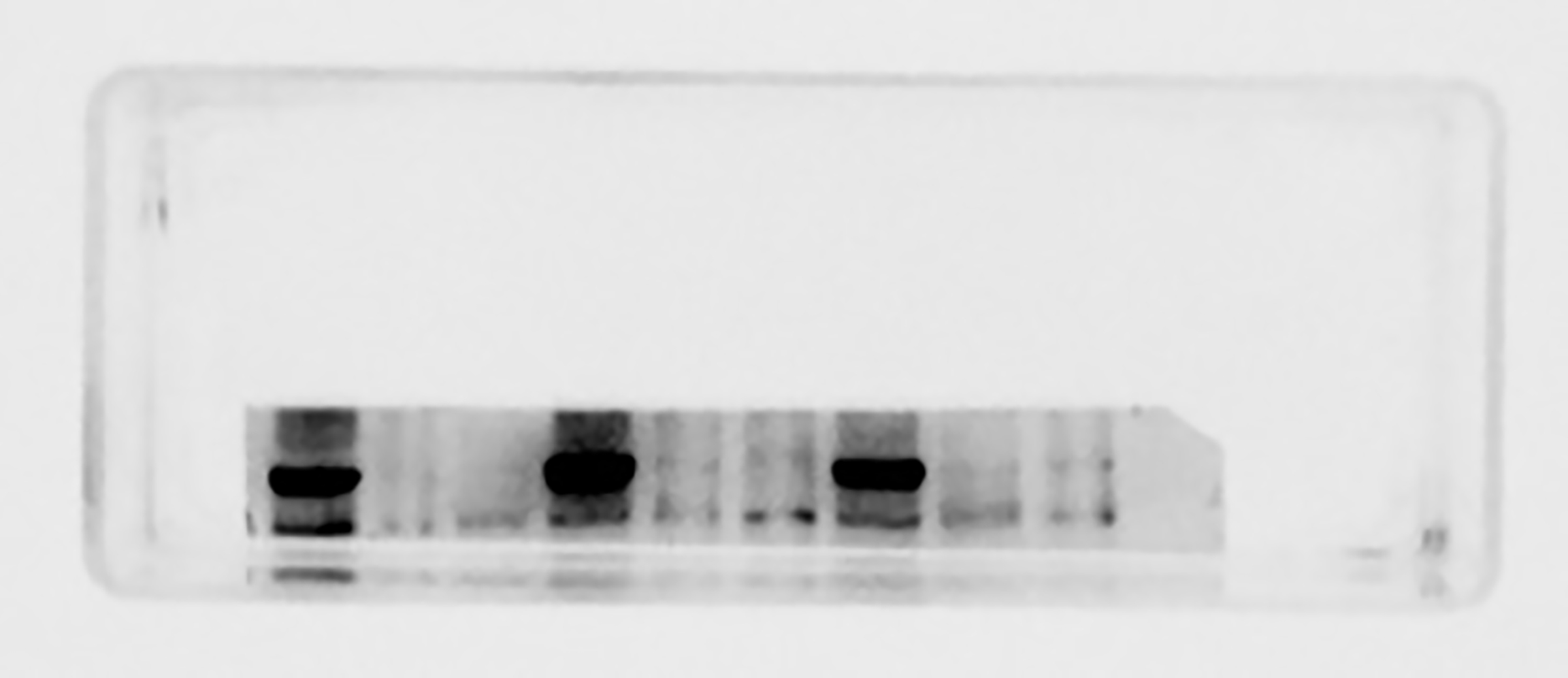

Supplement: Source data 1. [file elife-72266-data1.zip › Source data 1-original files of gels or blots/Figure 1/Figure 1A- Mas.jpg]

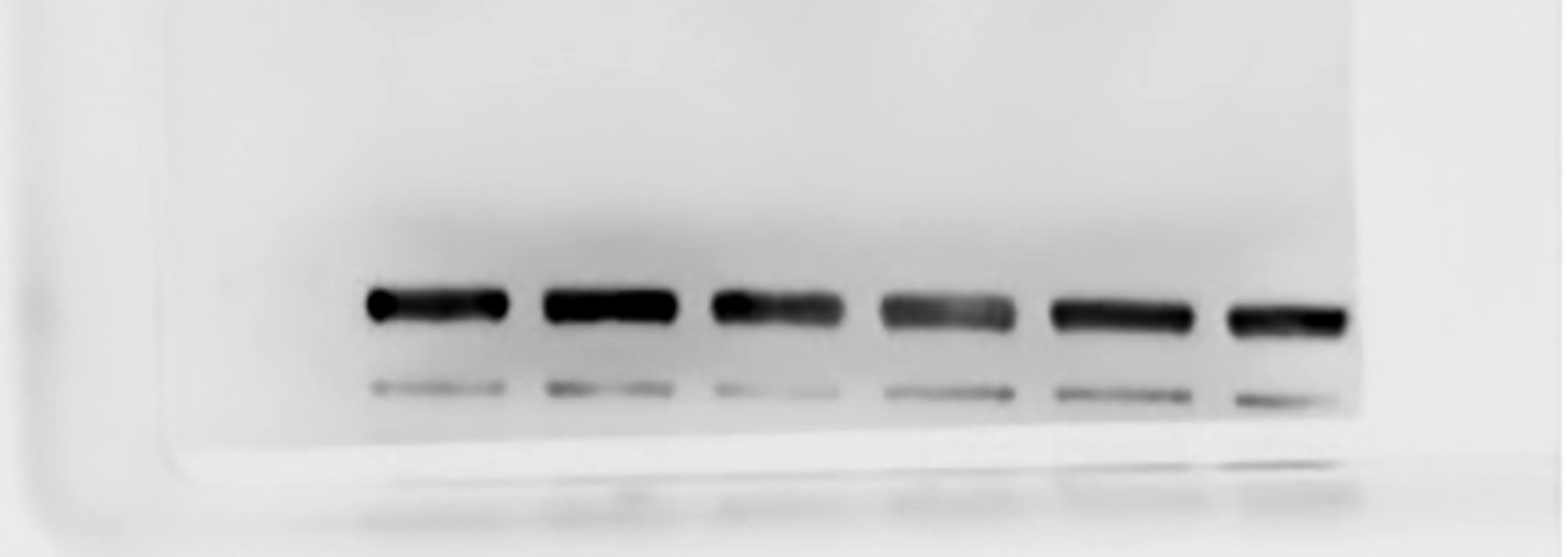

Supplement: Source data 1. [file elife-72266-data1.zip › Source data 1-original files of gels or blots/Figure 1/Figure 1A-actin.jpg]

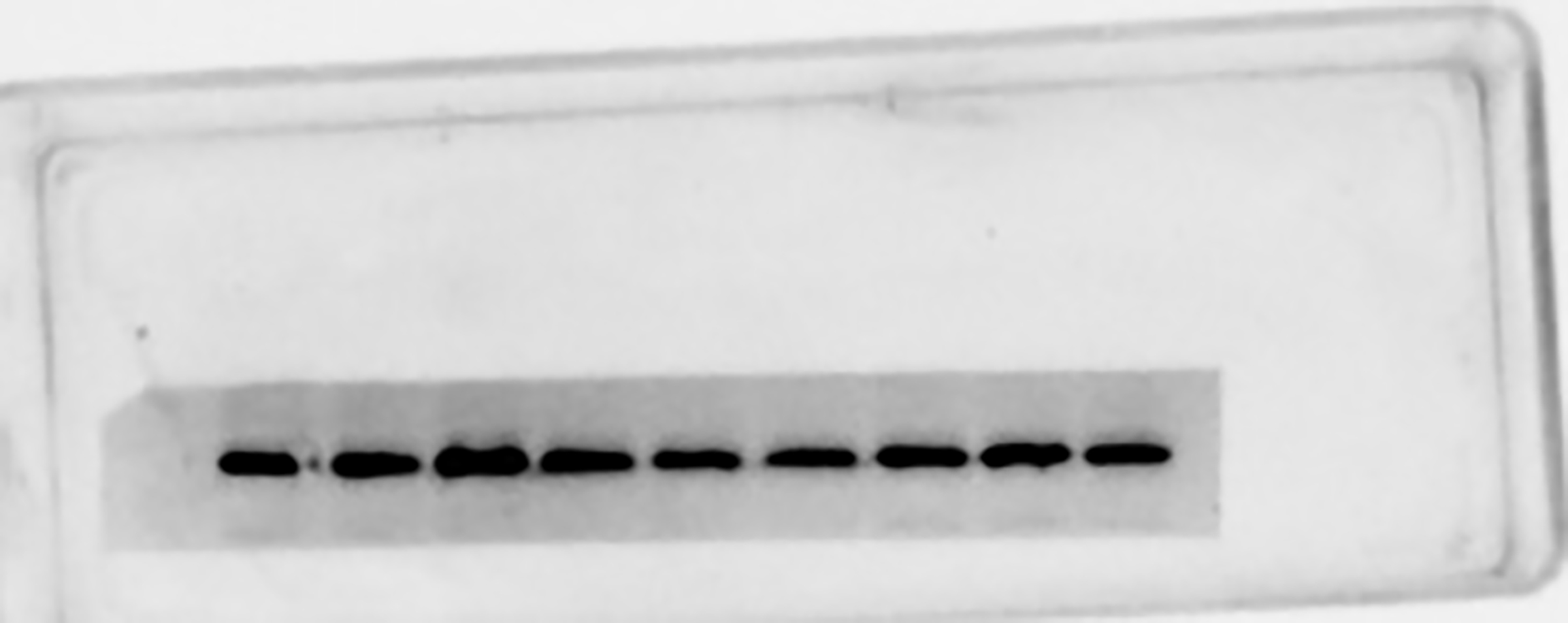

Supplement: Source data 1. [file elife-72266-data1.zip › Source data 1-original files of gels or blots/Figure 1/Figure 1A-actin2.jpg]

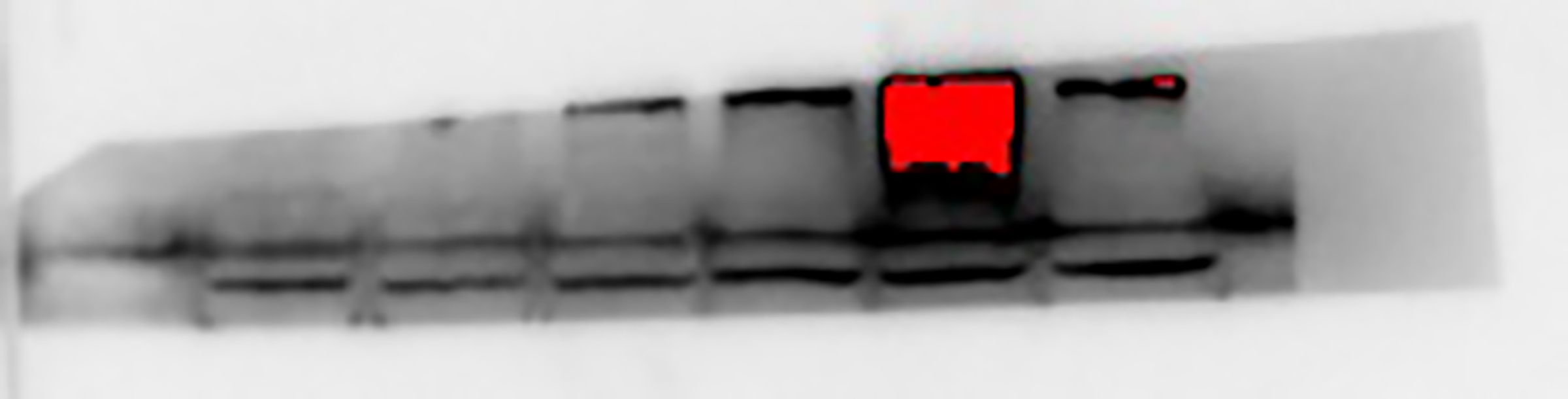

Supplement: Source data 1. [file elife-72266-data1.zip › Source data 1-original files of gels or blots/Figure 1/Figure 1B- ACE2.jpg]

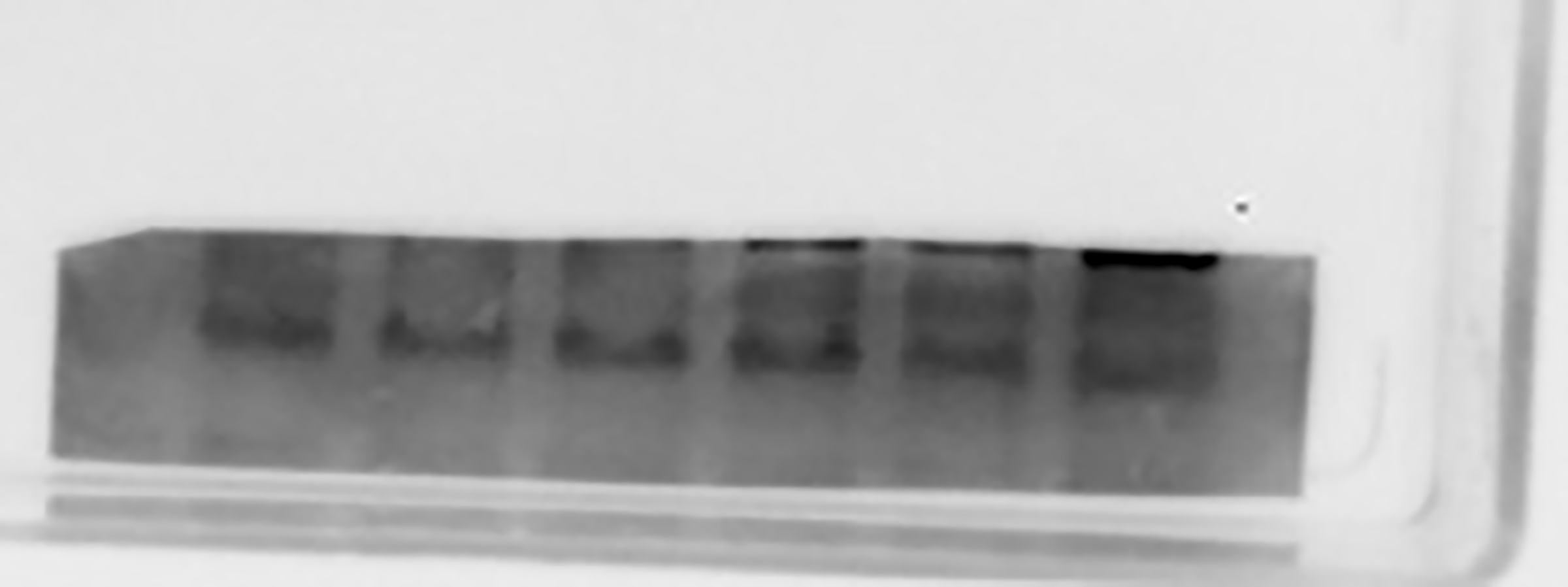

Supplement: Source data 1. [file elife-72266-data1.zip › Source data 1-original files of gels or blots/Figure 1/Figure 1B- Mas.jpg]

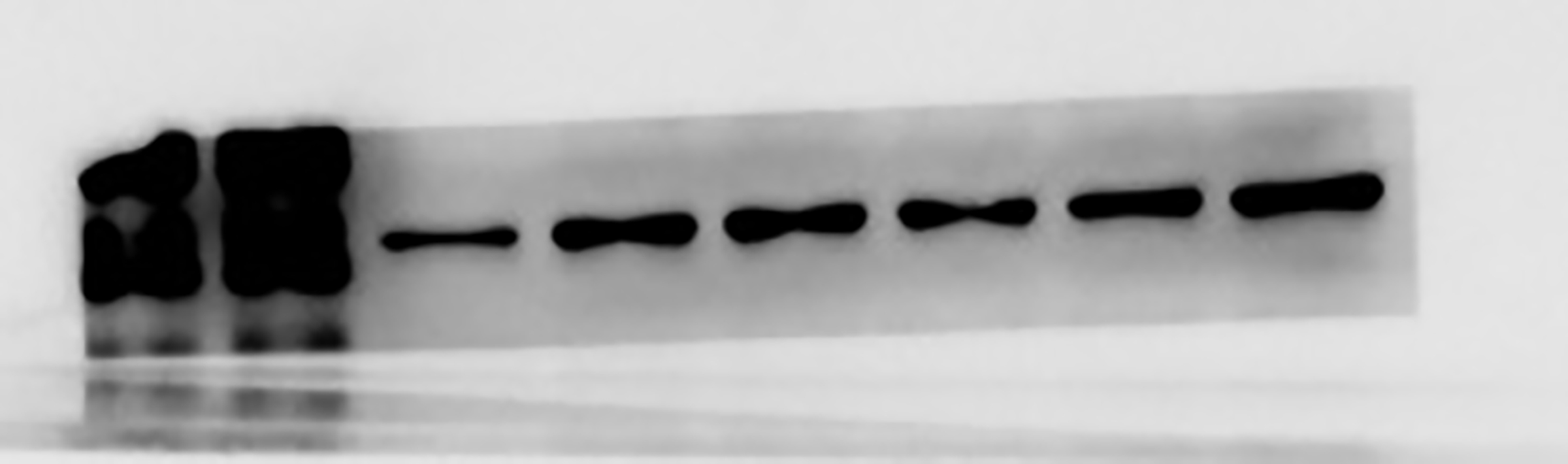

Supplement: Source data 1. [file elife-72266-data1.zip › Source data 1-original files of gels or blots/Figure 1/Figure 1B-actin.jpg]

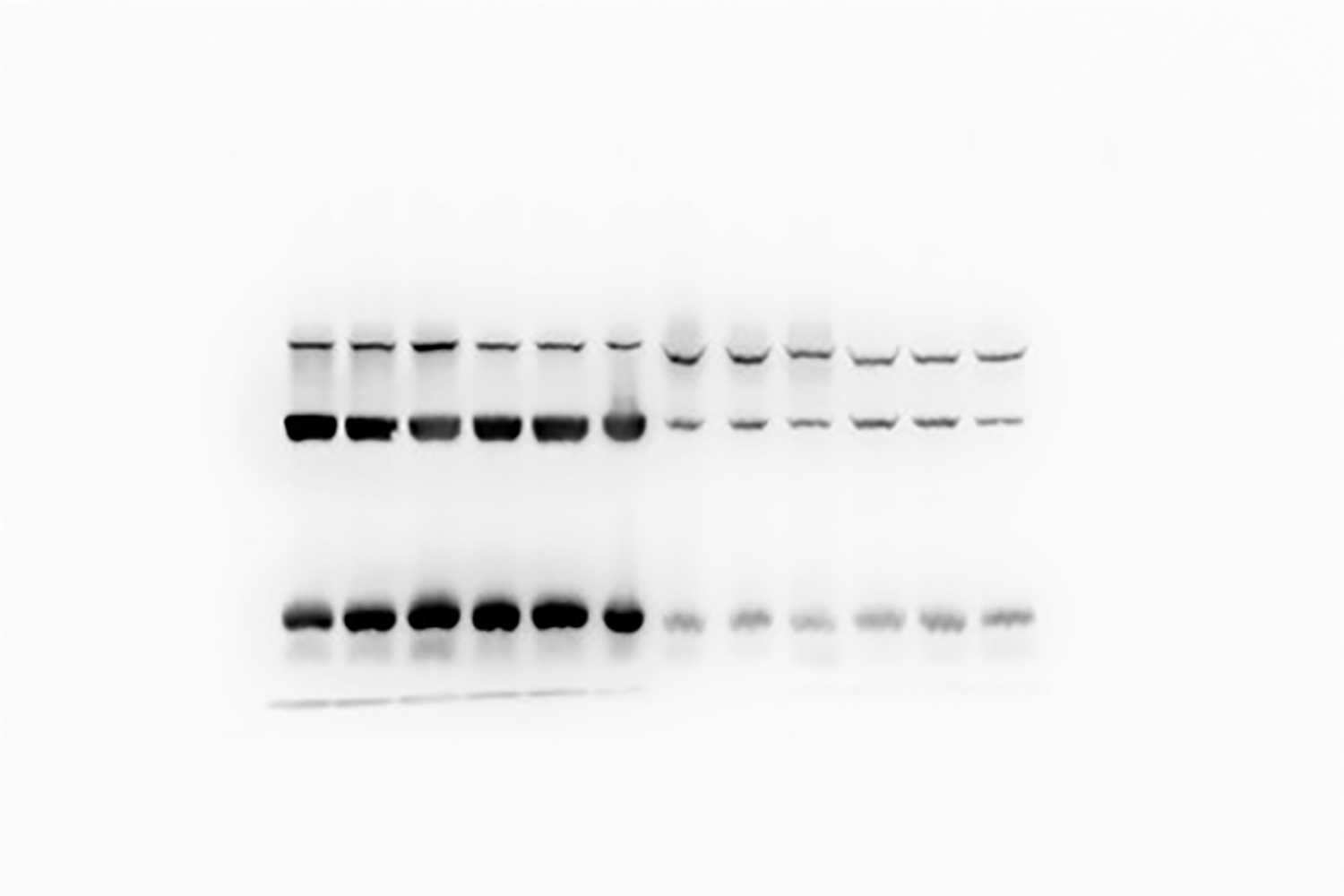

Supplement: Source data 1. [file elife-72266-data1.zip › Source data 1-original files of gels or blots/Figure 2/Figure 2 J OXPHS.jpg]

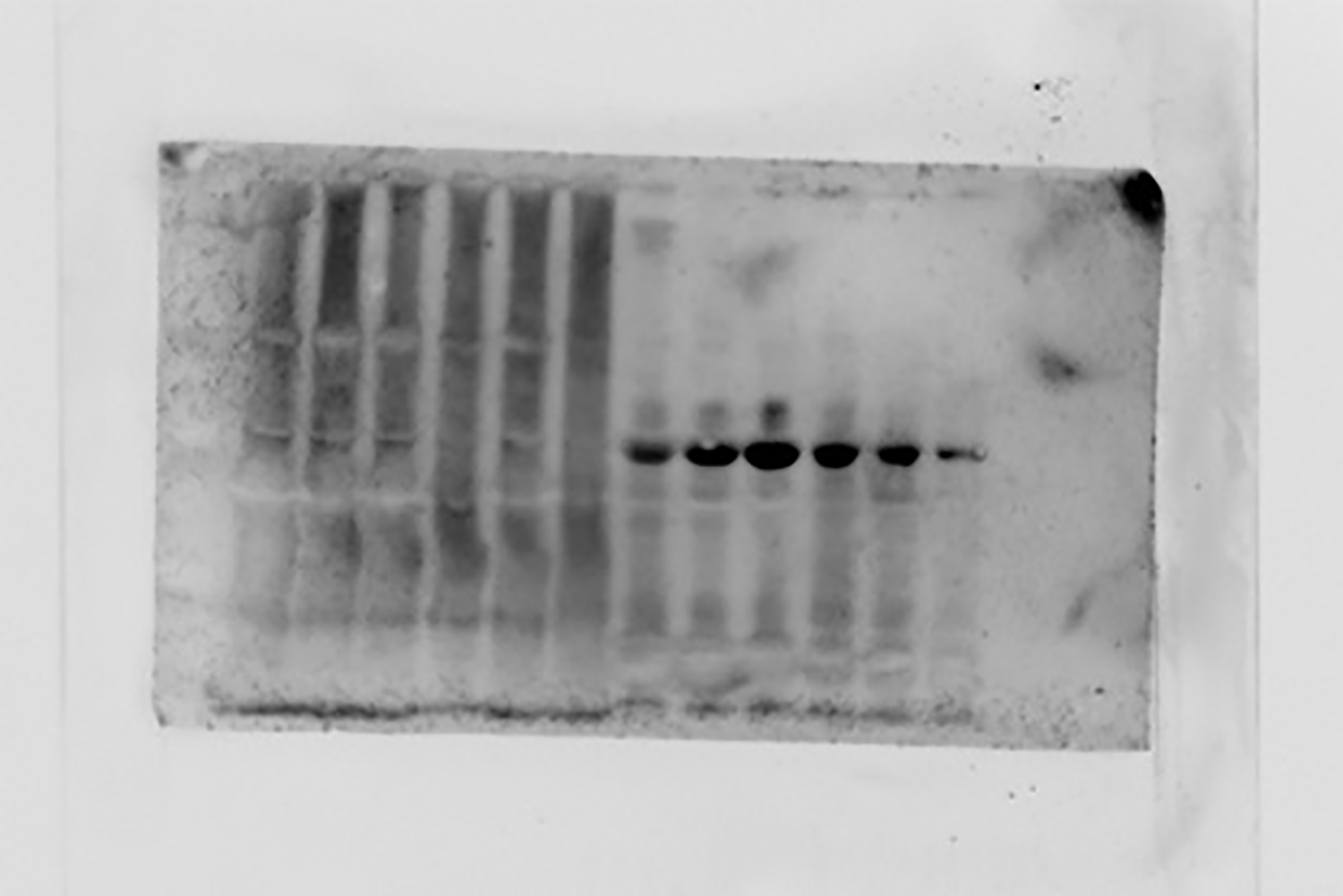

Supplement: Source data 1. [file elife-72266-data1.zip › Source data 1-original files of gels or blots/Figure 2/Figure 2 J UCP1.jpg]

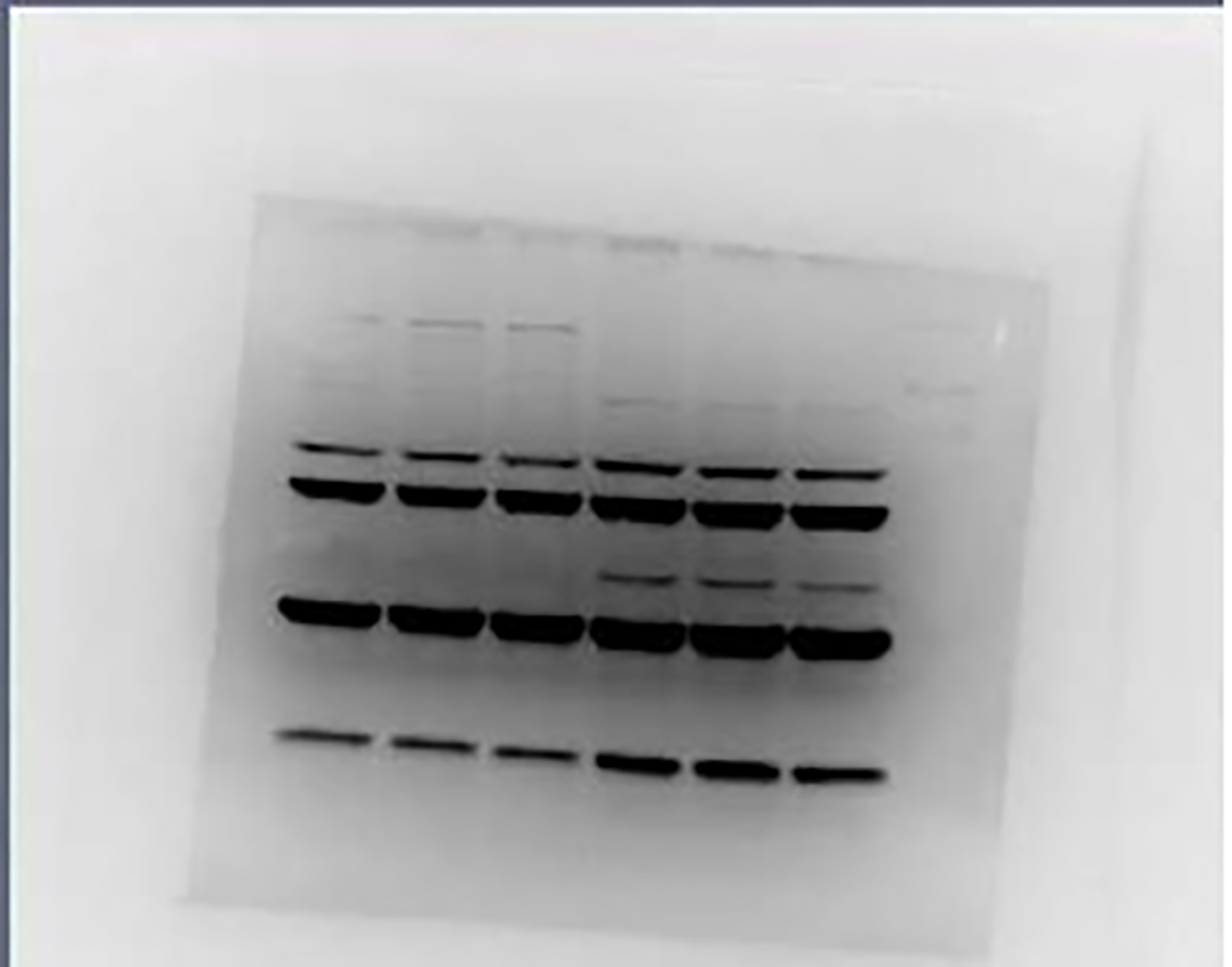

Supplement: Source data 1. [file elife-72266-data1.zip › Source data 1-original files of gels or blots/Figure 2/Figure 2 K OXPHS.jpg]

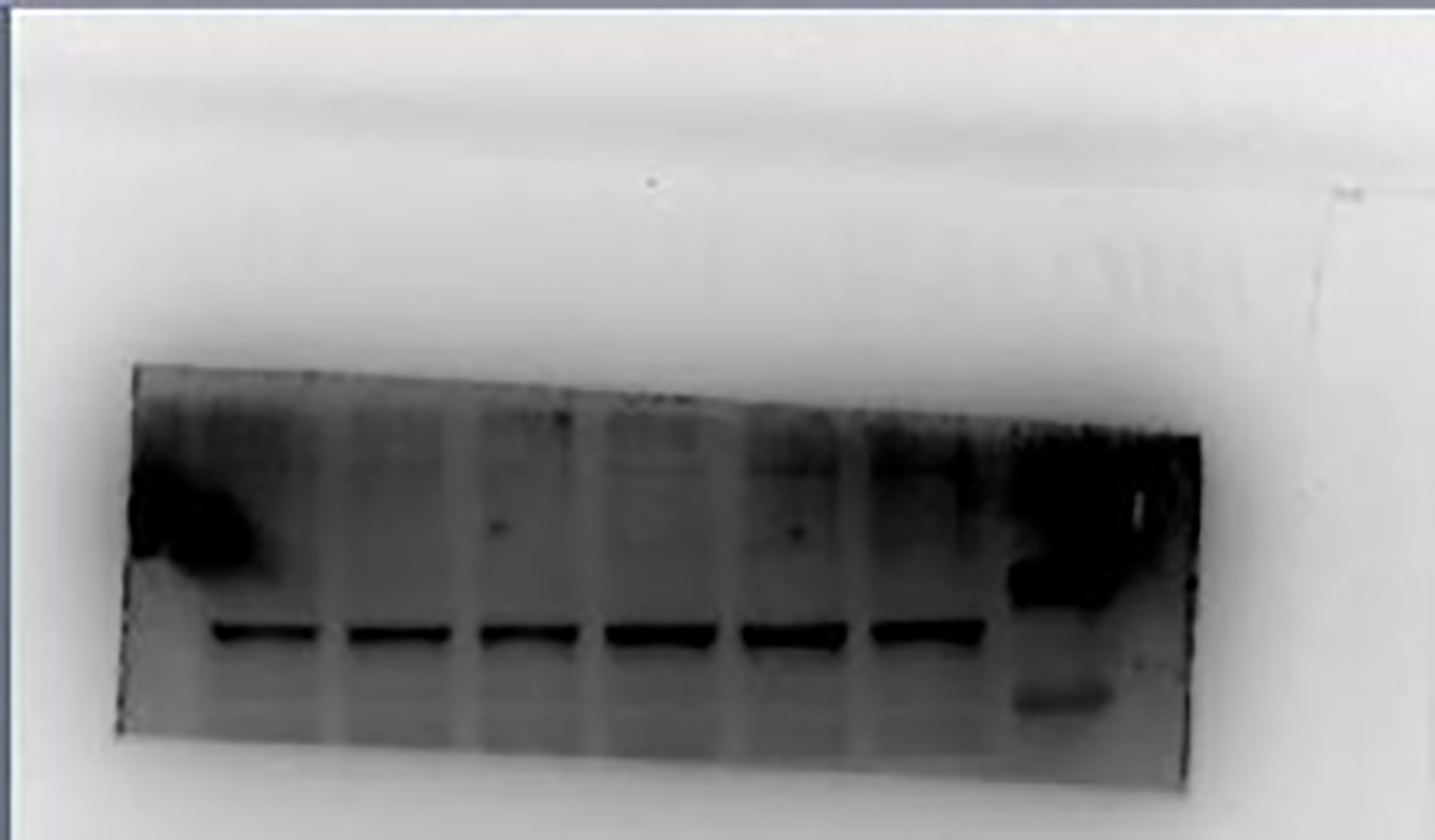

Supplement: Source data 1. [file elife-72266-data1.zip › Source data 1-original files of gels or blots/Figure 2/Figure 2 K PGC-1a.jpg]

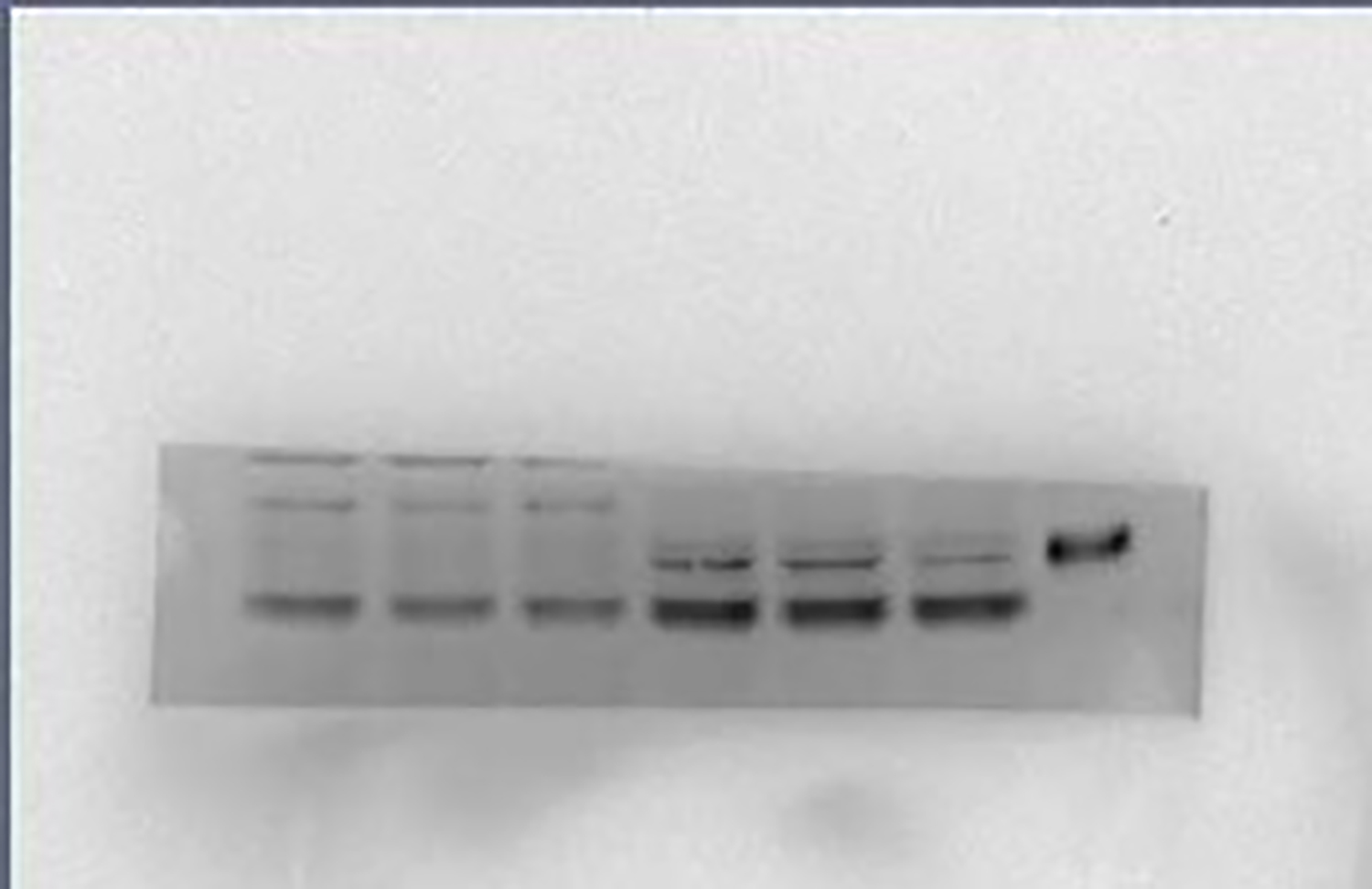

Supplement: Source data 1. [file elife-72266-data1.zip › Source data 1-original files of gels or blots/Figure 2/Figure 2 K UCP1.jpg]

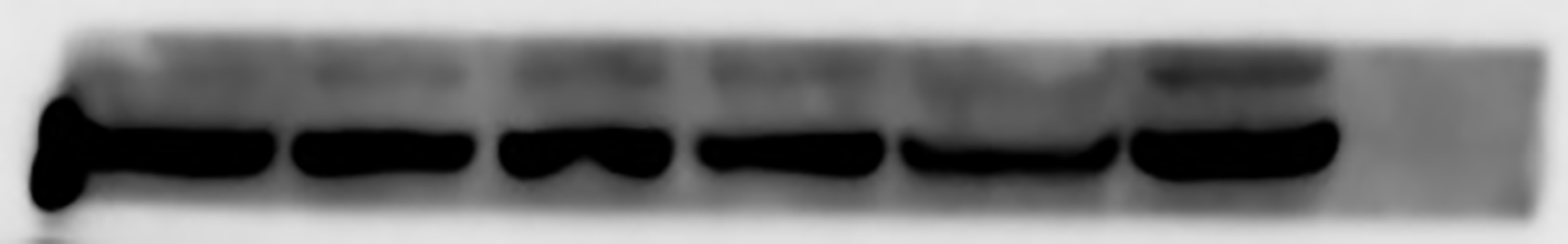

Supplement: Source data 1. [file elife-72266-data1.zip › Source data 1-original files of gels or blots/Figure 3/Figure 3 J ACTIN.jpg]

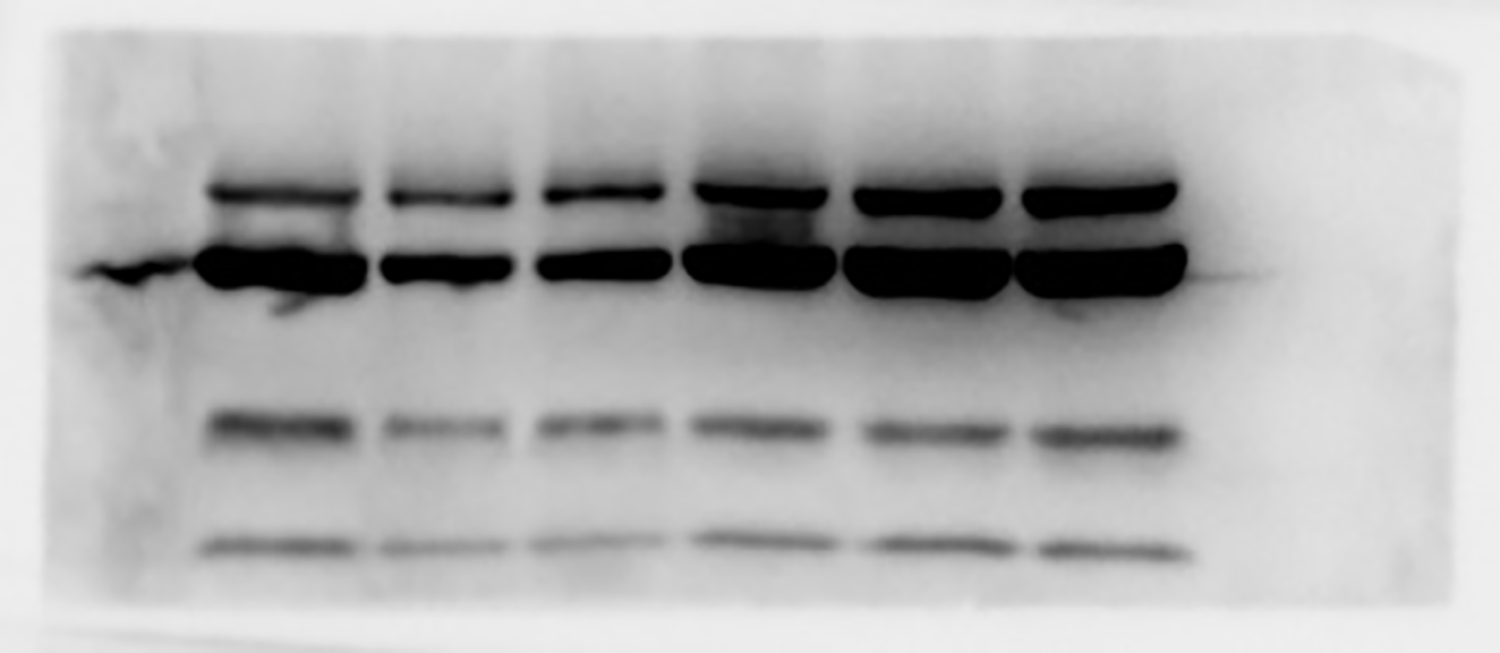

Supplement: Source data 1. [file elife-72266-data1.zip › Source data 1-original files of gels or blots/Figure 3/Figure 3 J OXPHS.jpg]

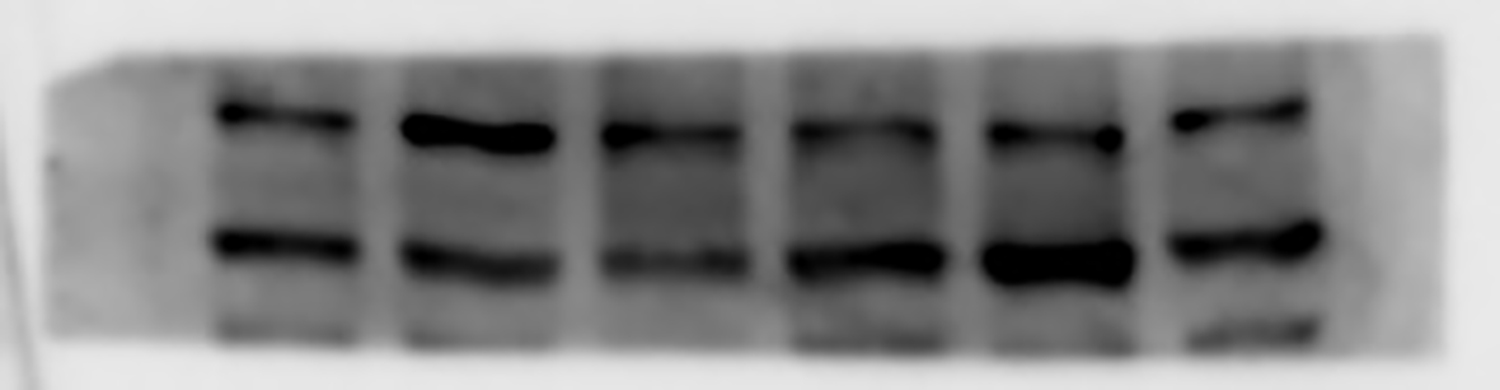

Supplement: Source data 1. [file elife-72266-data1.zip › Source data 1-original files of gels or blots/Figure 3/Figure 3 J PGC1-1a.jpg]

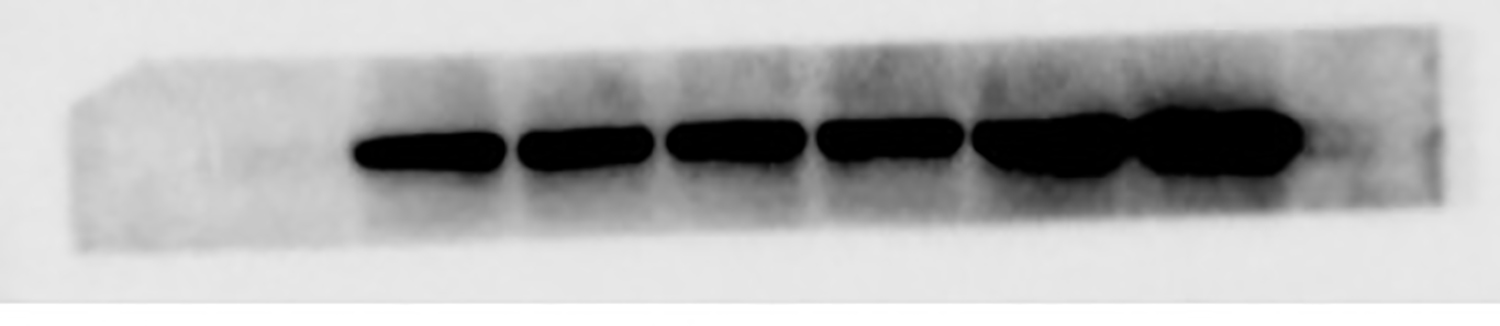

Supplement: Source data 1. [file elife-72266-data1.zip › Source data 1-original files of gels or blots/Figure 3/Figure 3 J UCP1.jpg]

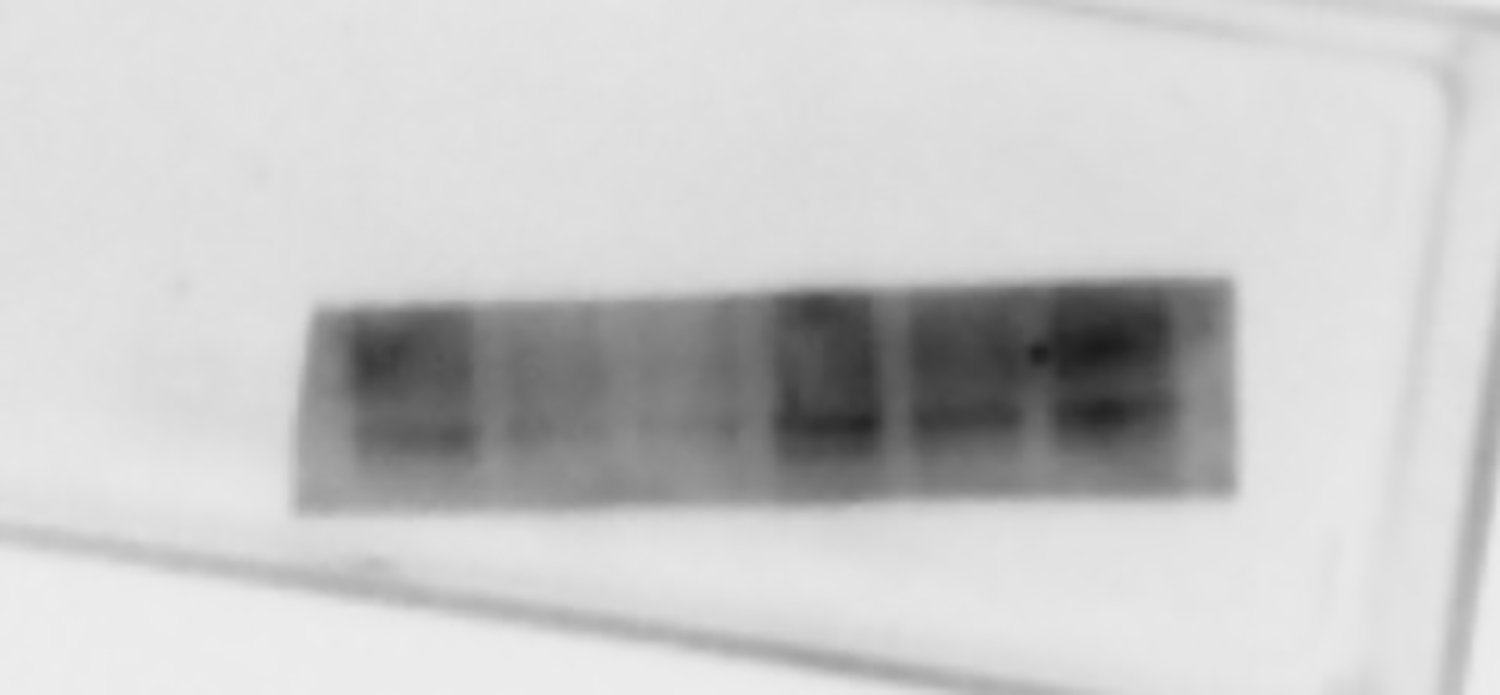

Supplement: Source data 1. [file elife-72266-data1.zip › Source data 1-original files of gels or blots/Figure 3-figure supplement 1/Figure 3-supple-1 A ACE2.jpg]

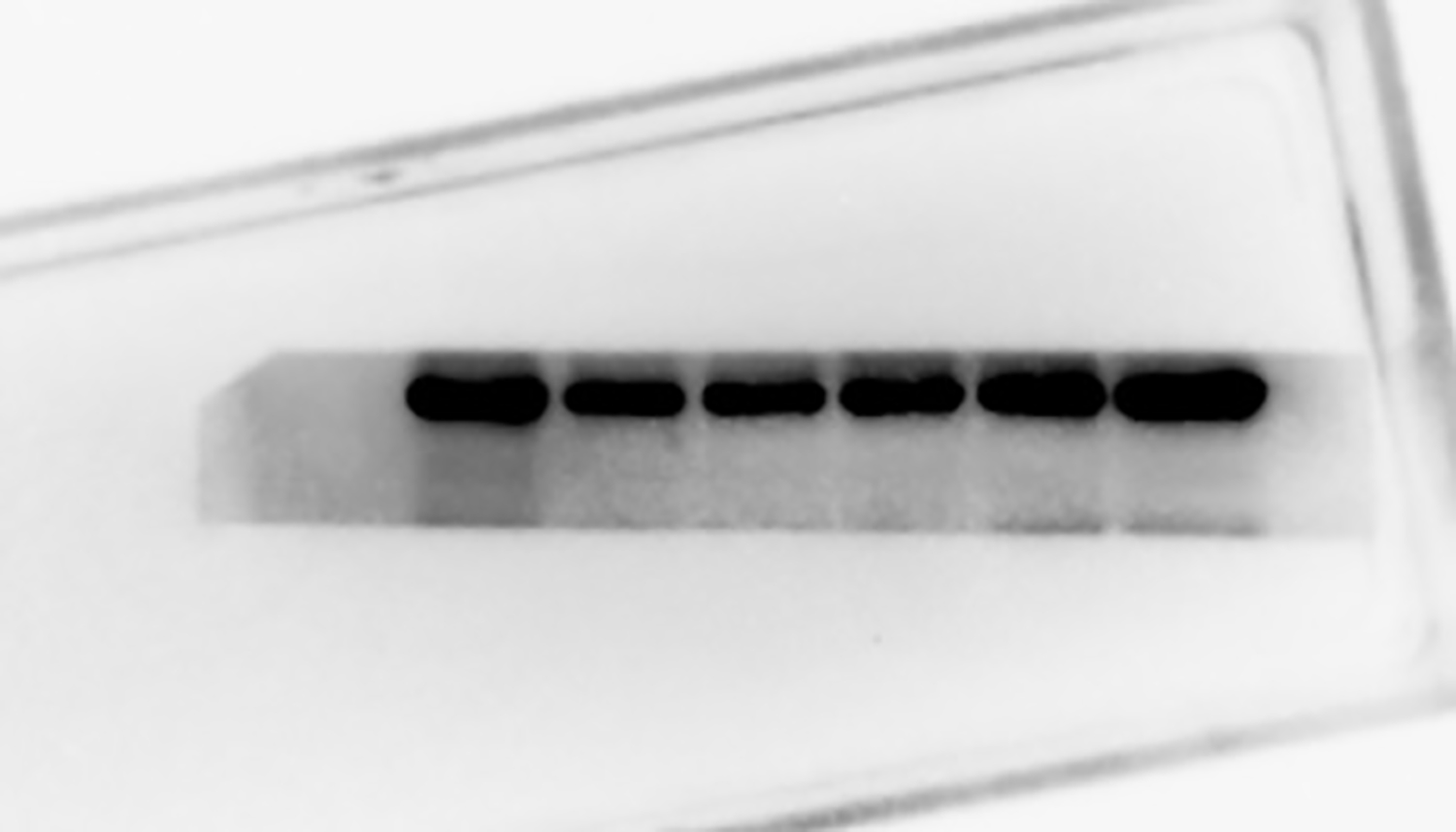

Supplement: Source data 1. [file elife-72266-data1.zip › Source data 1-original files of gels or blots/Figure 3-figure supplement 1/Figure 3-supple-1 A ACTIN.jpg]

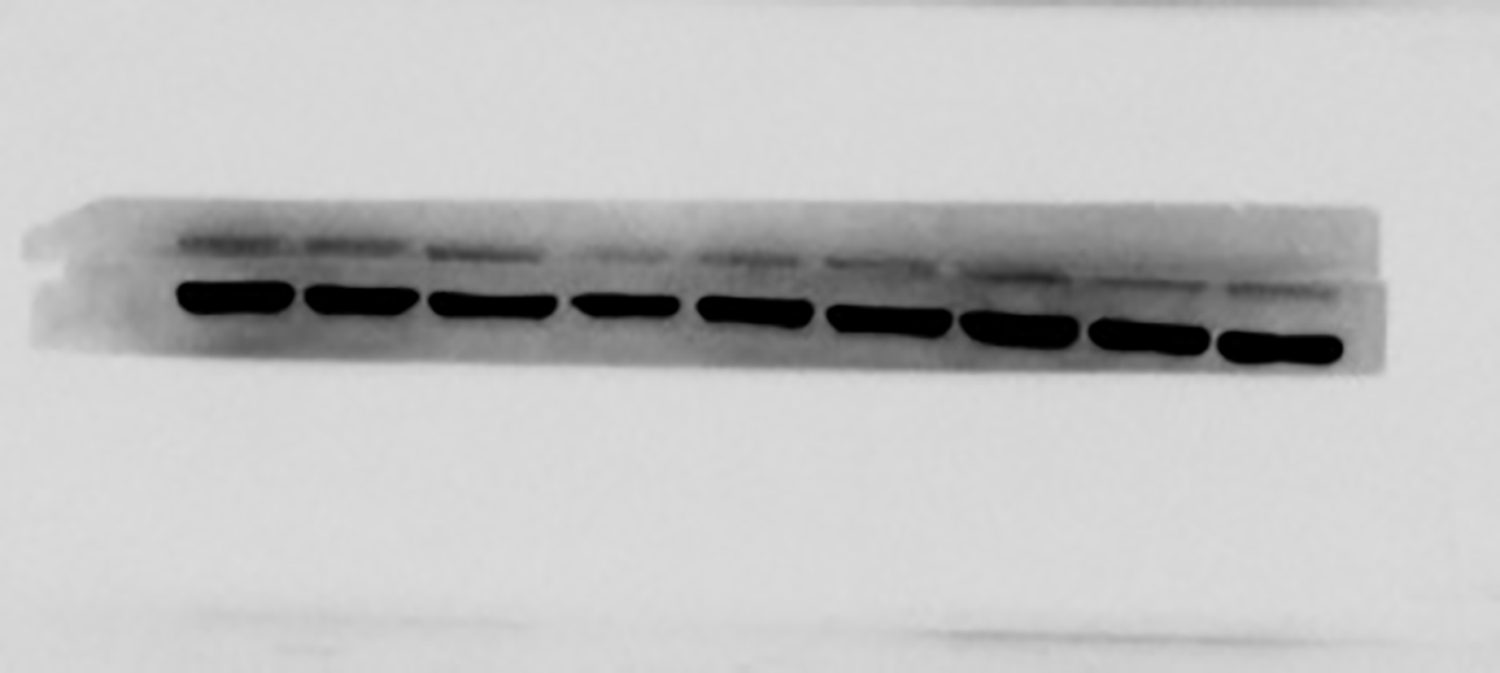

Supplement: Source data 1. [file elife-72266-data1.zip › Source data 1-original files of gels or blots/Figure 4/Figure 4 L ACTIN.jpg]

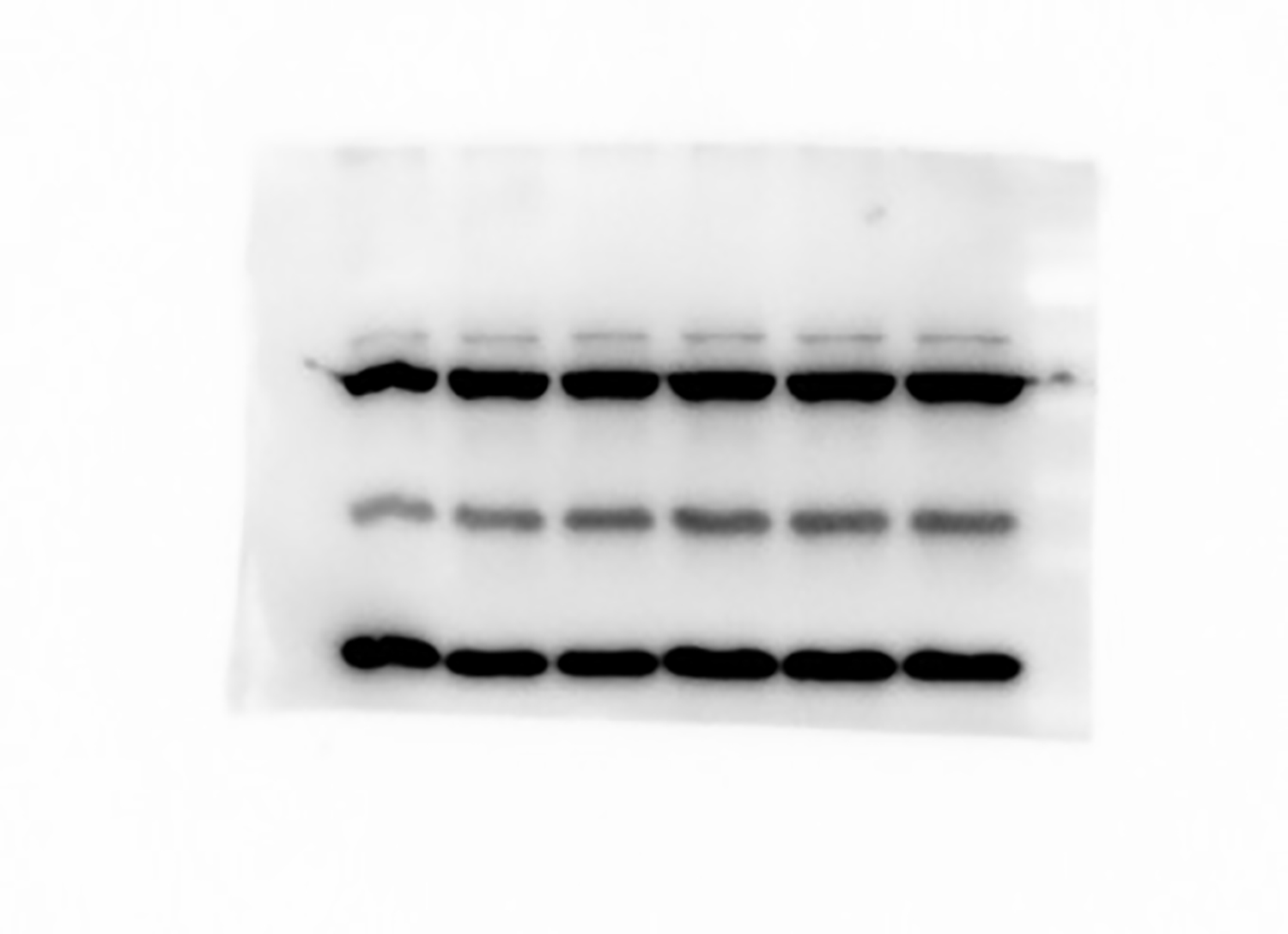

Supplement: Source data 1. [file elife-72266-data1.zip › Source data 1-original files of gels or blots/Figure 4/Figure 4 L OXPHS.jpg]

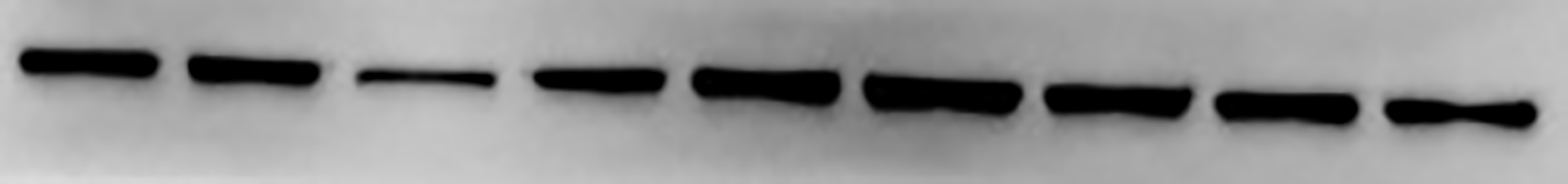

Supplement: Source data 1. [file elife-72266-data1.zip › Source data 1-original files of gels or blots/Figure 4/Figure 4 L PGC-1a.jpg]

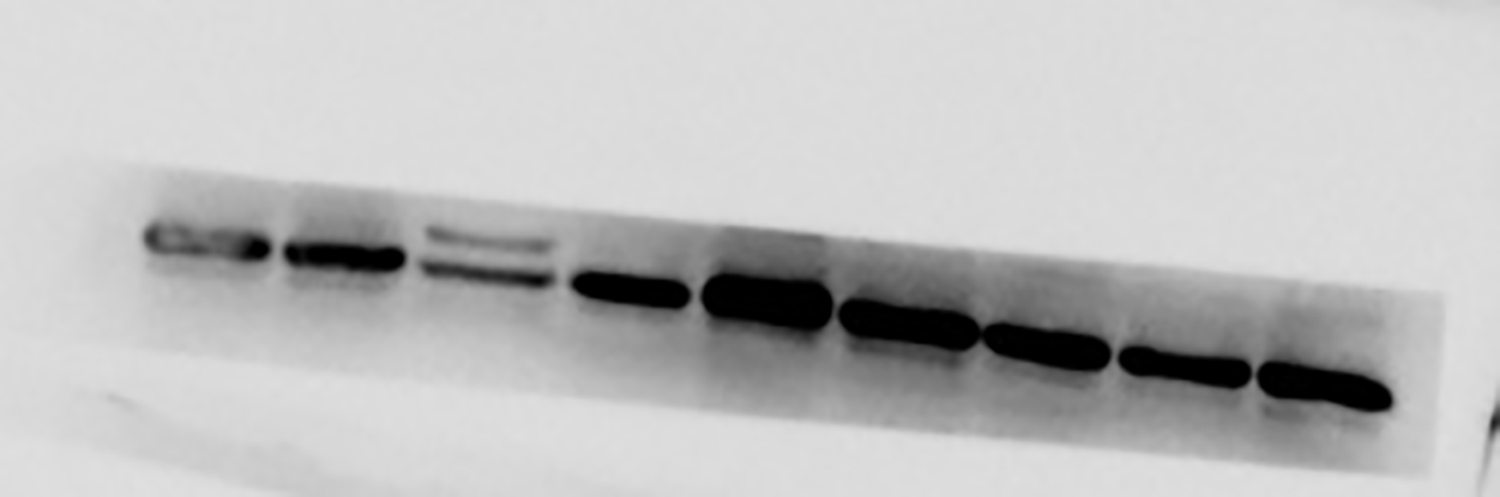

Supplement: Source data 1. [file elife-72266-data1.zip › Source data 1-original files of gels or blots/Figure 4/Figure 4 L UCP1.jpg]

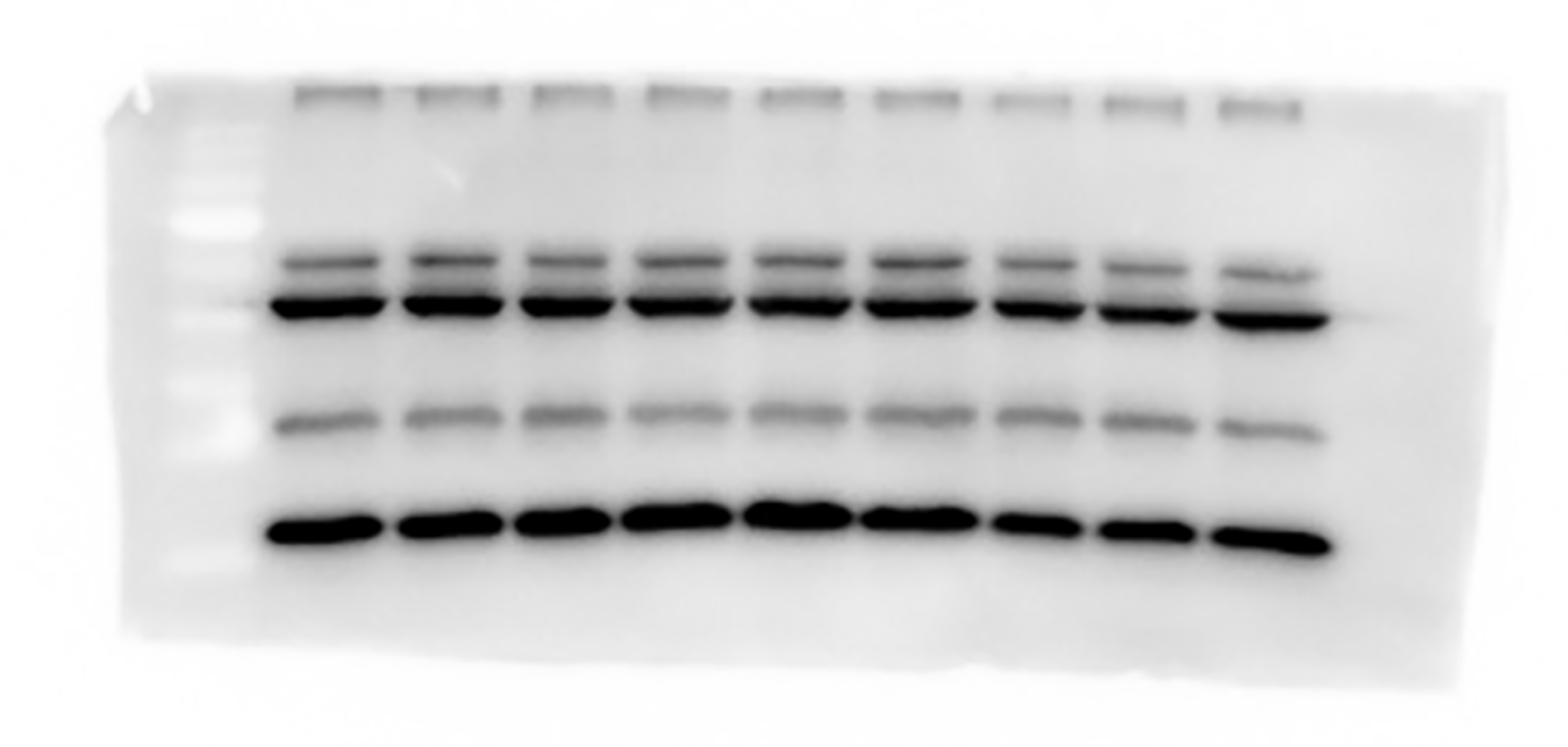

Supplement: Source data 1. [file elife-72266-data1.zip › Source data 1-original files of gels or blots/Figure 4/Figure 4 M ACTIN.jpg]

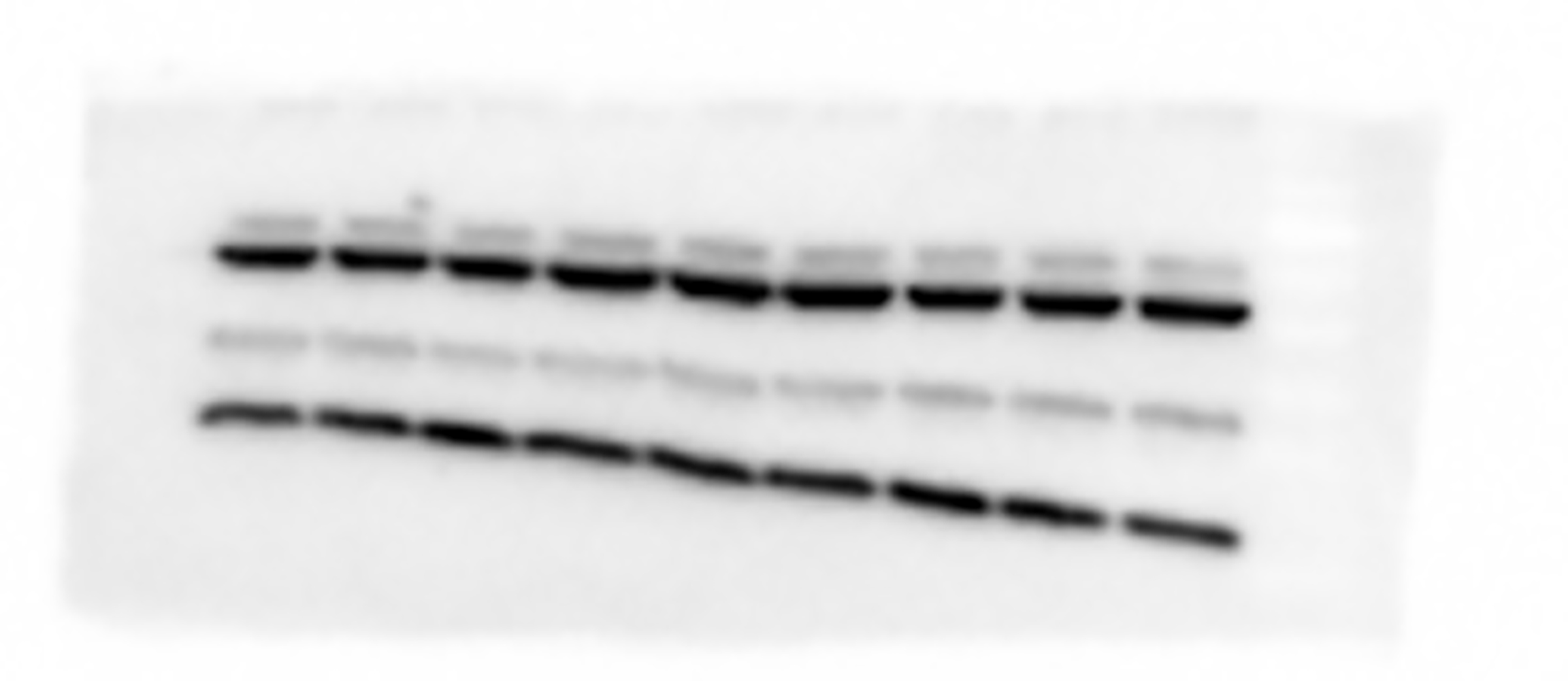

Supplement: Source data 1. [file elife-72266-data1.zip › Source data 1-original files of gels or blots/Figure 4/Figure 4 M OXPHS.jpg]

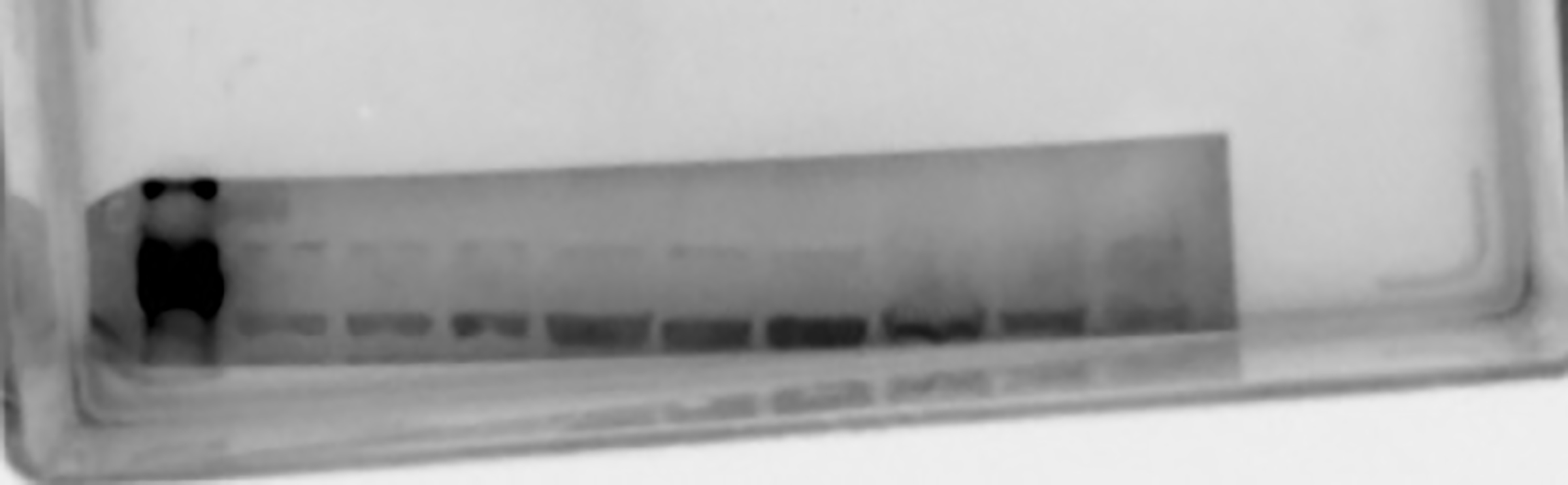

Supplement: Source data 1. [file elife-72266-data1.zip › Source data 1-original files of gels or blots/Figure 4/Figure 4 M PGC-1a.jpg]

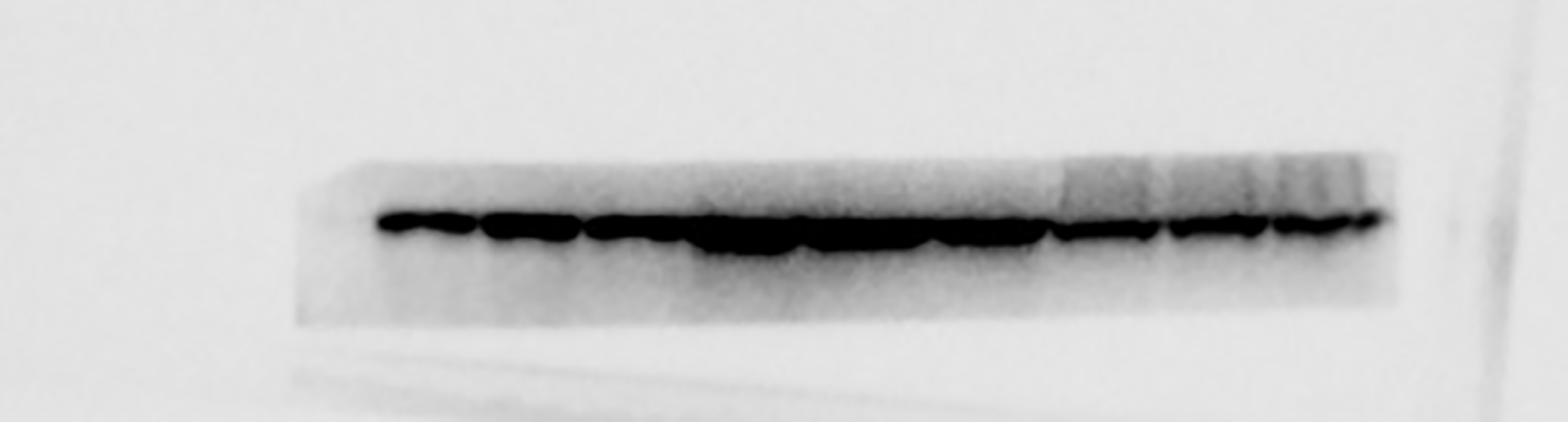

Supplement: Source data 1. [file elife-72266-data1.zip › Source data 1-original files of gels or blots/Figure 4/Figure 4 M UCP1.jpg]

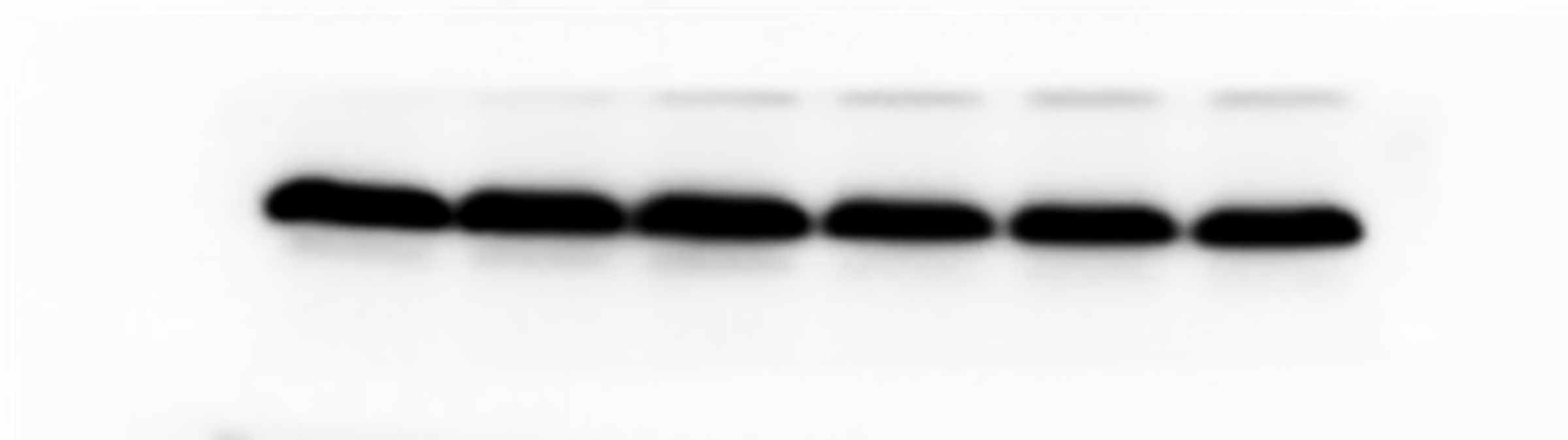

Supplement: Source data 1. [file elife-72266-data1.zip › Source data 1-original files of gels or blots/Figure 5/Figure 5 O ACTIN.jpg]

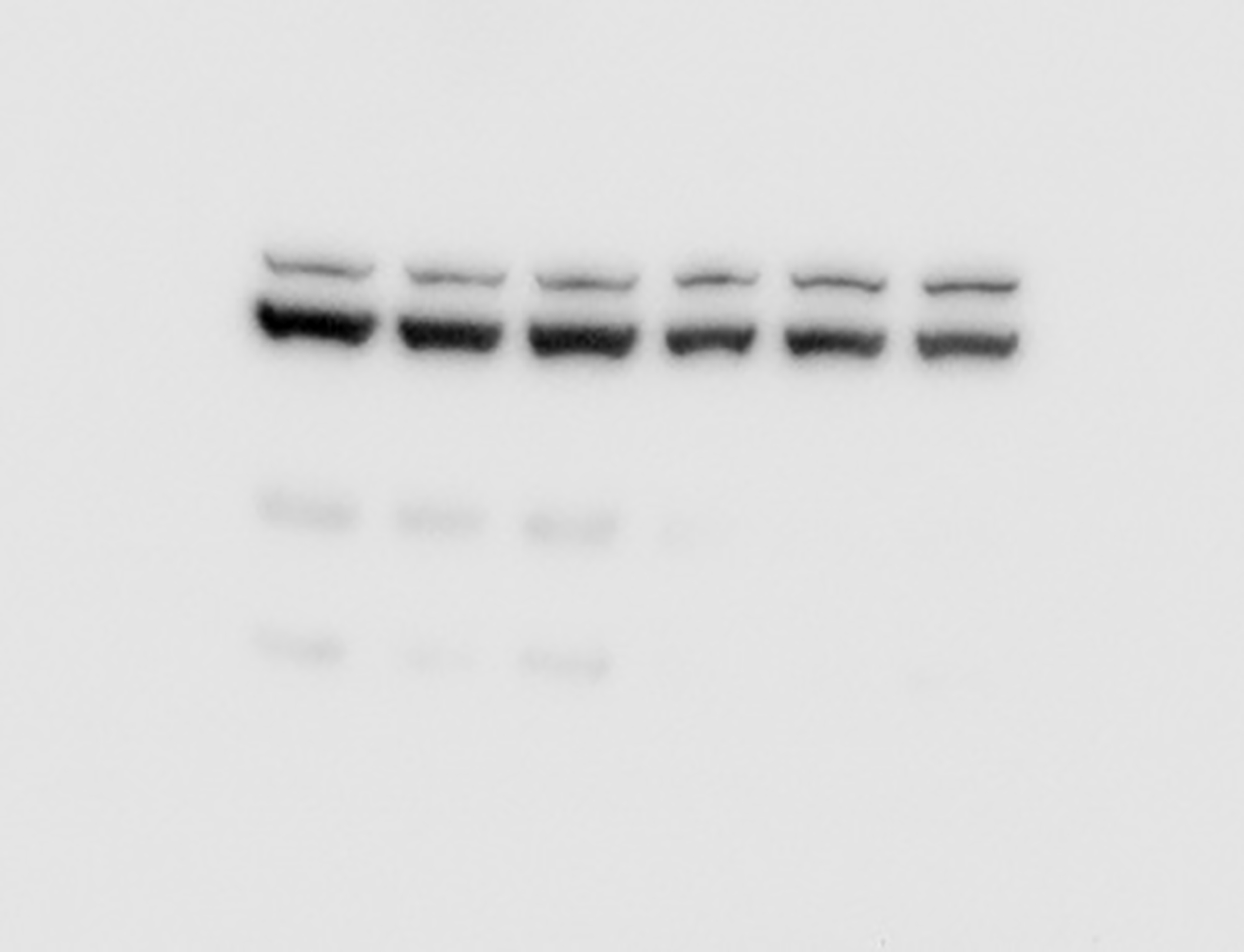

Supplement: Source data 1. [file elife-72266-data1.zip › Source data 1-original files of gels or blots/Figure 5/Figure 5 O OXPHS.jpg]

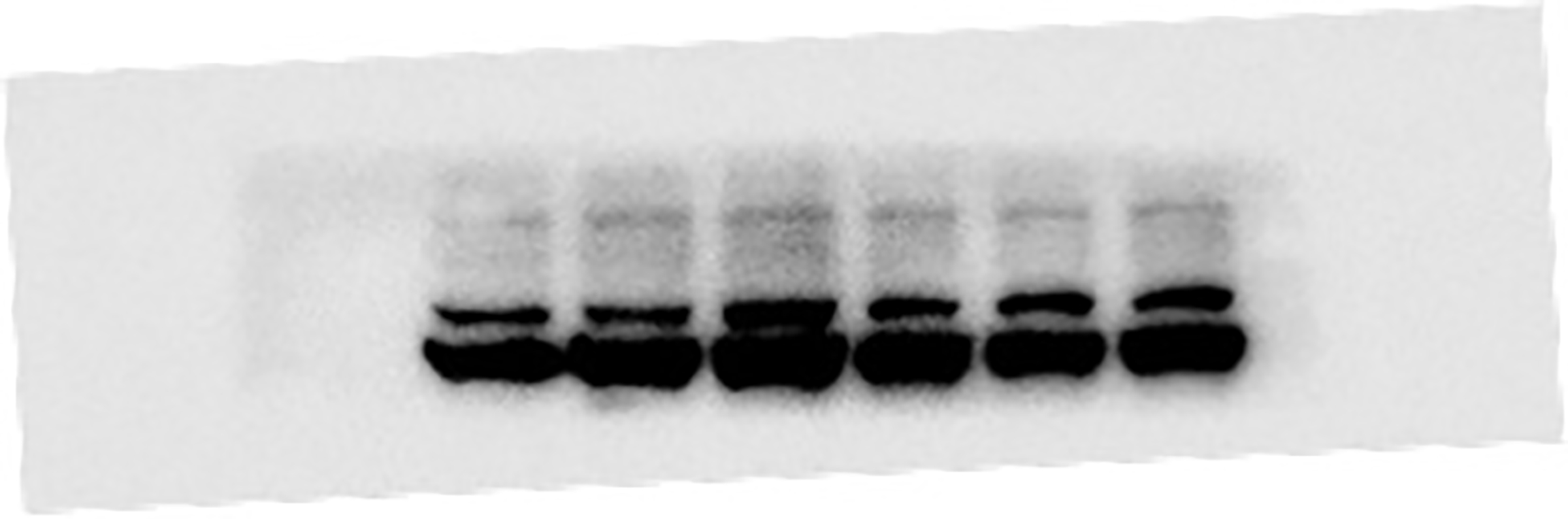

Supplement: Source data 1. [file elife-72266-data1.zip › Source data 1-original files of gels or blots/Figure 5/Figure 5 O PGC-1a.jpg]

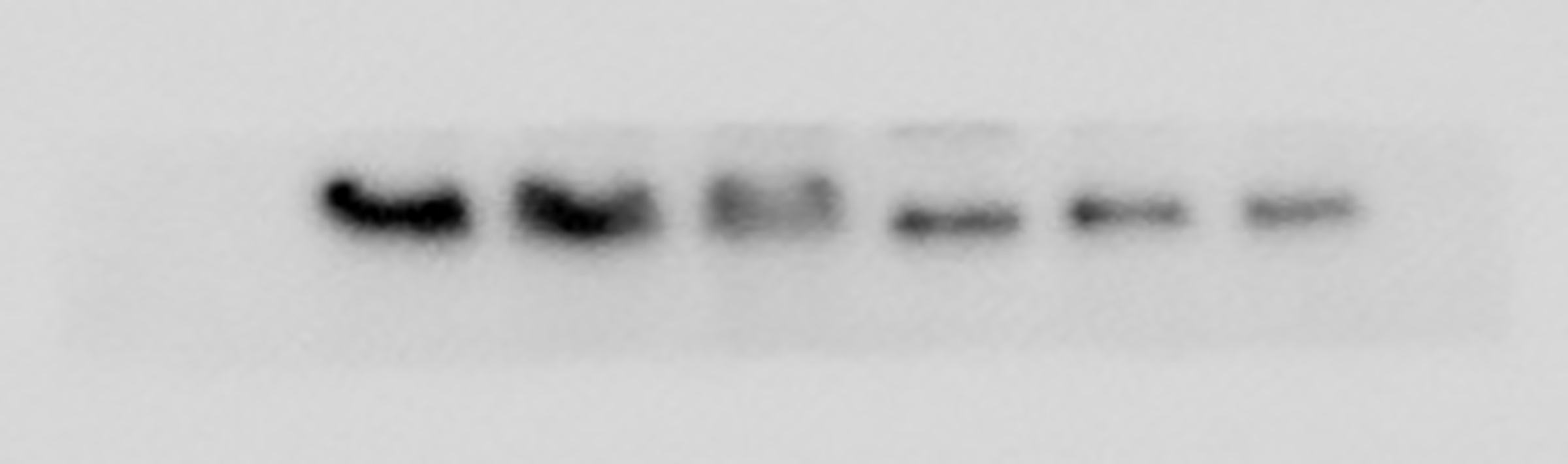

Supplement: Source data 1. [file elife-72266-data1.zip › Source data 1-original files of gels or blots/Figure 5/Figure 5 O UCP1.jpg]

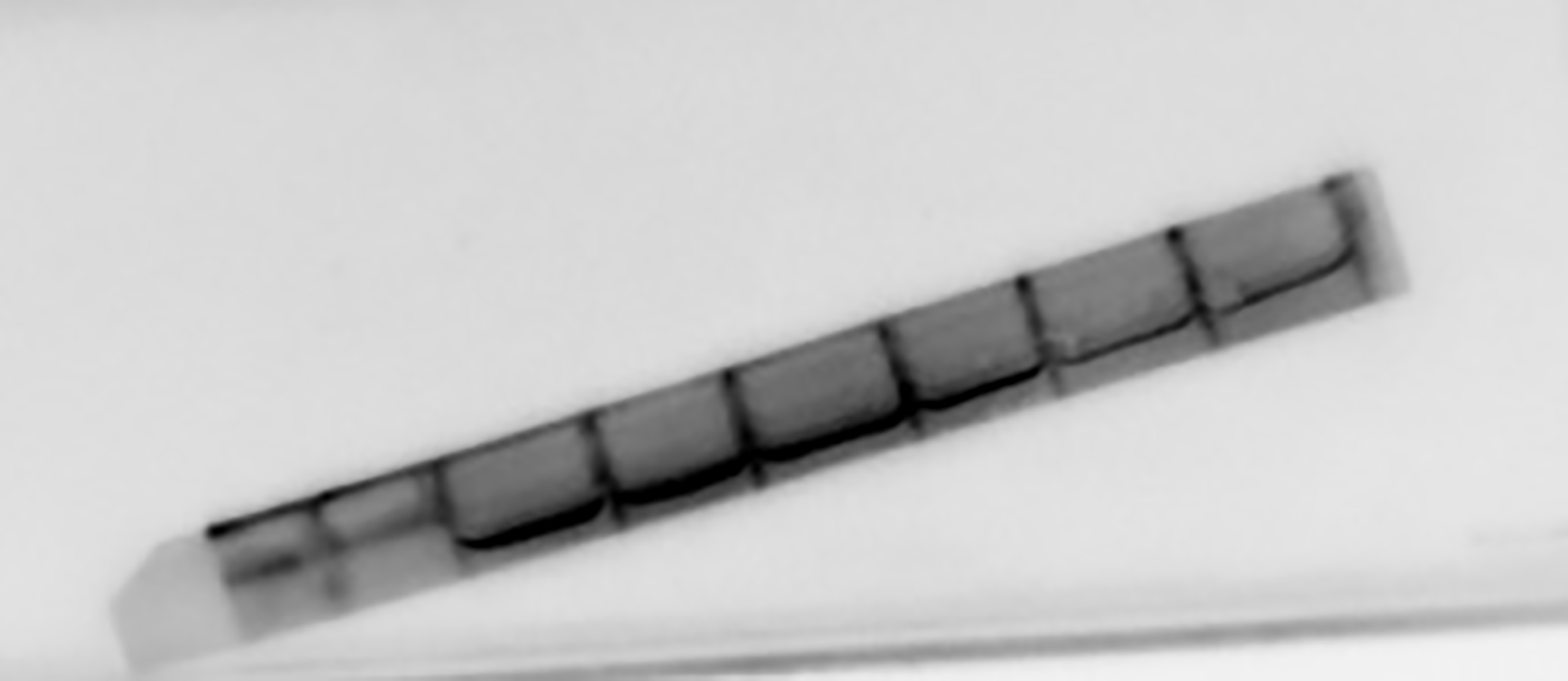

Supplement: Source data 1. [file elife-72266-data1.zip › Source data 1-original files of gels or blots/Figure 7/Figure 7 A pAKT308.jpg]

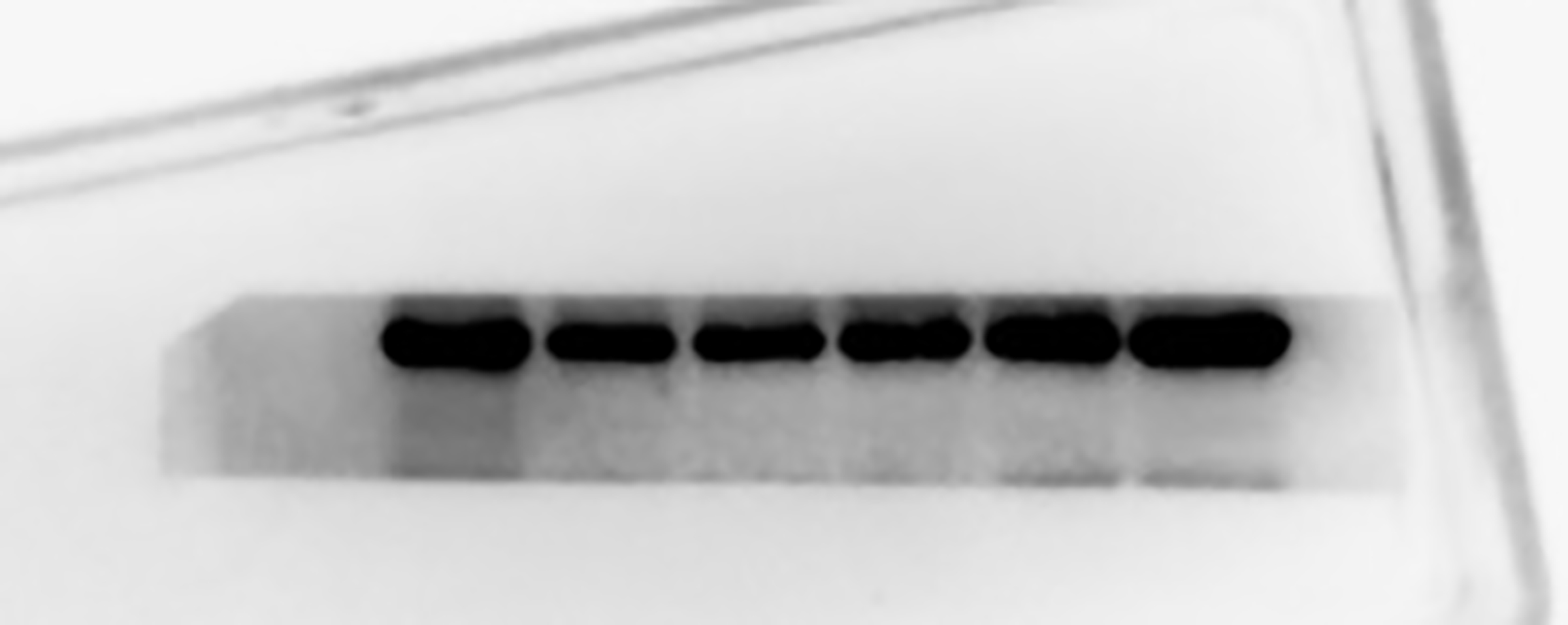

Supplement: Source data 1. [file elife-72266-data1.zip › Source data 1-original files of gels or blots/Figure 7/Figure 7 B ACTIN.jpg]

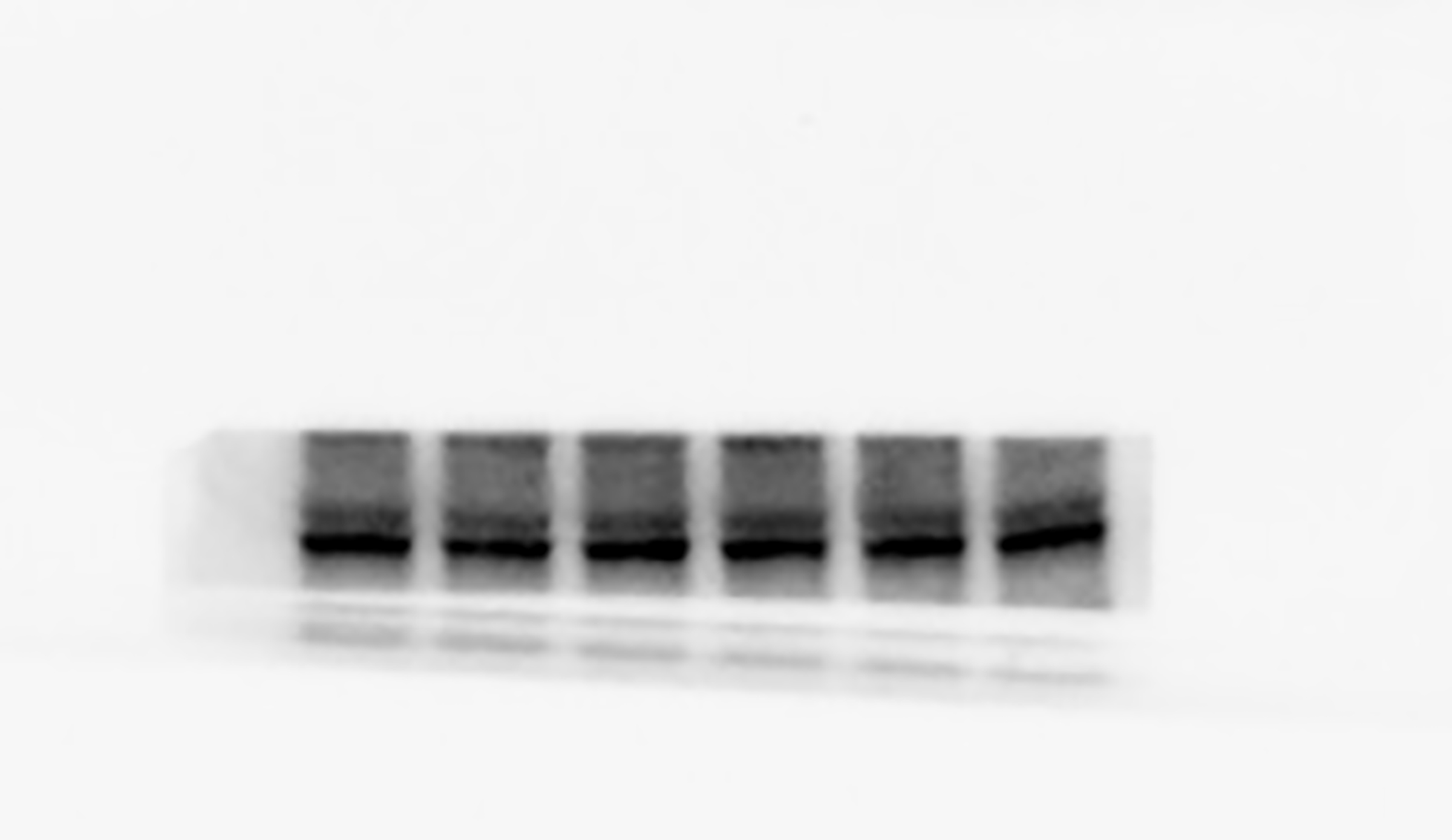

Supplement: Source data 1. [file elife-72266-data1.zip › Source data 1-original files of gels or blots/Figure 7/Figure 7 B AKT.jpg]

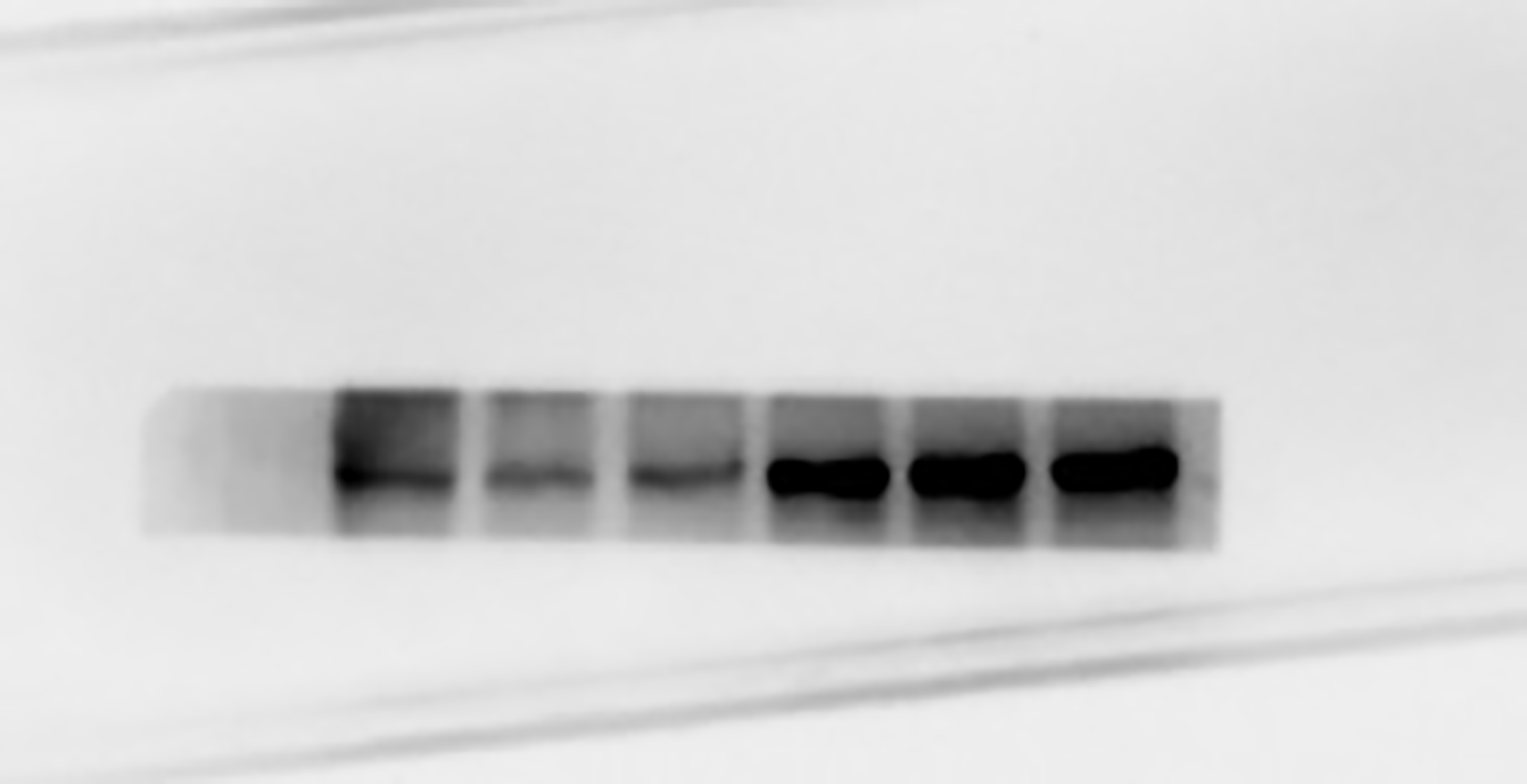

Supplement: Source data 1. [file elife-72266-data1.zip › Source data 1-original files of gels or blots/Figure 7/Figure 7 B pAKT308.jpg]

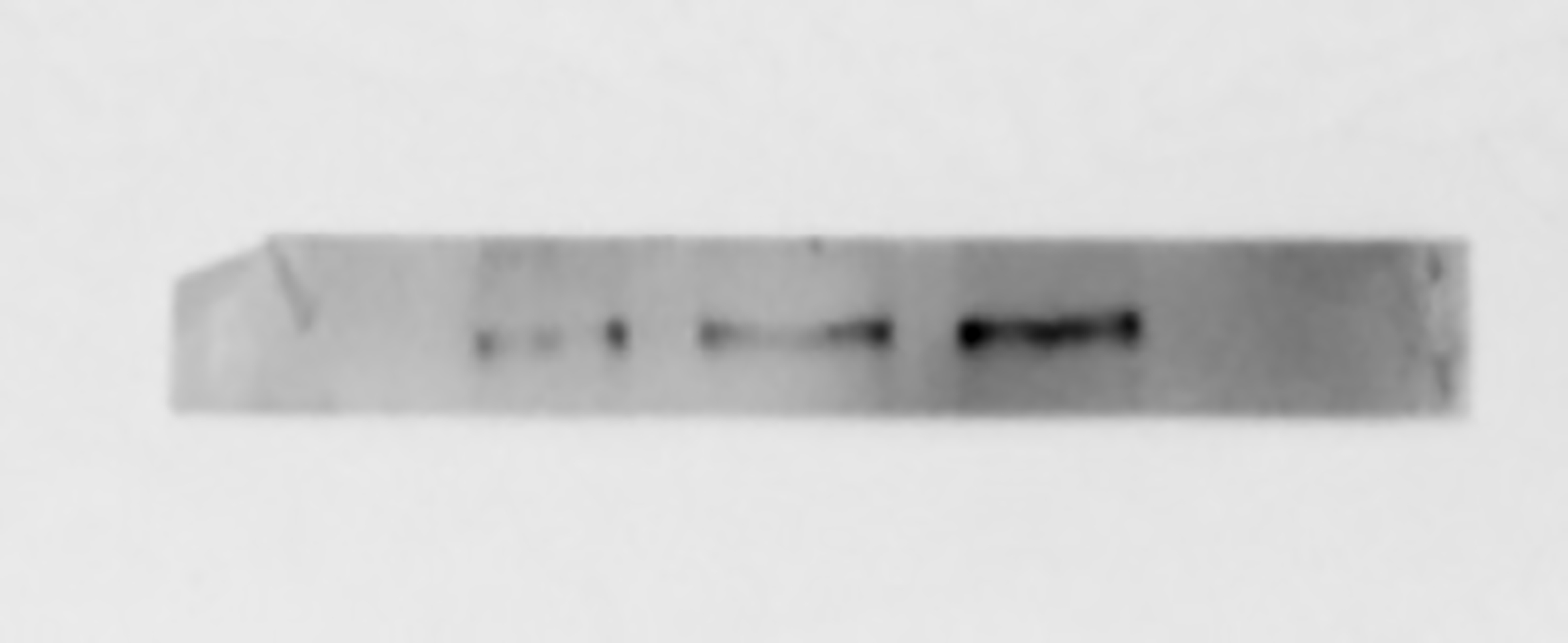

Supplement: Source data 1. [file elife-72266-data1.zip › Source data 1-original files of gels or blots/Figure 7/Figure 7 C AKT.jpg]

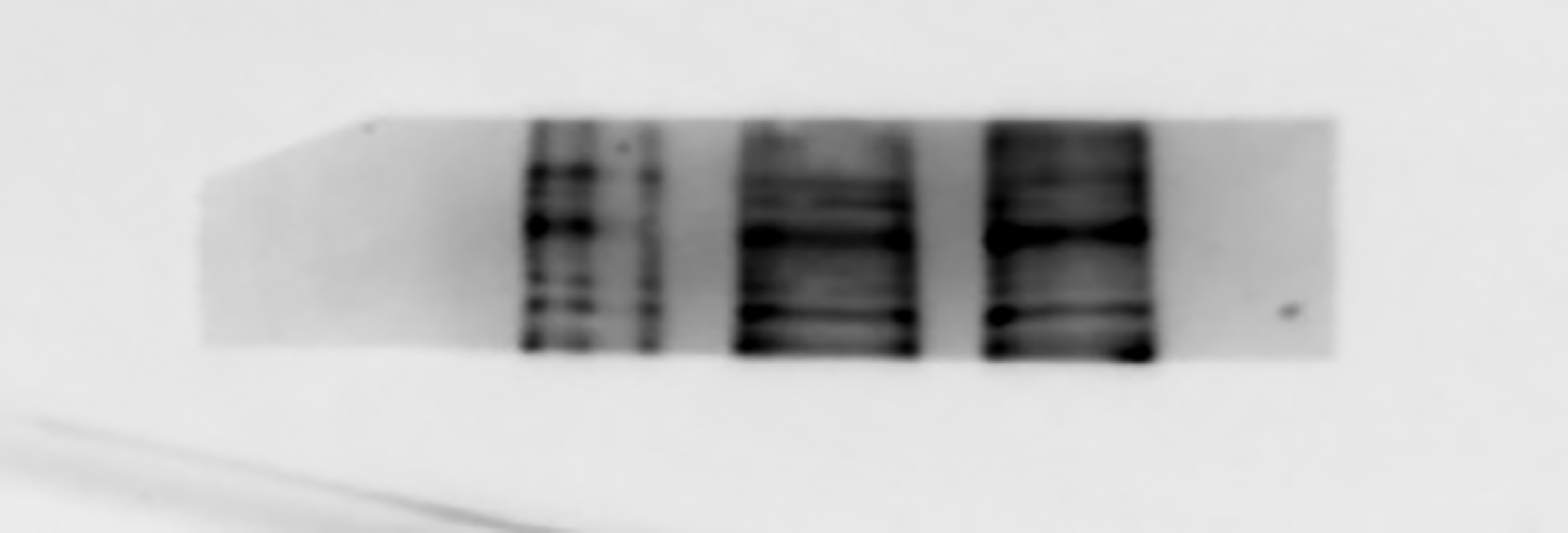

Supplement: Source data 1. [file elife-72266-data1.zip › Source data 1-original files of gels or blots/Figure 7/Figure 7 C FOXO1.jpg]

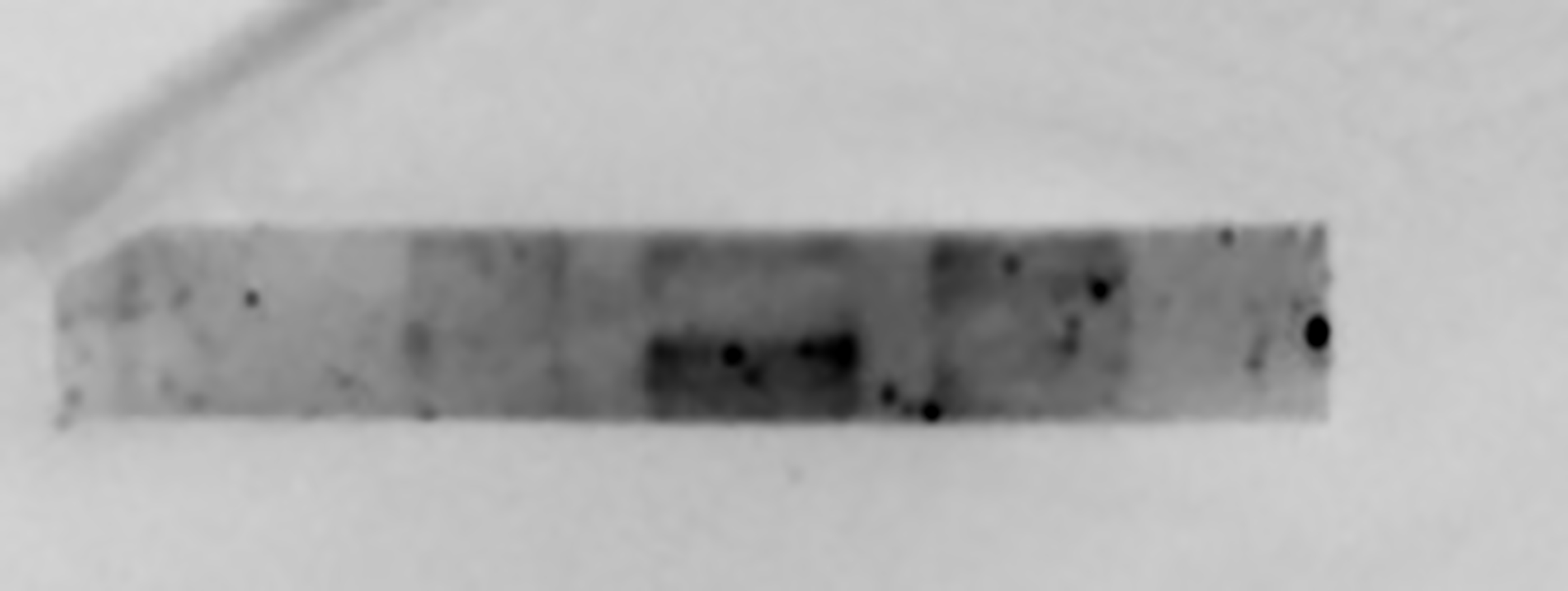

Supplement: Source data 1. [file elife-72266-data1.zip › Source data 1-original files of gels or blots/Figure 7/Figure 7 C pAKT308.jpg]

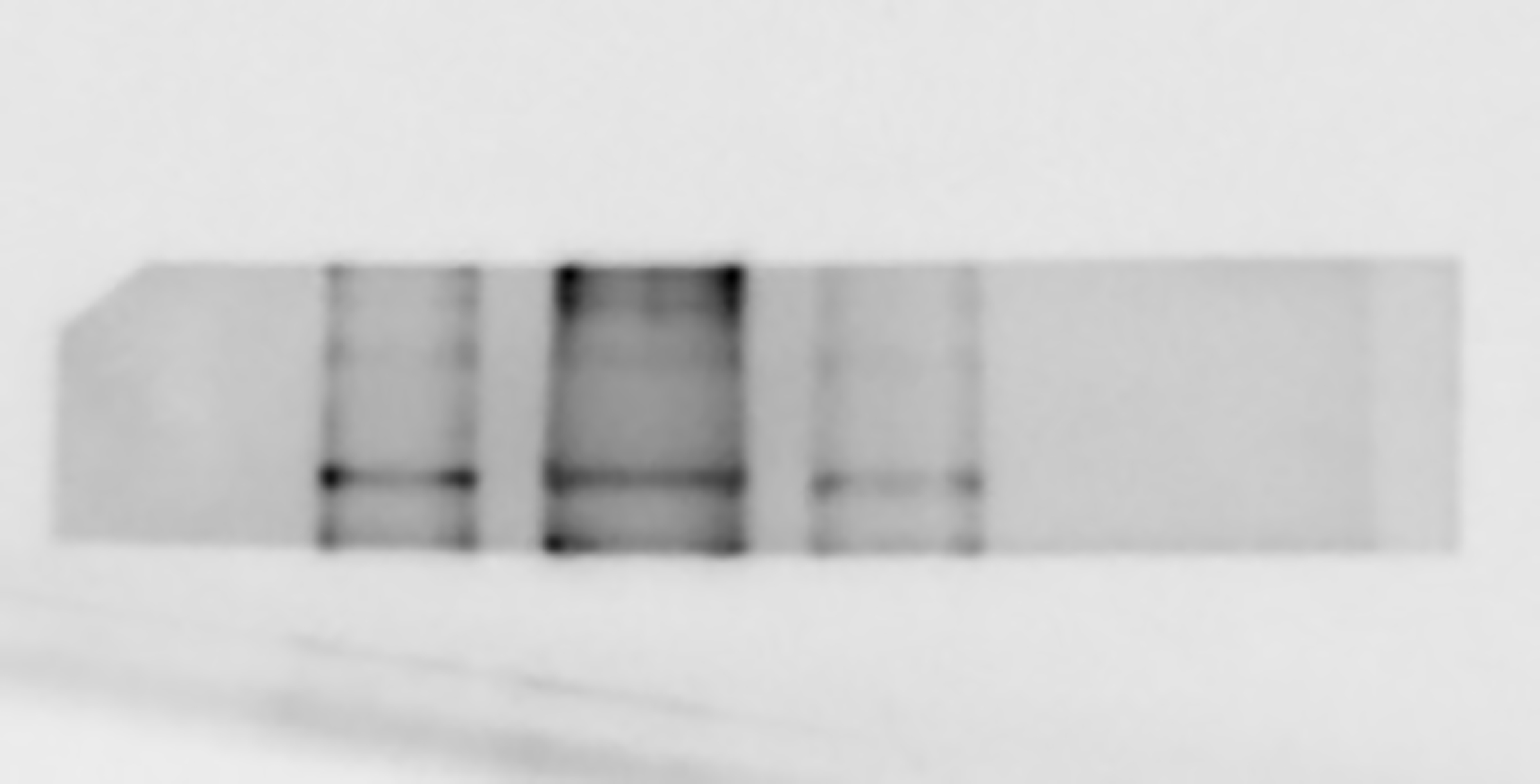

Supplement: Source data 1. [file elife-72266-data1.zip › Source data 1-original files of gels or blots/Figure 7/Figure 7 C pFOXO1.jpg]

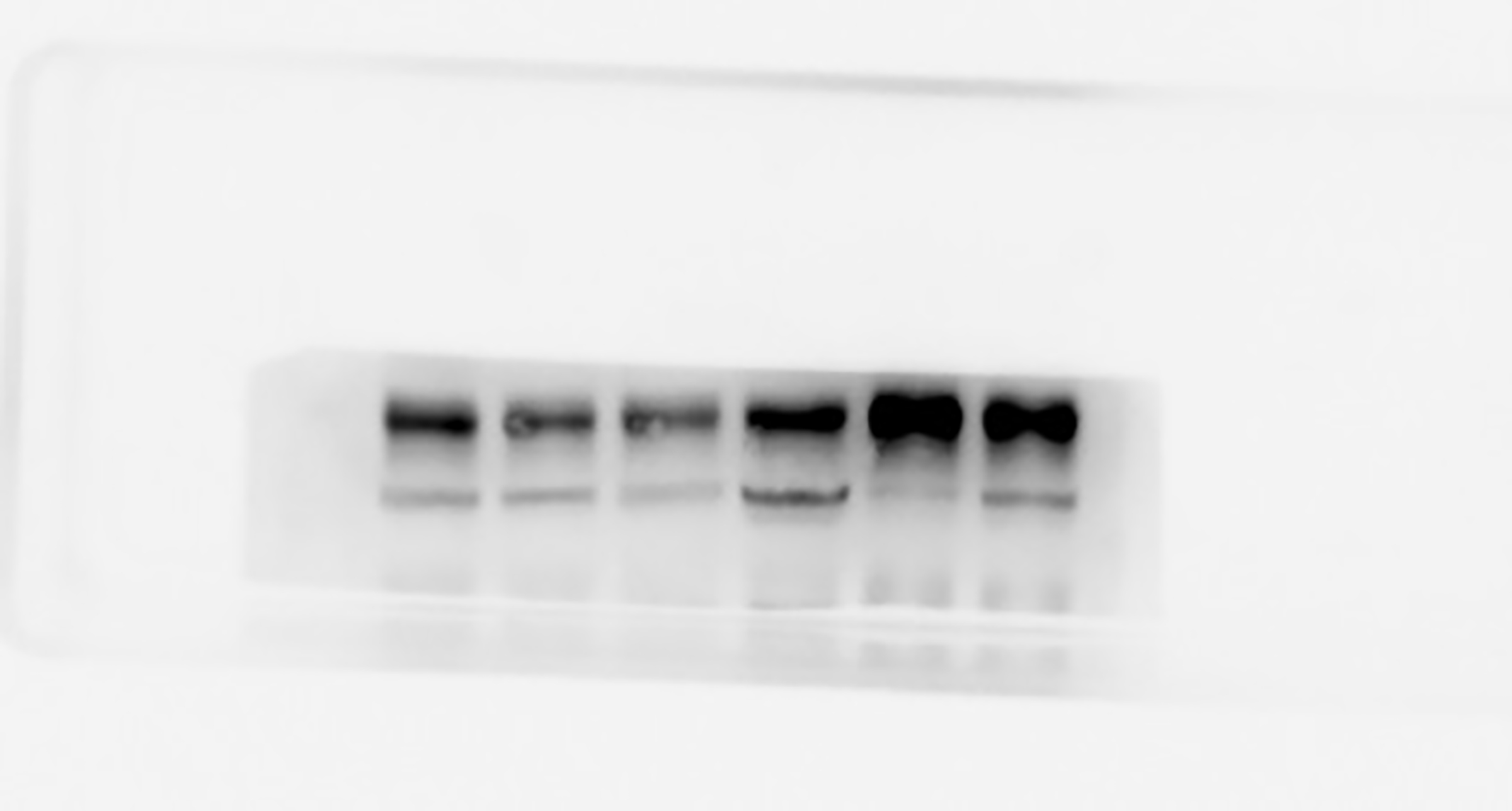

Supplement: Source data 1. [file elife-72266-data1.zip › Source data 1-original files of gels or blots/Figure 7/Figure 7 F PKA.jpg]

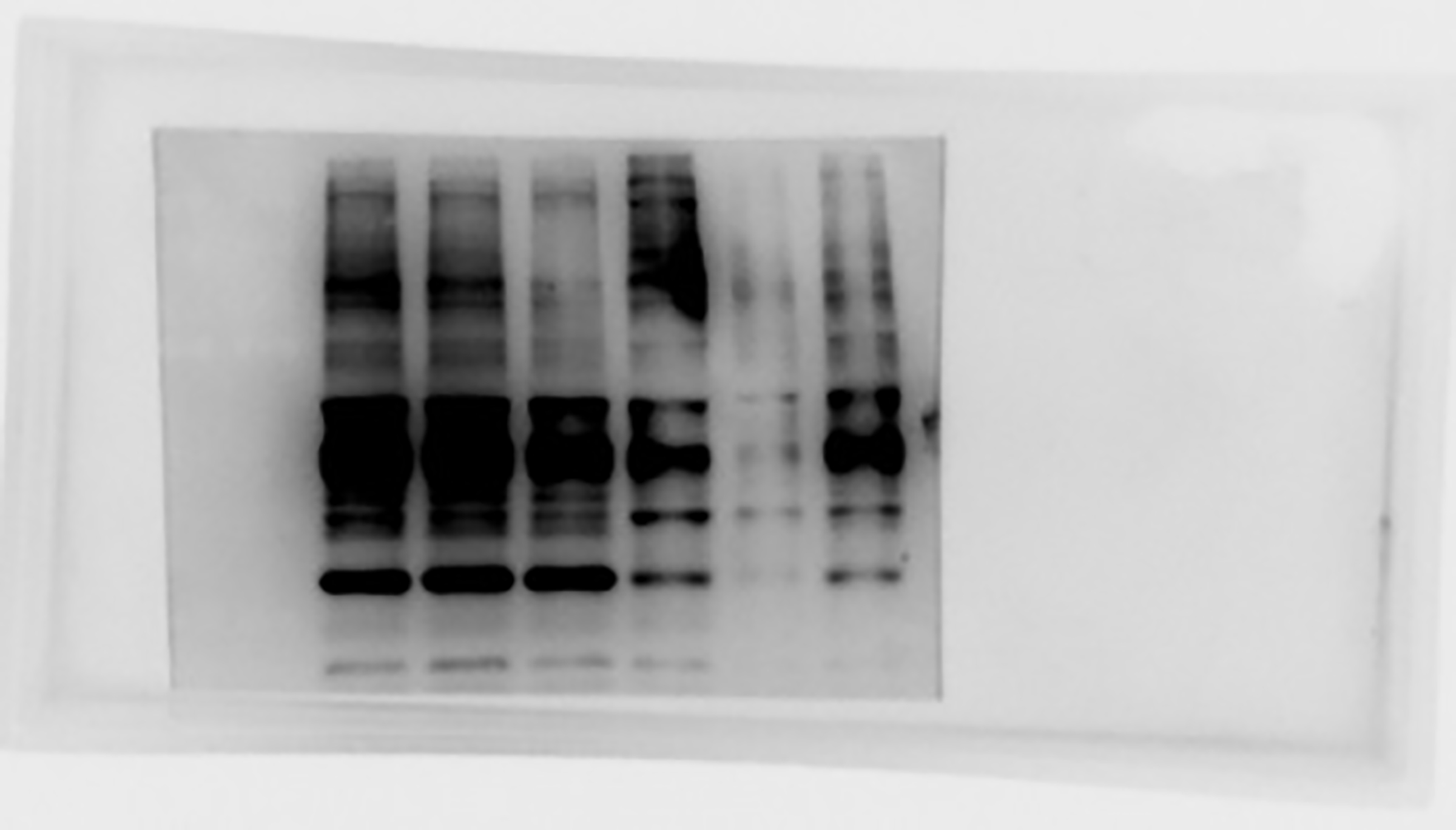

Supplement: Source data 1. [file elife-72266-data1.zip › Source data 1-original files of gels or blots/Figure 7/Figure 7 F pPKA.jpg]

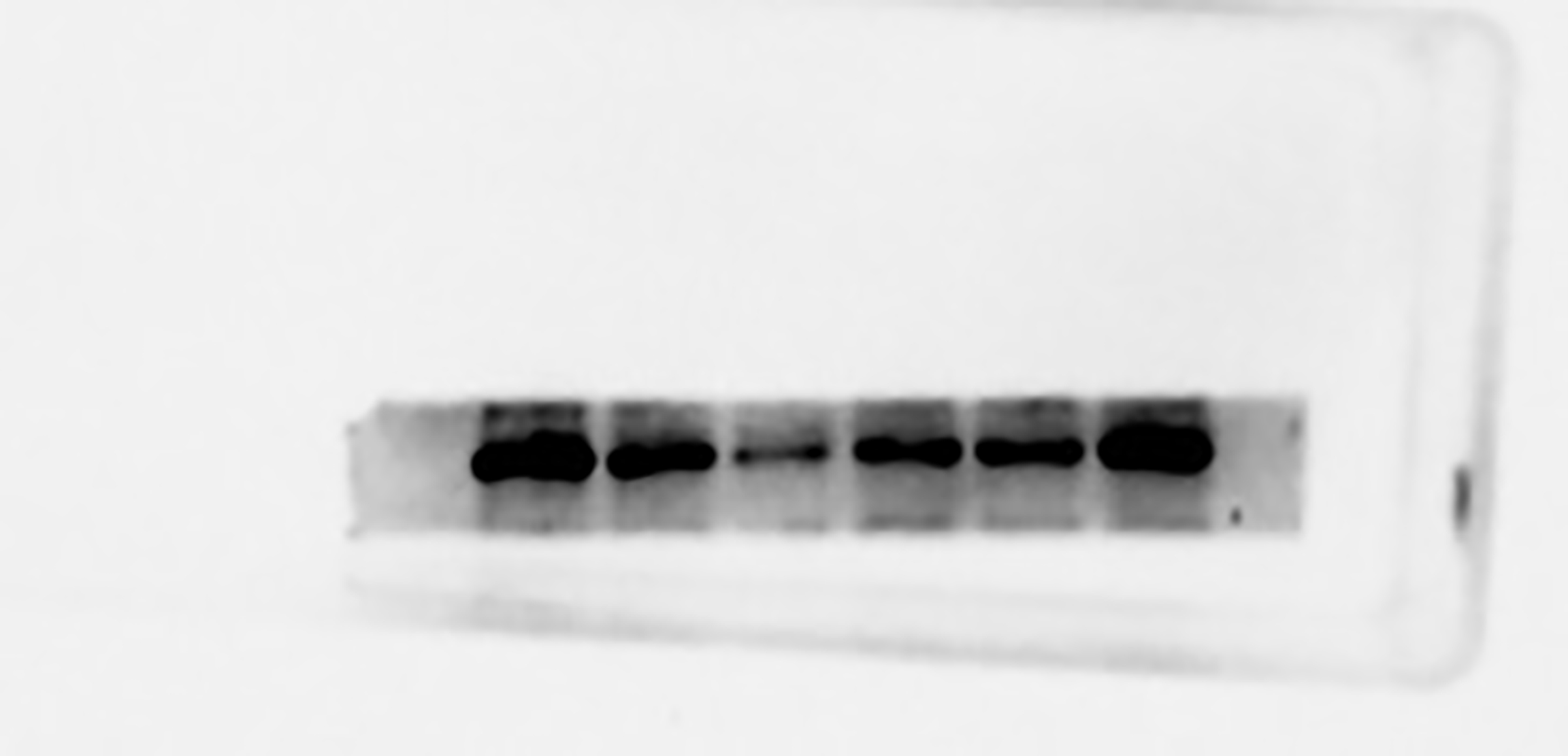

Supplement: Source data 1. [file elife-72266-data1.zip › Source data 1-original files of gels or blots/Figure 7/Figure 7 G ACTIN.jpg]

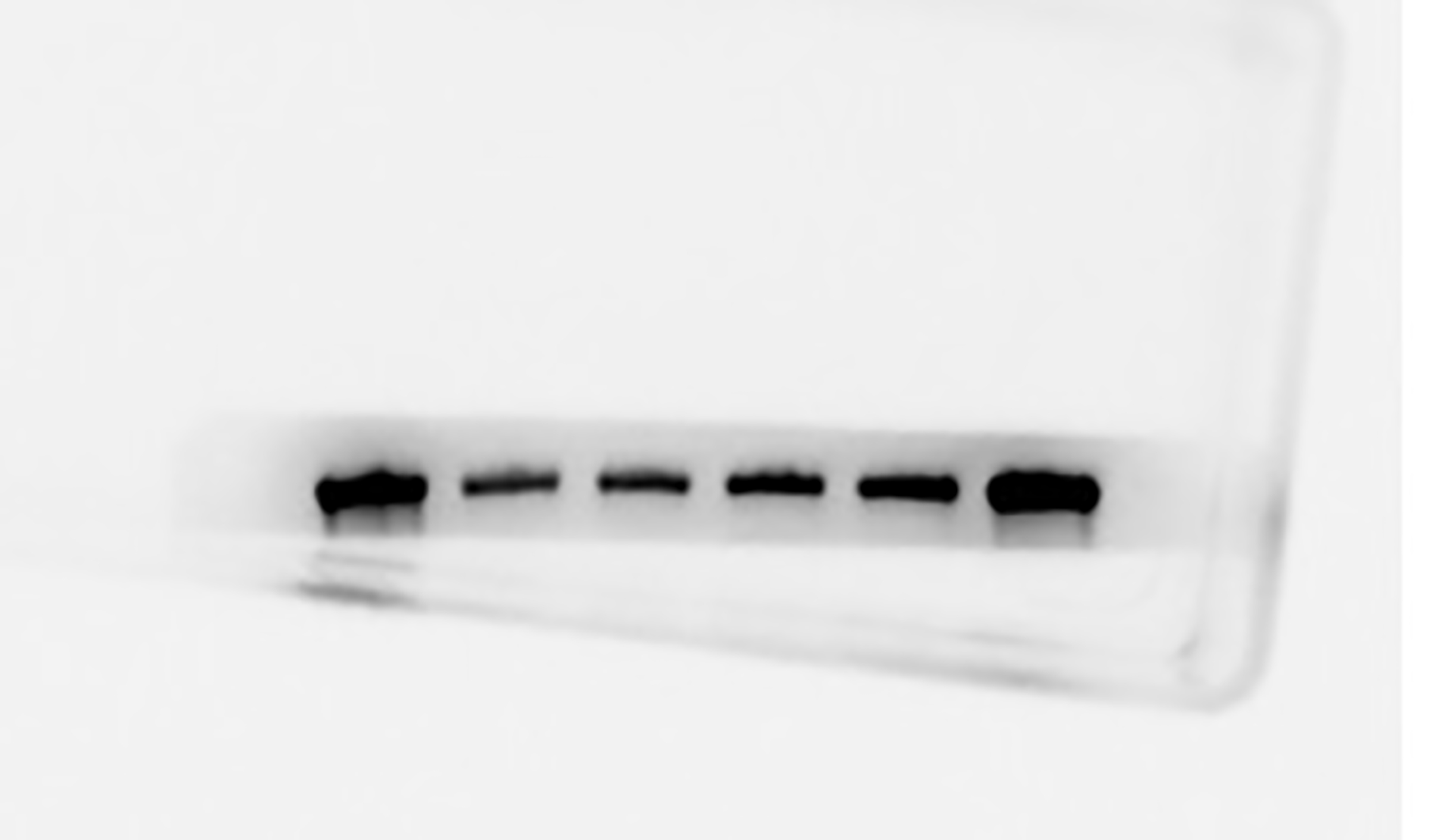

Supplement: Source data 1. [file elife-72266-data1.zip › Source data 1-original files of gels or blots/Figure 7/Figure 7 G PKA.jpg]

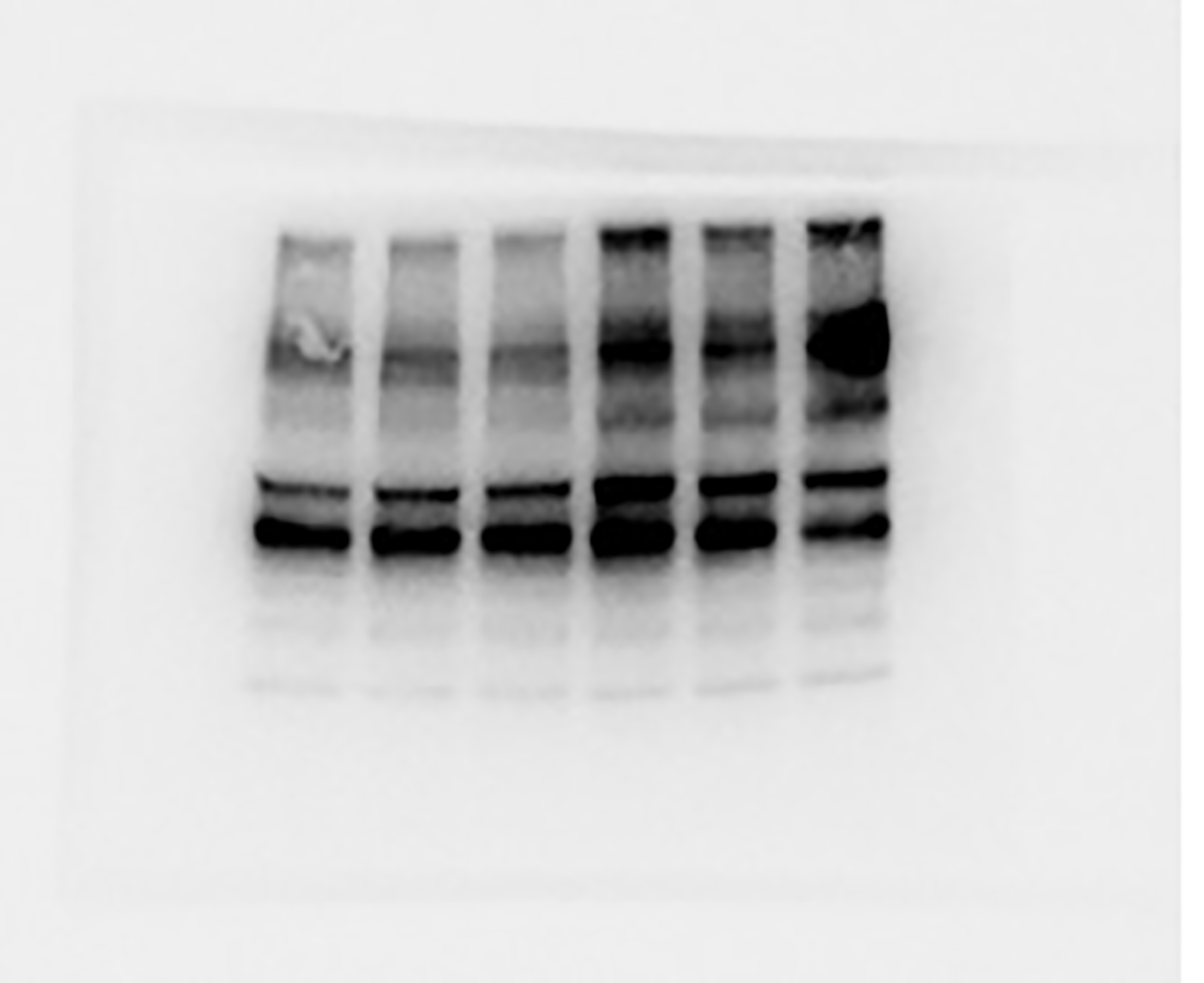

Supplement: Source data 1. [file elife-72266-data1.zip › Source data 1-original files of gels or blots/Figure 7/Figure 7 G pPKA.jpg]

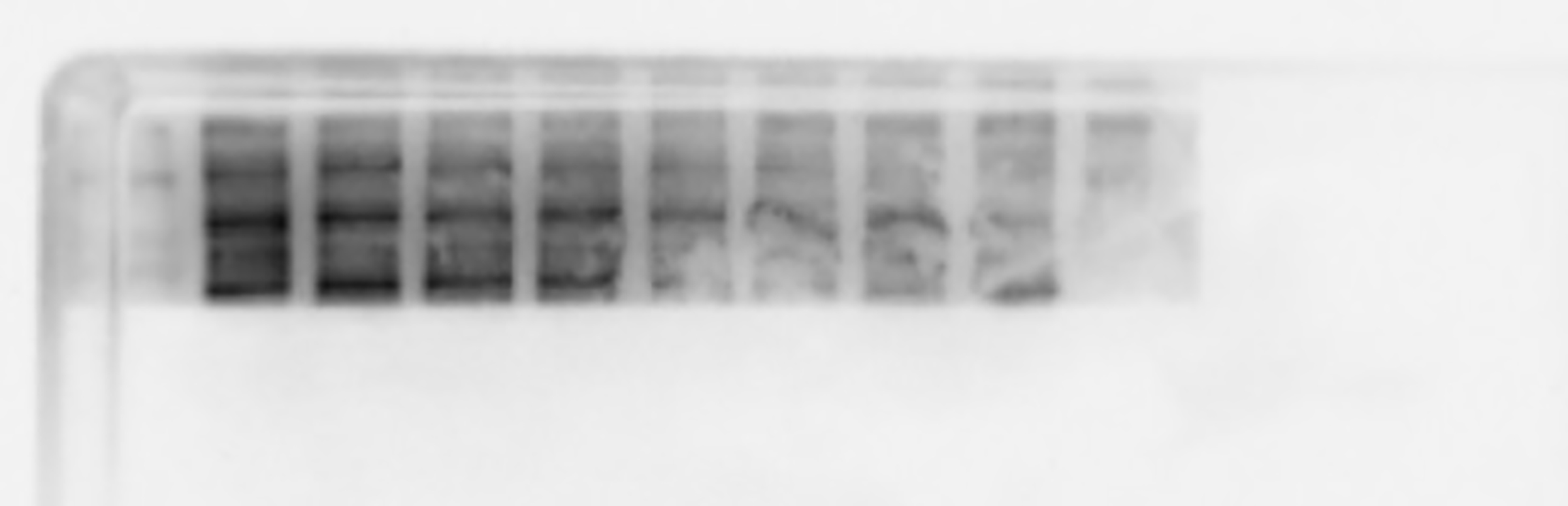

Supplement: Source data 1. [file elife-72266-data1.zip › Source data 1-original files of gels or blots/Figure 7/Figure 7 I PGC-1a.jpg]

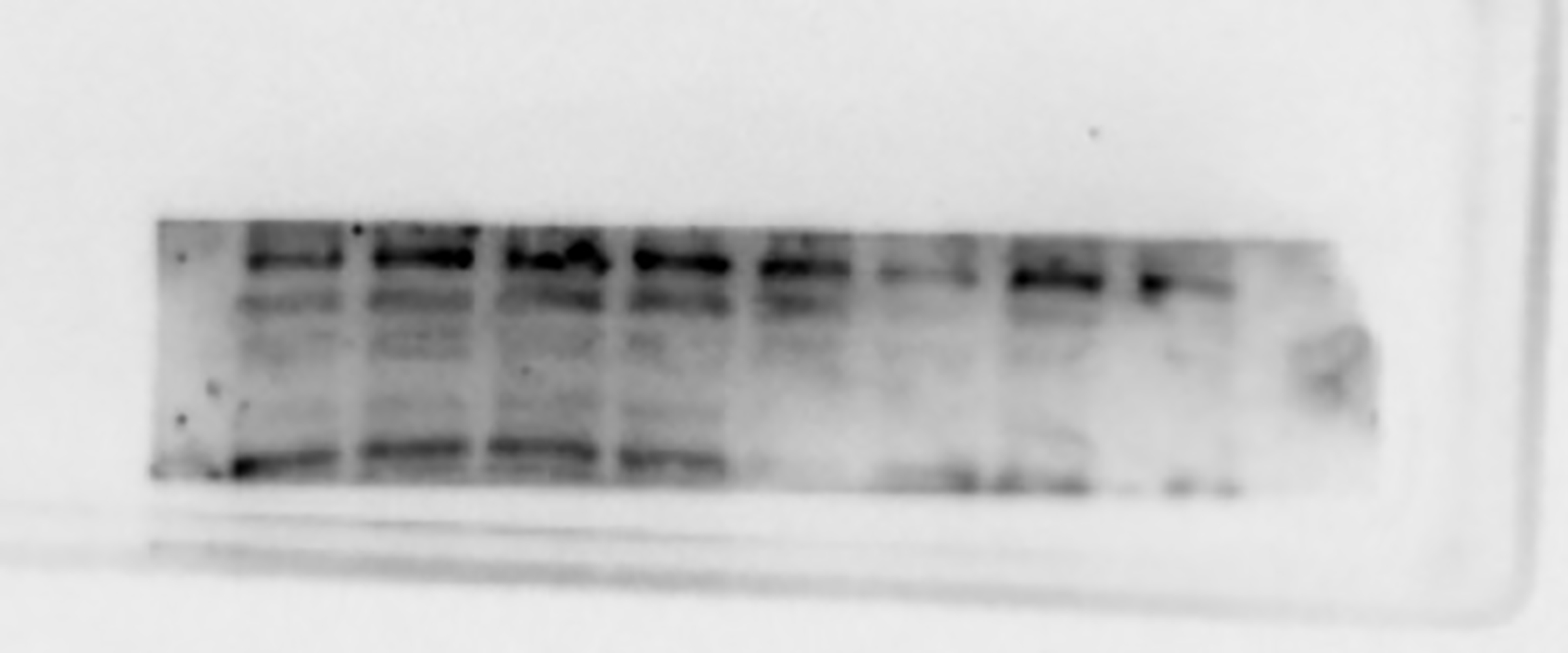

Supplement: Source data 1. [file elife-72266-data1.zip › Source data 1-original files of gels or blots/Figure 7/Figure 7 I UCP1.jpg]

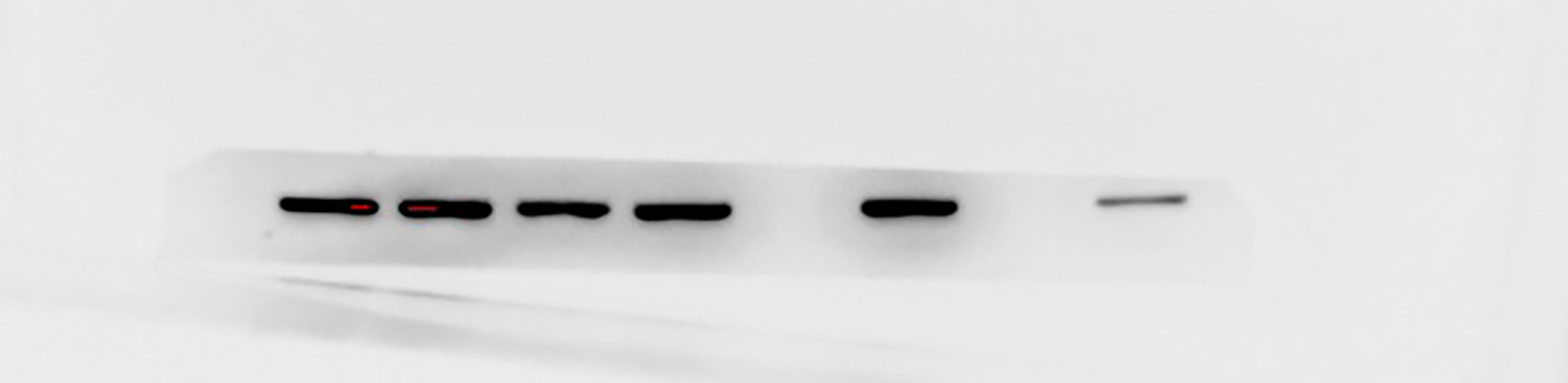

Supplement: Source data 1. [file elife-72266-data1.zip › Source data 1-original files of gels or blots/Figure 7-figure supplement 2/Figure 7-supple 2-A AKT.jpg]

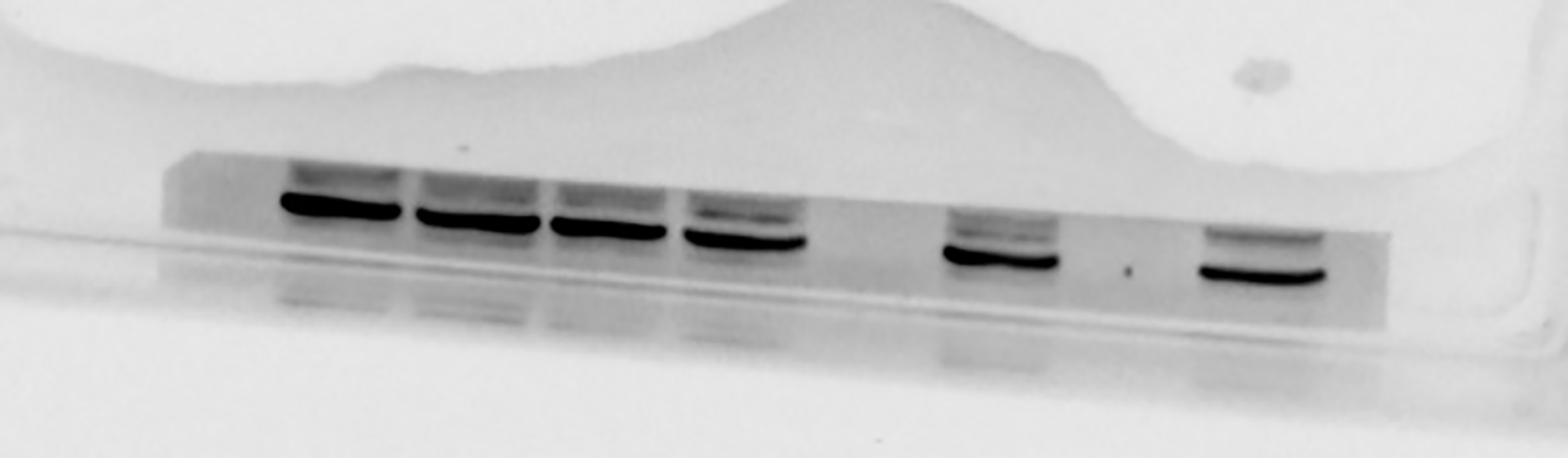

Supplement: Source data 1. [file elife-72266-data1.zip › Source data 1-original files of gels or blots/Figure 7-figure supplement 2/Figure 7-supple 2-A FOXO1.jpg]

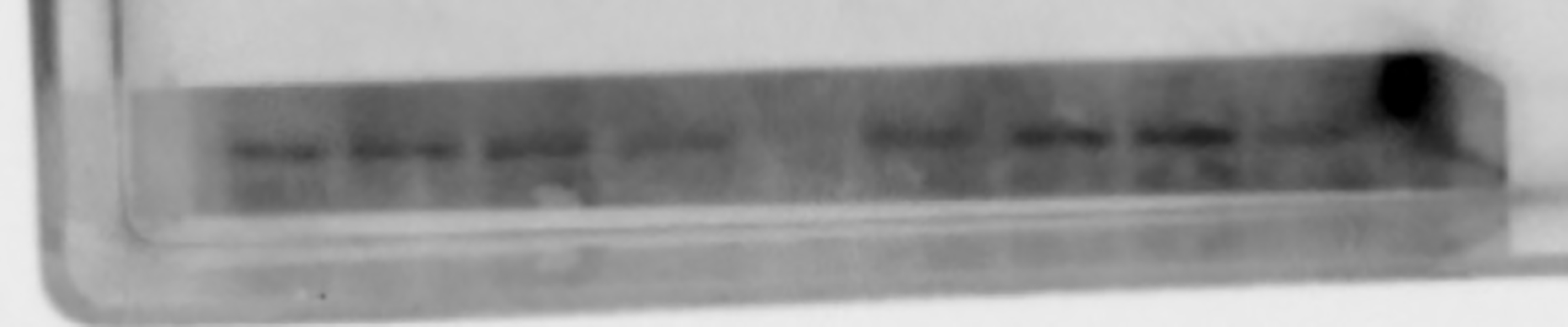

Supplement: Source data 1. [file elife-72266-data1.zip › Source data 1-original files of gels or blots/Figure 7-figure supplement 2/Figure 7-supple 2-A UCP1.jpg]

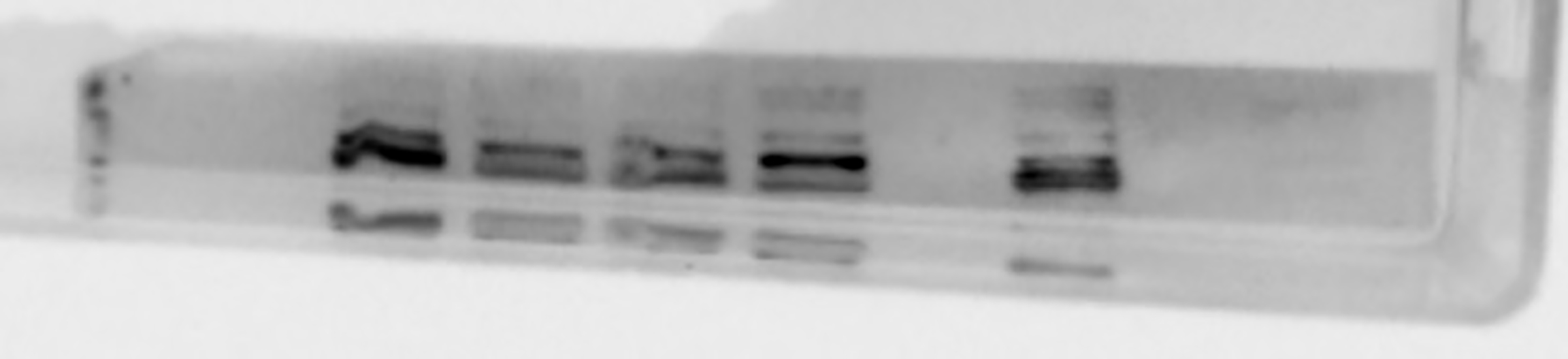

Supplement: Source data 1. [file elife-72266-data1.zip › Source data 1-original files of gels or blots/Figure 7-figure supplement 2/Figure 7-supple 2-A pAKT308.jpg]

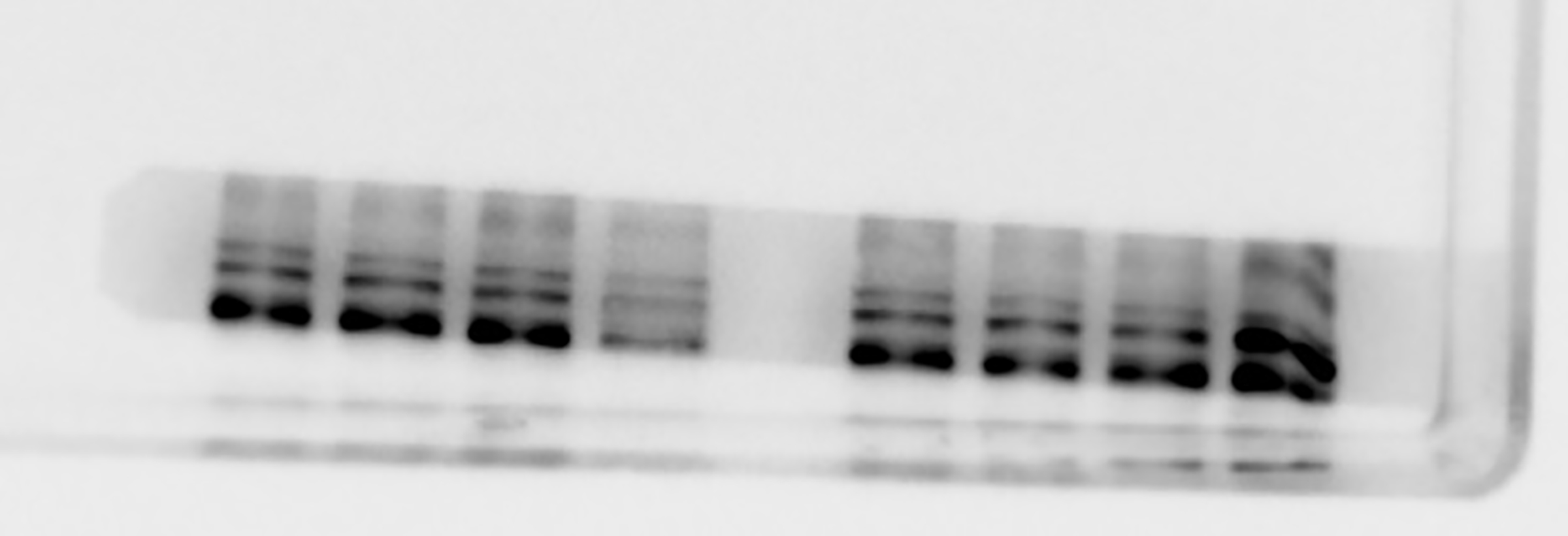

Supplement: Source data 1. [file elife-72266-data1.zip › Source data 1-original files of gels or blots/Figure 7-figure supplement 2/Figure 7-supple 2-A pFOXO1.jpg]

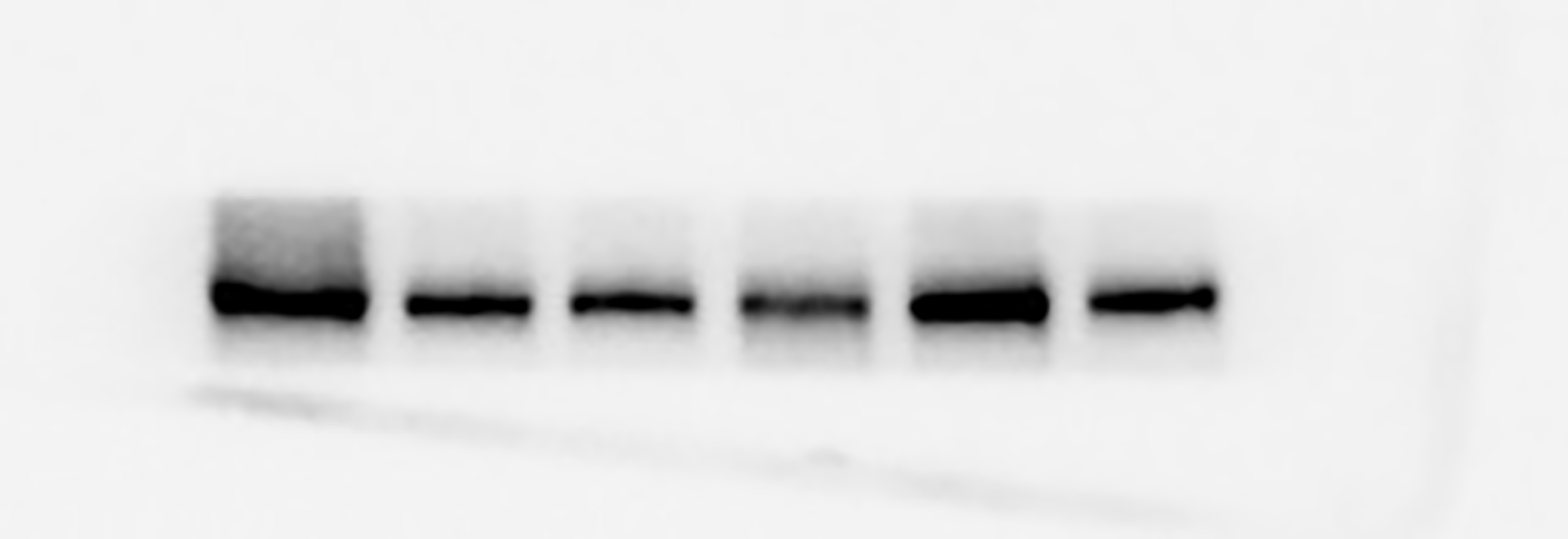

Supplement: Source data 1. [file elife-72266-data1.zip › Source data 1-original files of gels or blots/Figure 7-figure supplement 2/Figure 7-supple 2-D ACTIN.jpg]

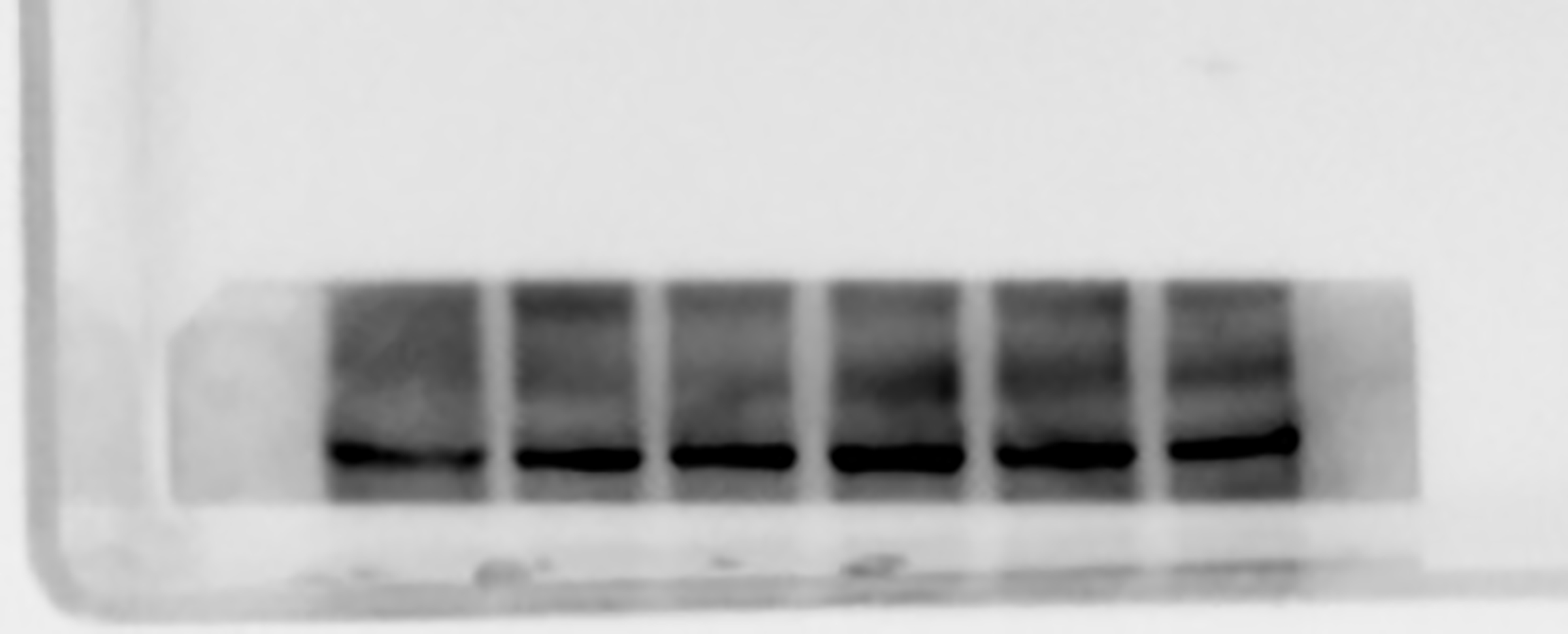

Supplement: Source data 1. [file elife-72266-data1.zip › Source data 1-original files of gels or blots/Figure 7-figure supplement 2/Figure 7-supple 2-D PKA.jpg]

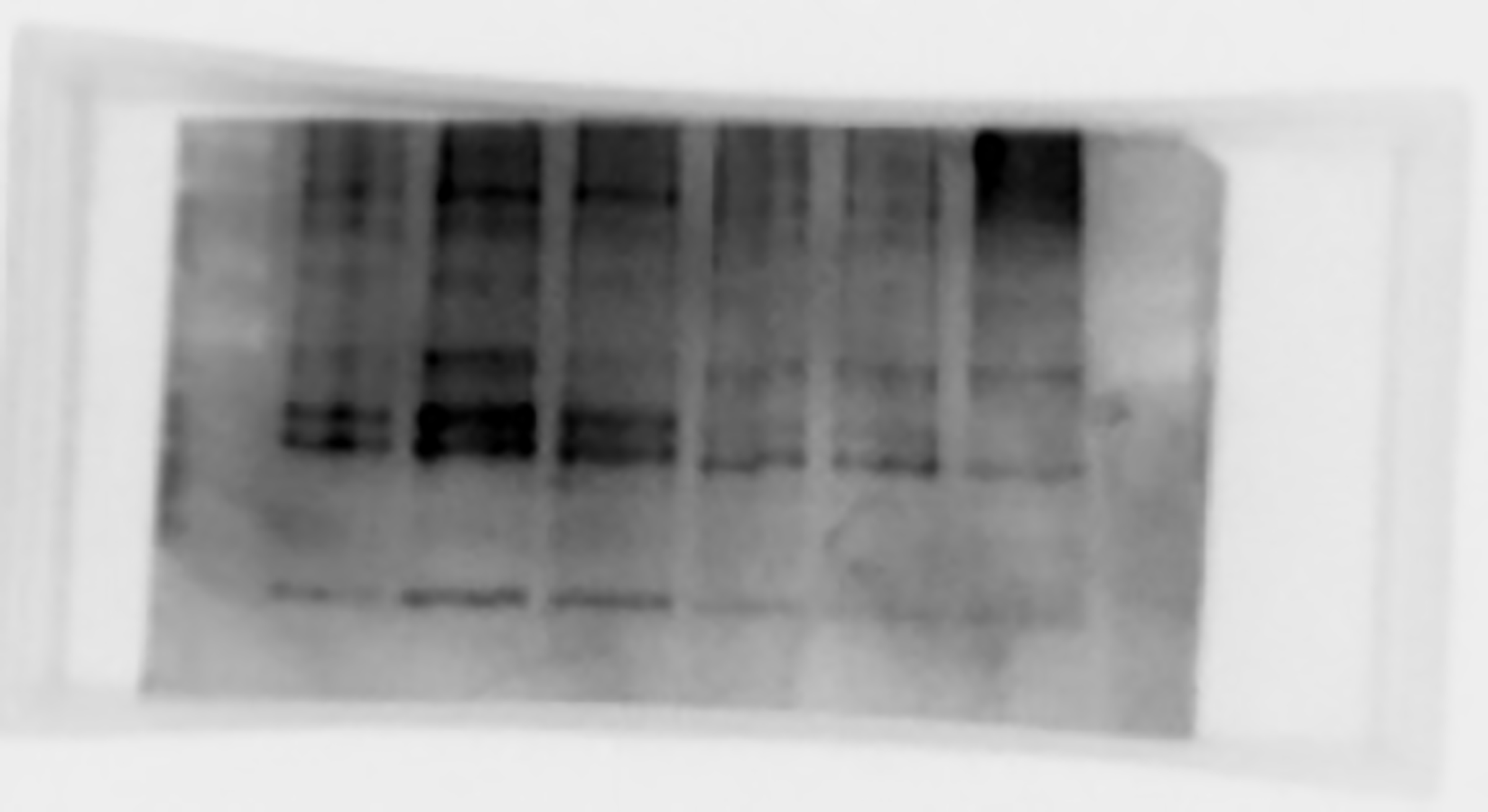

Supplement: Source data 1. [file elife-72266-data1.zip › Source data 1-original files of gels or blots/Figure 7-figure supplement 2/Figure 7-supple 2-D pPKA.jpg]

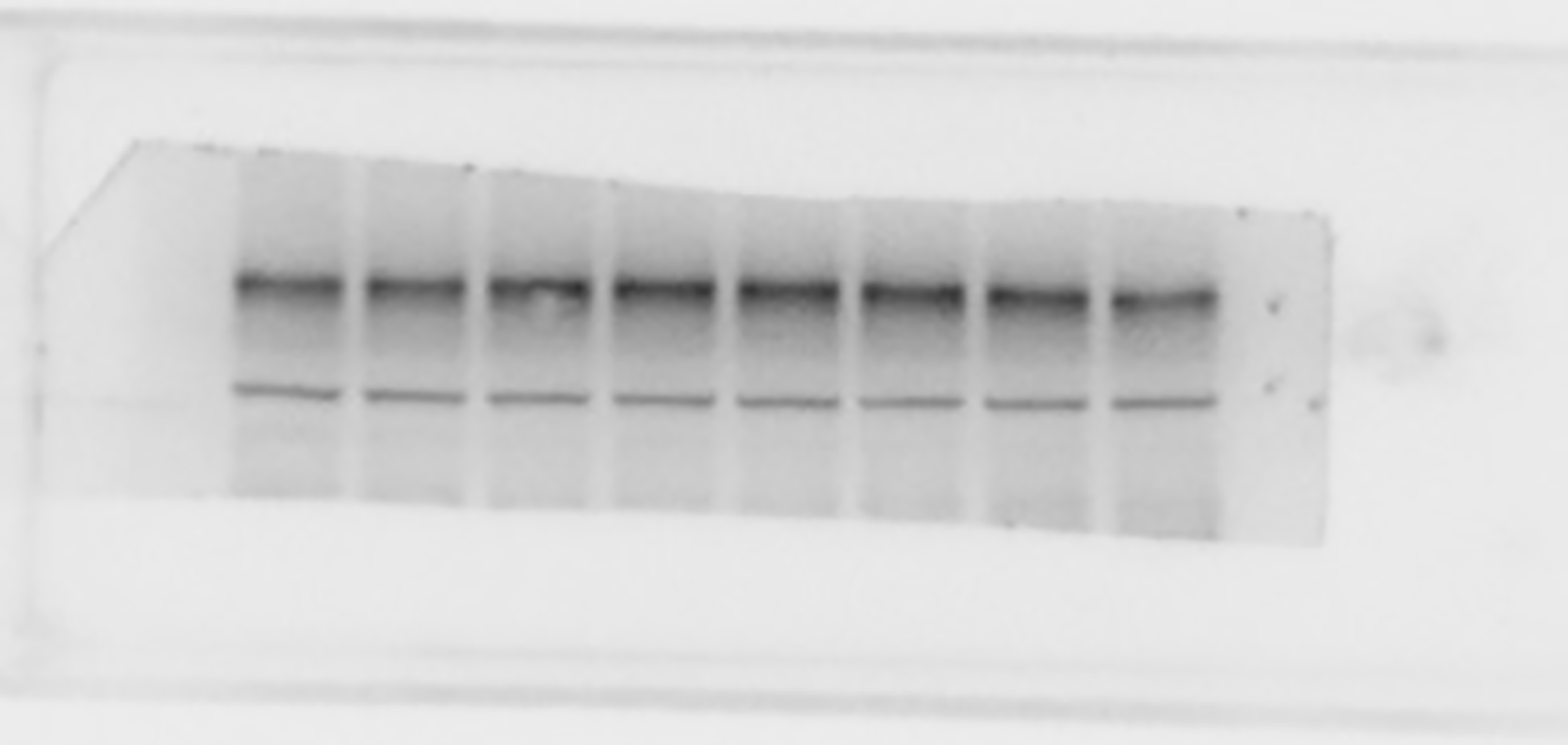

Supplement: Source data 1. [file elife-72266-data1.zip › Source data 1-original files of gels or blots/Figure 7-figure supplement 2/Figure 7-supple 2-E PKA.jpg]

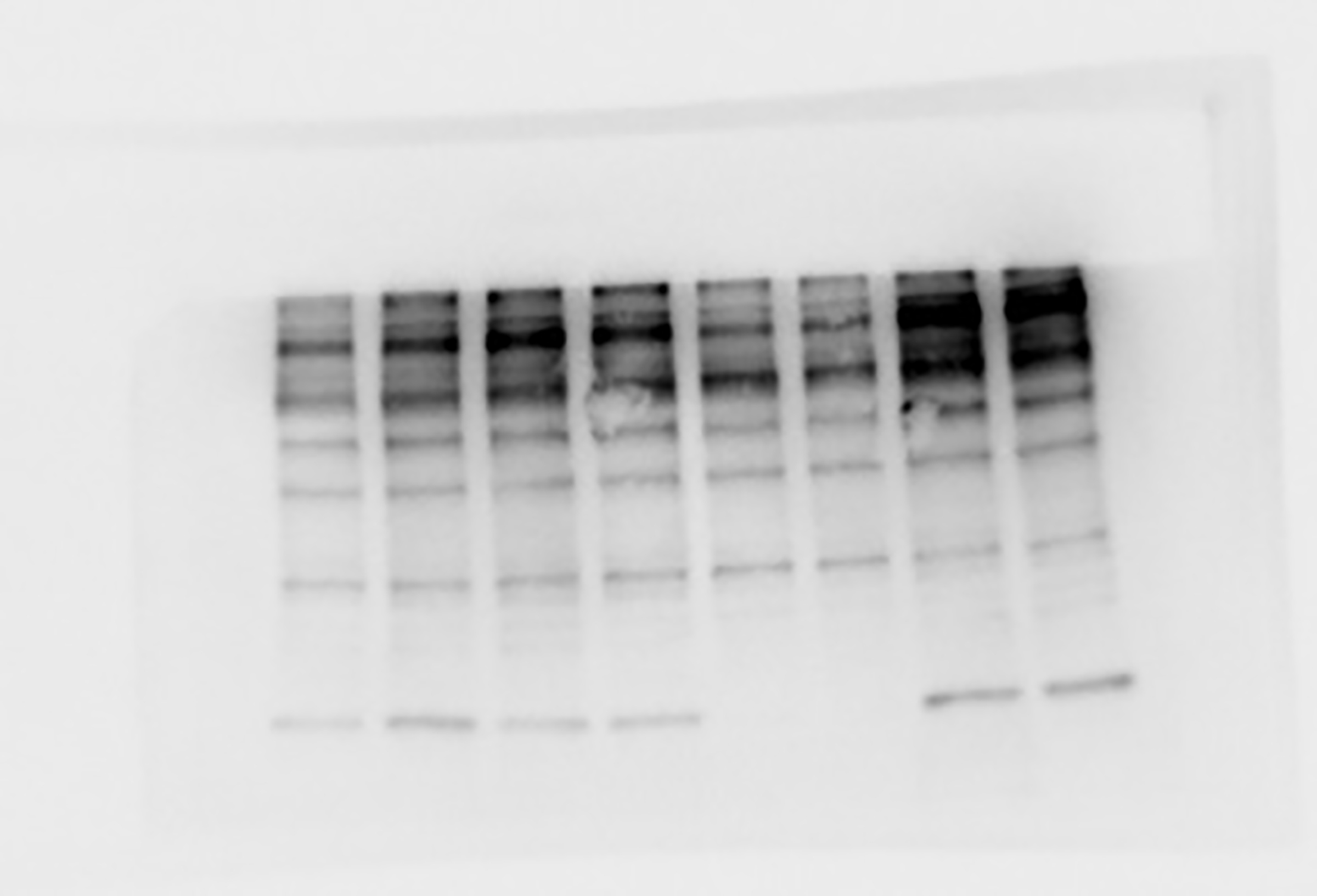

Supplement: Source data 1. [file elife-72266-data1.zip › Source data 1-original files of gels or blots/Figure 7-figure supplement 2/Figure 7-supple 2-E pPKA.jpg]

## Slide 1
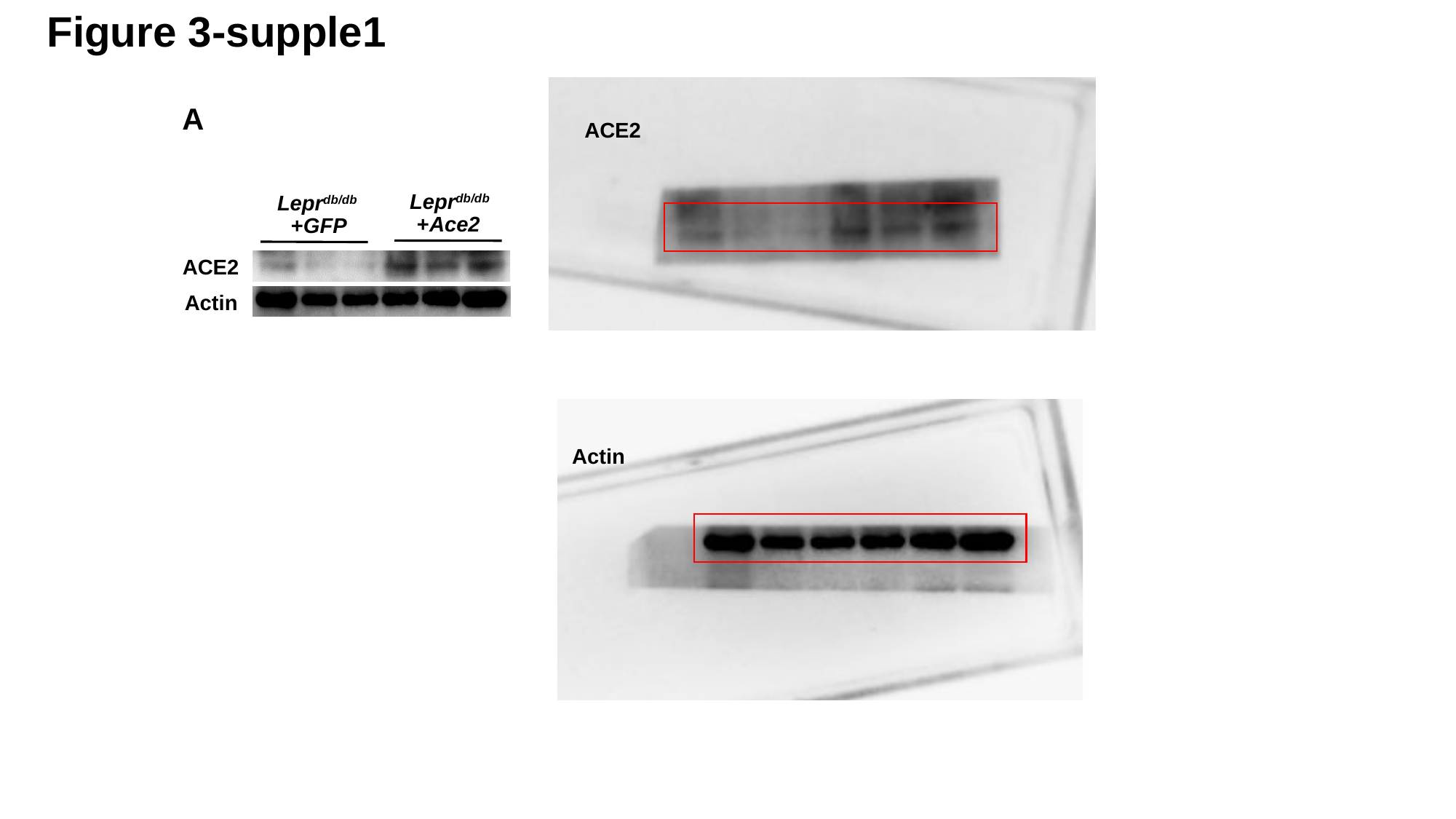

Figure 3-supple1
A
ACE2
Leprdb/db
+Ace2
Leprdb/db
+GFP
ACE2
Actin
Actin

Supplement: Source data 2. [file elife-72266-data2.zip › Source data 2--PowerPoint of gels or blots/Figure 3-figure supplement 1- Ace2 enhance BAT activity and whole-body energy m-source data 2.pptx]

## Slide 1
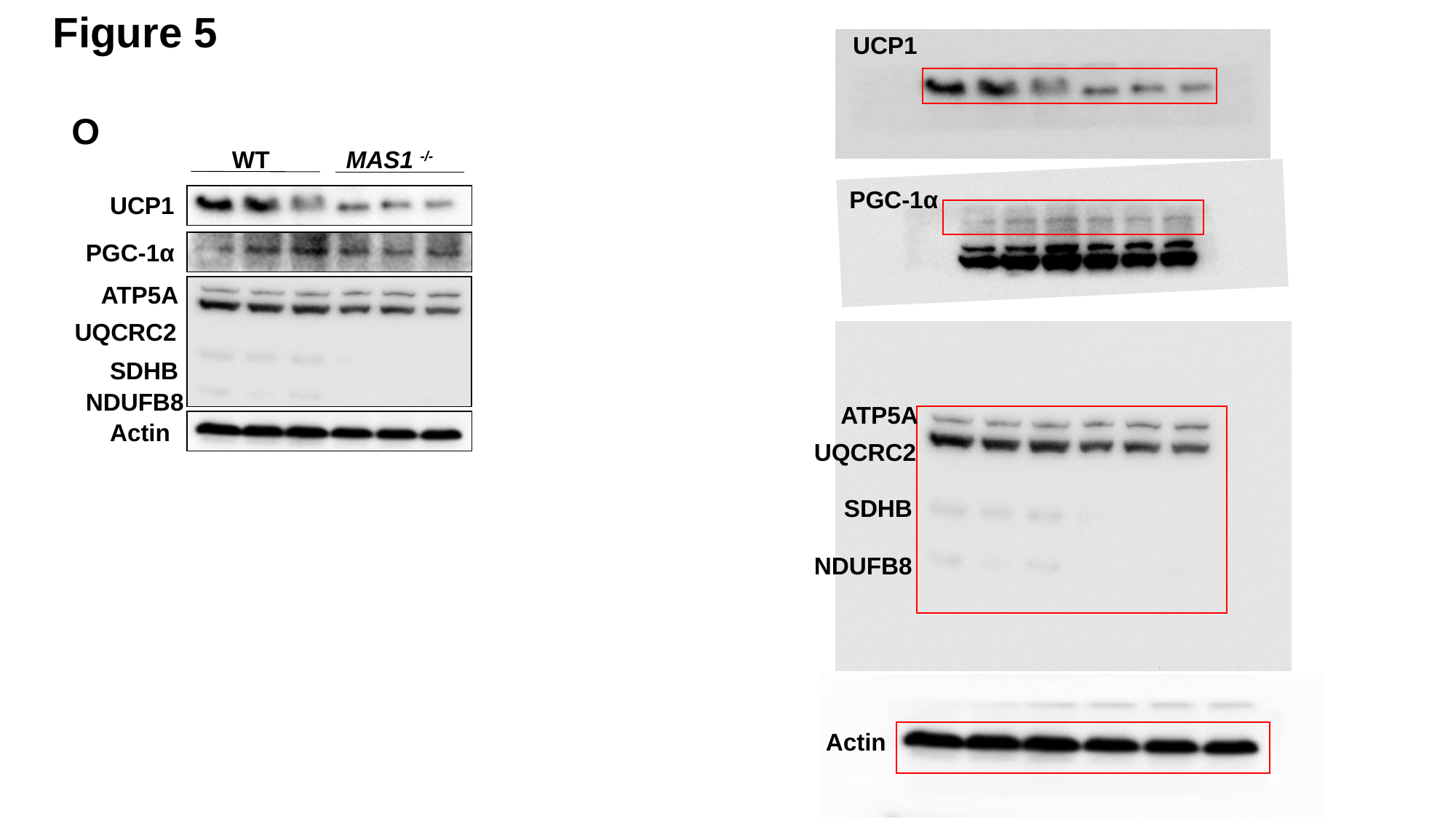

Figure 5
UCP1
O
WT
MAS1 -/-
UCP1
PGC-1α
ATP5A
UQCRC2
SDHB
Actin
PGC-1α
NDUFB8
ATP5A
UQCRC2
SDHB
NDUFB8
Actin

Supplement: Source data 2. [file elife-72266-data2.zip › Source data 2--PowerPoint of gels or blots/Figure 5-Ablation of Mas1 impairs thermogenesis, BAT activity-source data 2.pptx]
