# Supplementary material for: ACE2 pathway regulates thermogenesis and energy metabolism
Source: eLife. 2022 Jan 11;11:e72266. doi: 10.7554/eLife.72266 (PMC8776250; doi:10.7554/eLife.72266)
Supplement: Source data 2. [file elife-72266-data2.zip › Source data 2--PowerPoint of gels or blots/Figure 1-Ace2 pathway is activated by cold exposure-source data 2.pptx]

## Slide 1
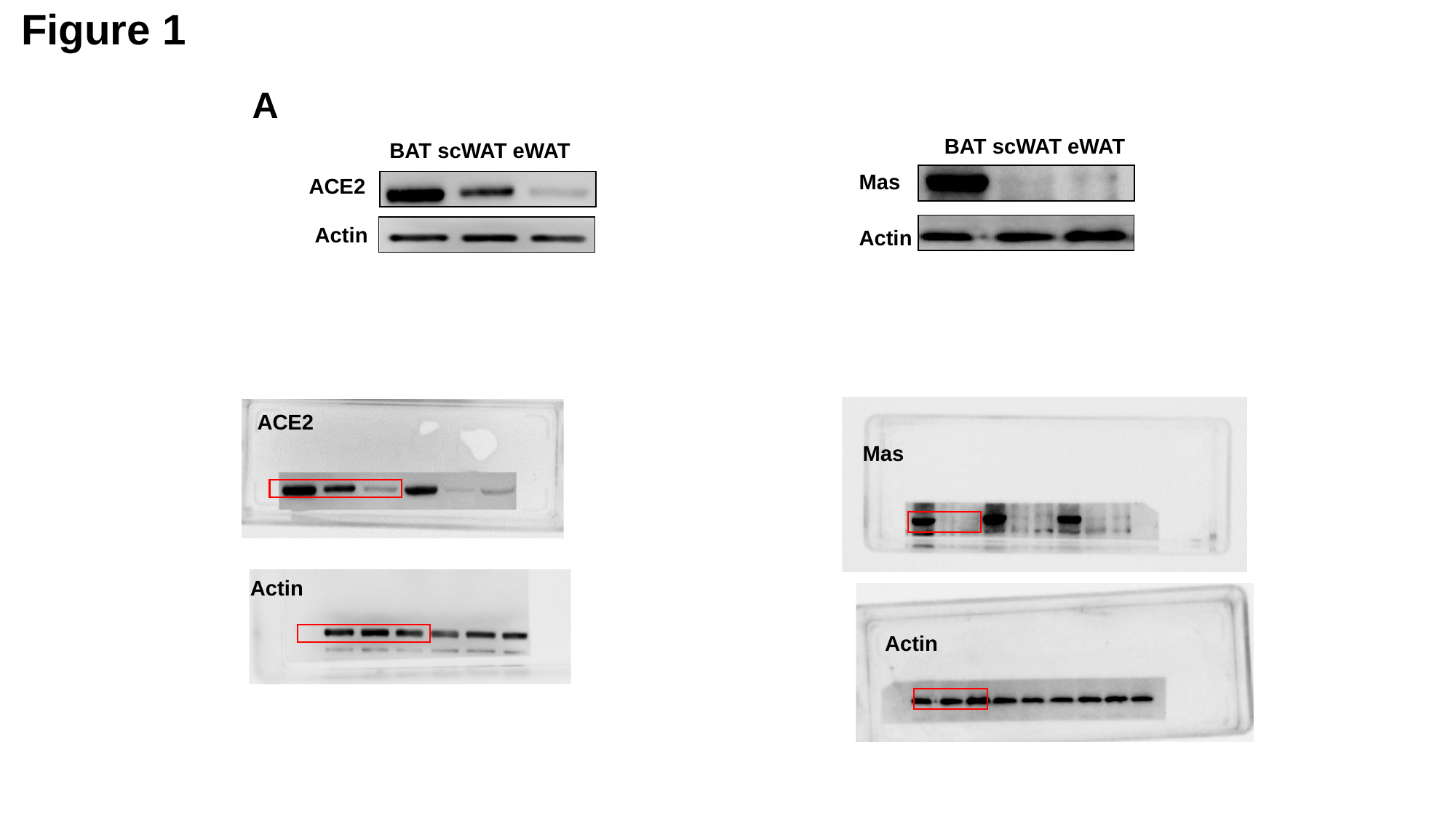

Figure 1
A
 BAT scWAT eWAT
Mas
Actin
 BAT scWAT eWAT
ACE2
Actin
Mas
Actin
ACE2
Actin

## Slide 2
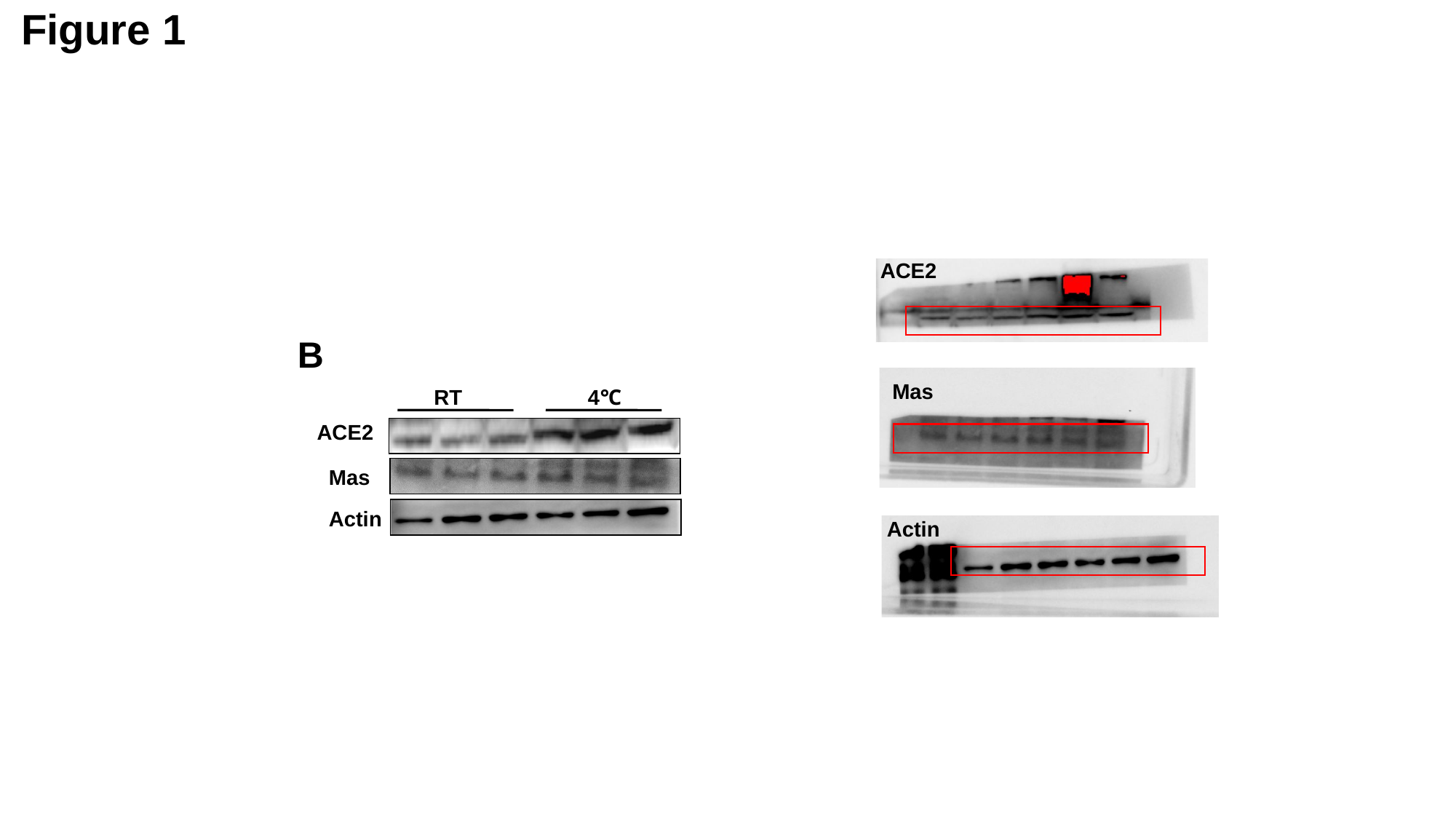

Figure 1
ACE2
Mas
Actin
B
RT 4℃
ACE2
Mas
Actin
