# Supplementary material for: ACE2 pathway regulates thermogenesis and energy metabolism
Source: eLife. 2022 Jan 11;11:e72266. doi: 10.7554/eLife.72266 (PMC8776250; doi:10.7554/eLife.72266)
Supplement: Source data 2. [file elife-72266-data2.zip › Source data 2--PowerPoint of gels or blots/Figure 2-Ace2 deficiency impairs thermogenesis, BAT activity-source data 2.pptx]

## Slide 1
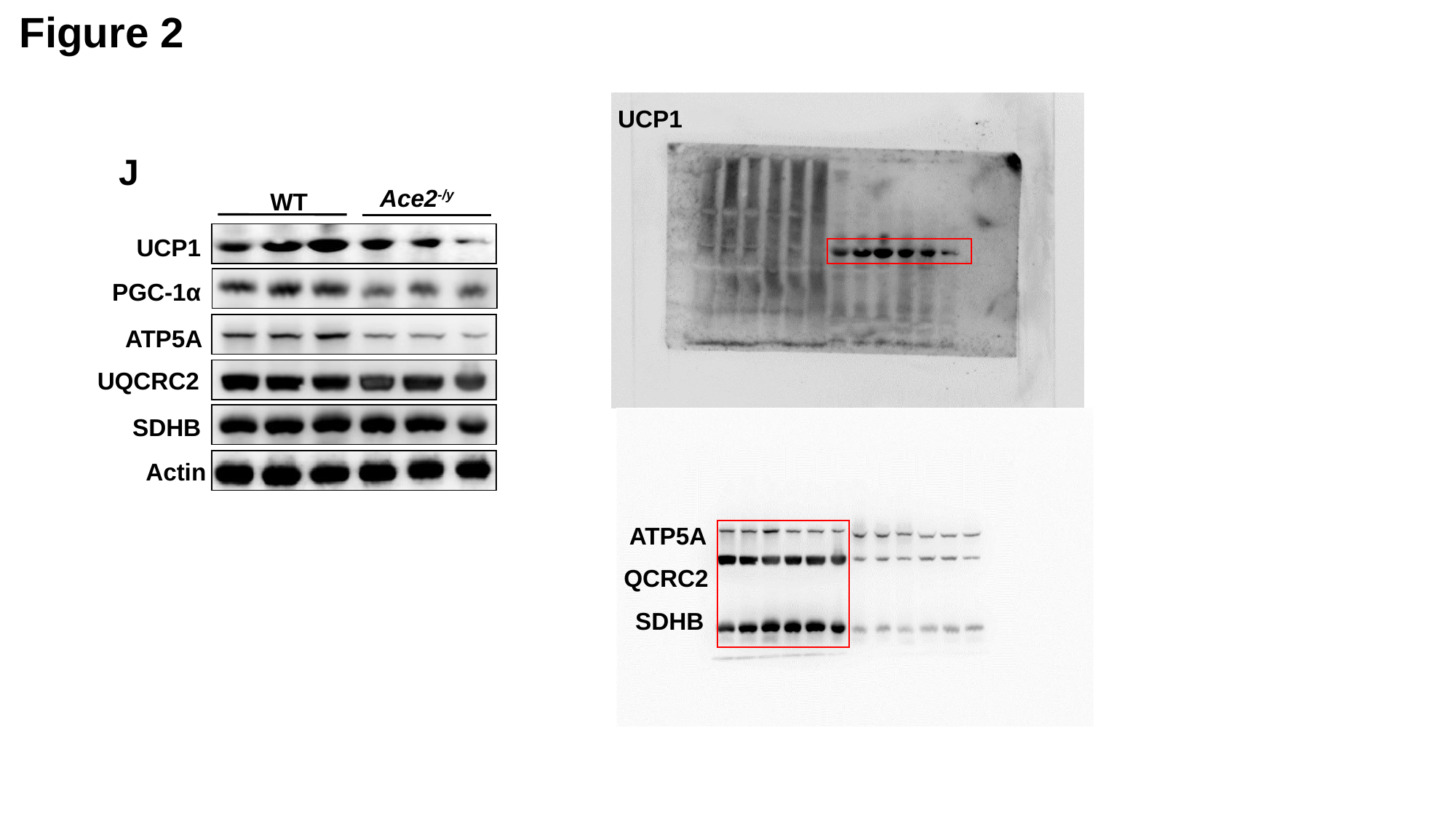

Figure 2
UCP1
ATP5A
QCRC2
SDHB
J
Ace2-/y
WT
UCP1
PGC-1α
ATP5A
UQCRC2
SDHB
Actin

## Slide 2
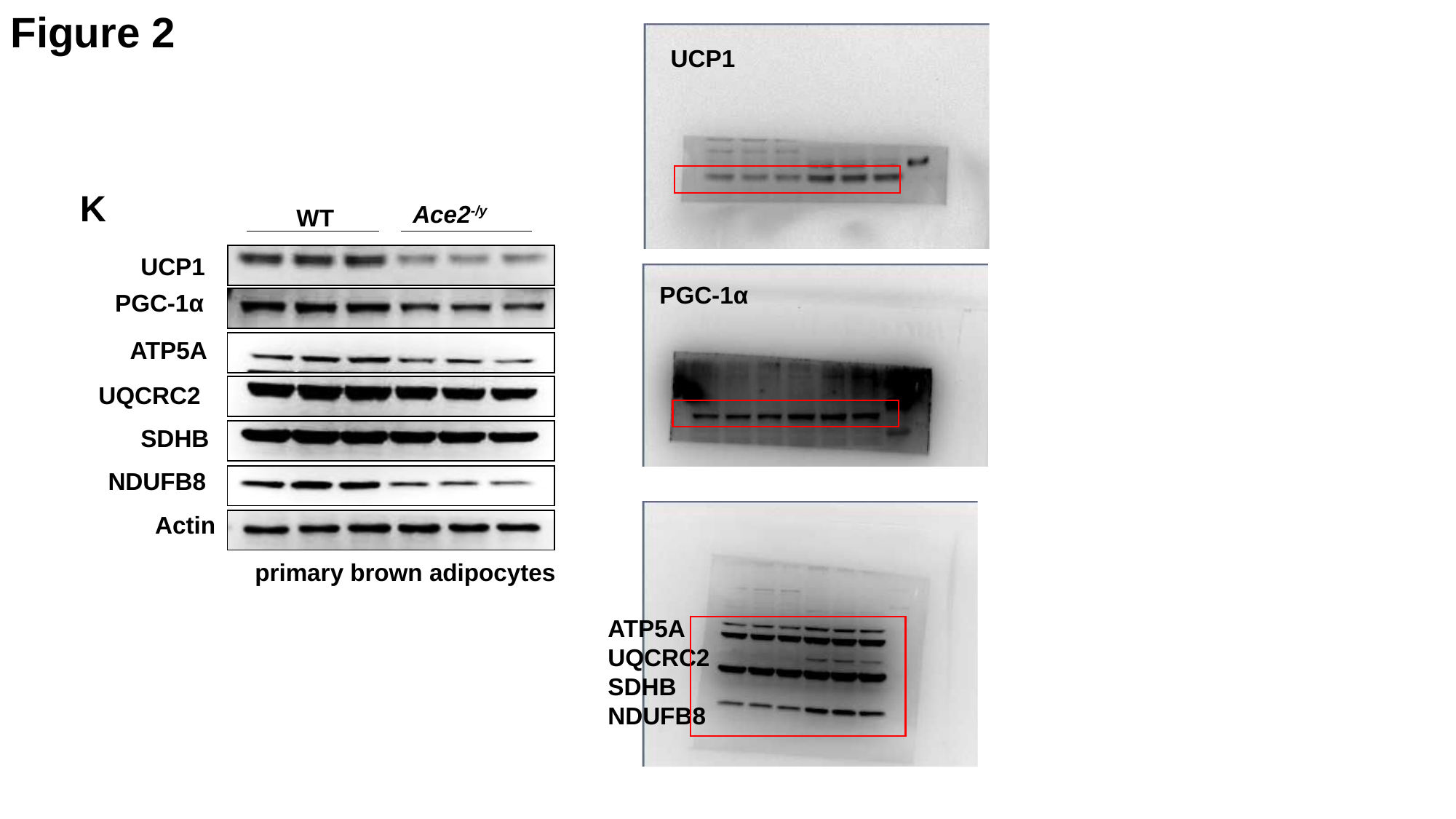

Figure 2
UCP1
PGC-1α
ATP5A UQCRC2 SDHB
NDUFB8
K
Ace2-/y
WT
UCP1
PGC-1α
ATP5A
UQCRC2
SDHB
NDUFB8
Actin
primary brown adipocytes
