# Supplementary material for: ACE2 pathway regulates thermogenesis and energy metabolism
Source: eLife. 2022 Jan 11;11:e72266. doi: 10.7554/eLife.72266 (PMC8776250; doi:10.7554/eLife.72266)
Supplement: Source data 2. [file elife-72266-data2.zip › Source data 2--PowerPoint of gels or blots/Figure 3-Ace2 enhances thermogenesis, BAT activity, and energy metabolism in db-source data 2.pptx]

## Slide 1
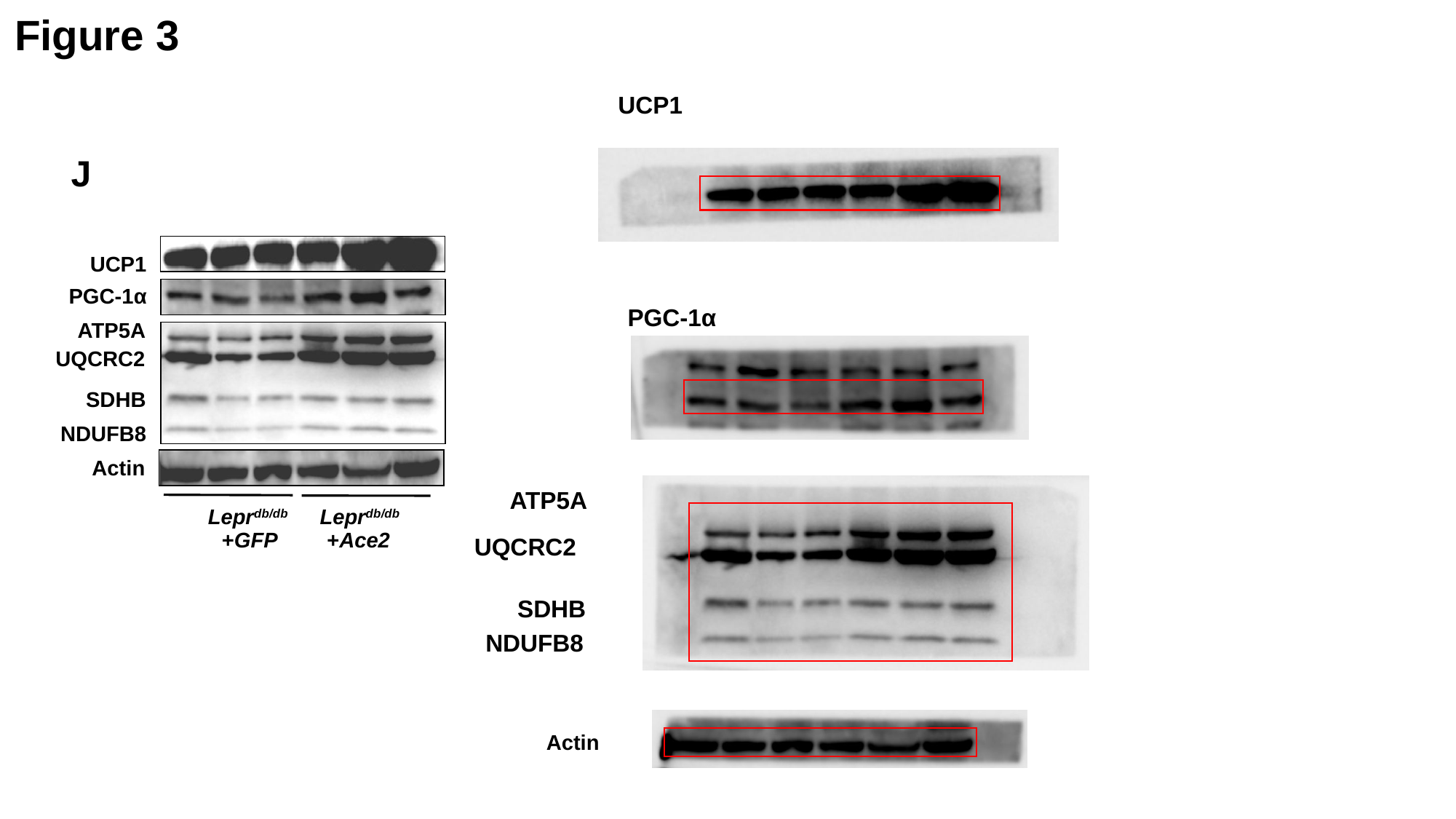

Figure 3
UCP1
PGC-1α
ATP5A
UQCRC2
SDHB
NDUFB8
Actin
J
UCP1
PGC-1α
ATP5A
UQCRC2
SDHB
NDUFB8
Actin
Leprdb/db
+GFP
Leprdb/db
+Ace2
