# Supplementary material for: ACE2 pathway regulates thermogenesis and energy metabolism
Source: eLife. 2022 Jan 11;11:e72266. doi: 10.7554/eLife.72266 (PMC8776250; doi:10.7554/eLife.72266)
Supplement: Source data 2. [file elife-72266-data2.zip › Source data 2--PowerPoint of gels or blots/Figure 4-Ang-(1-7) promotes thermogenesis, BAT activity, and energy metabolism-source data 2.pptx]

## Slide 1
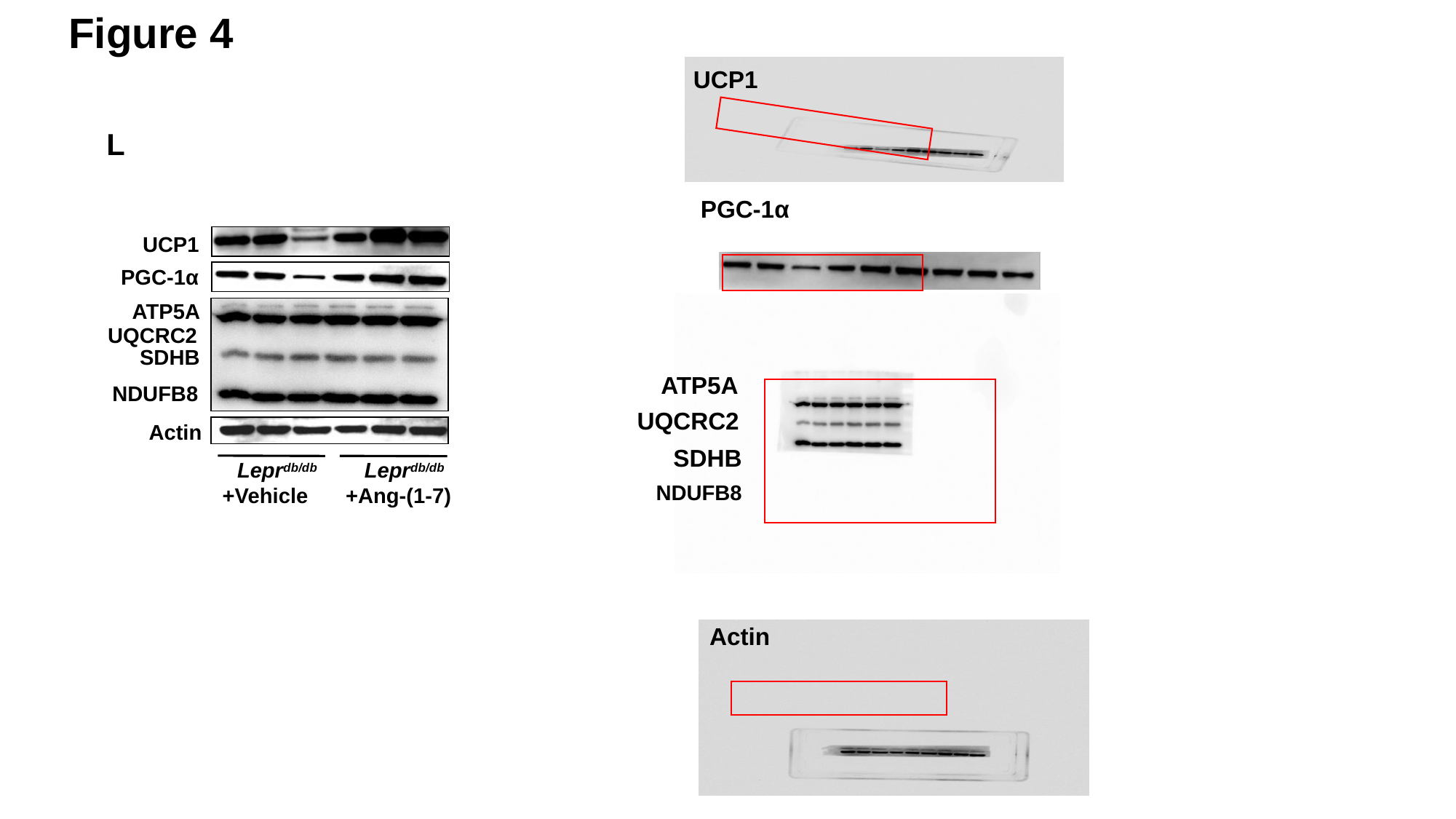

Figure 4
UCP1
L
PGC-1α
UCP1
PGC-1α
ATP5A
UQCRC2
SDHB
NDUFB8
Actin
 Leprdb/db +Vehicle
 Leprdb/db
+Ang-(1-7)
ATP5A
UQCRC2
SDHB
NDUFB8
Actin

## Slide 2
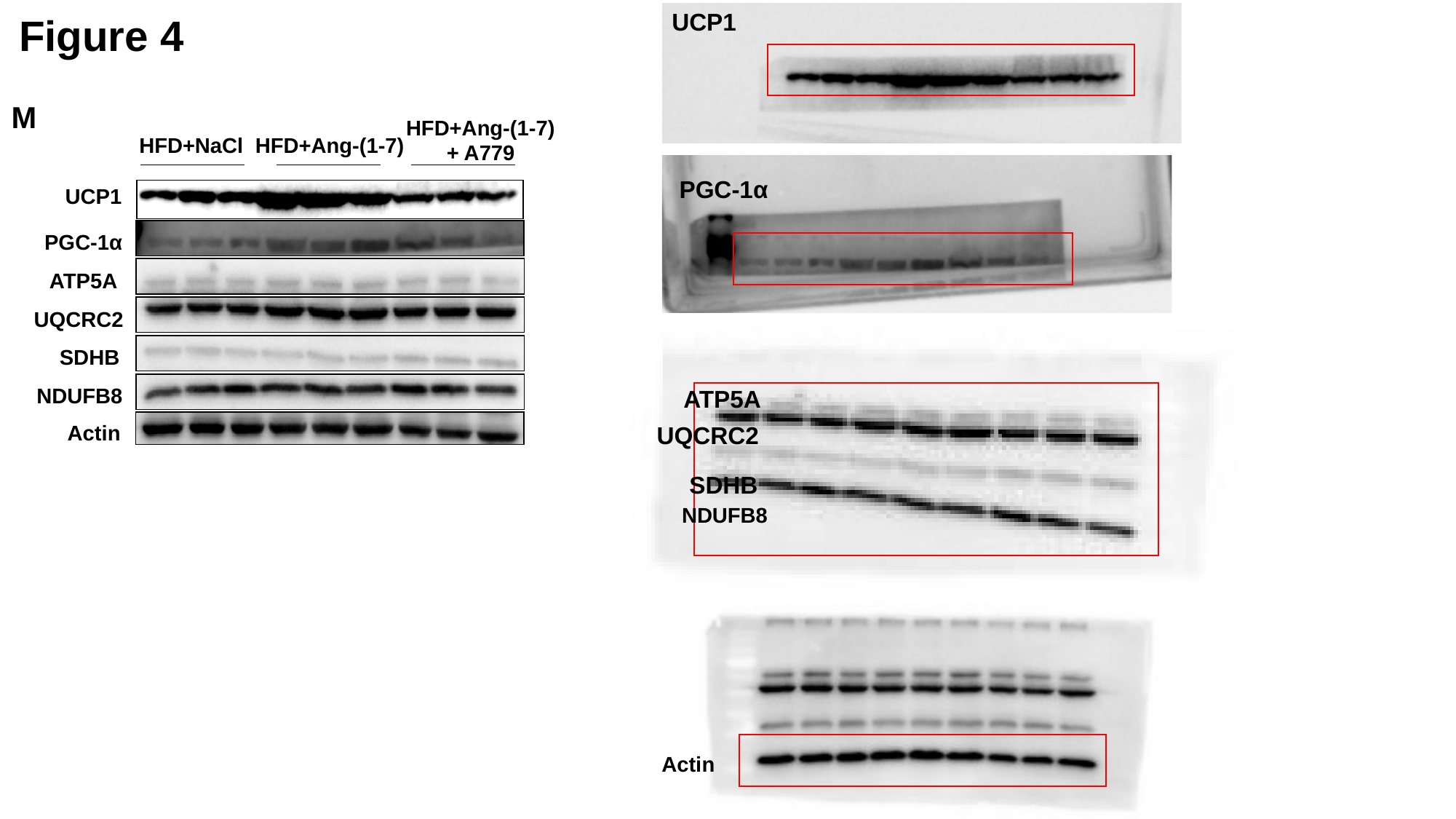

UCP1
Figure 4
M
HFD+Ang-(1-7)
+ A779
 HFD+NaCl HFD+Ang-(1-7)
PGC-1α
UCP1
PGC-1α
ATP5A
UQCRC2
SDHB
NDUFB8
ATP5A
Actin
UQCRC2
SDHB
NDUFB8
Actin
