# Supplementary material for: ACE2 pathway regulates thermogenesis and energy metabolism
Source: eLife. 2022 Jan 11;11:e72266. doi: 10.7554/eLife.72266 (PMC8776250; doi:10.7554/eLife.72266)
Supplement: Source data 2. [file elife-72266-data2.zip › Source data 2--PowerPoint of gels or blots/Figure 7-Ace2 pathway induces a thermogenesis program through the Akt signaling-source data 2.pptx]

## Slide 1
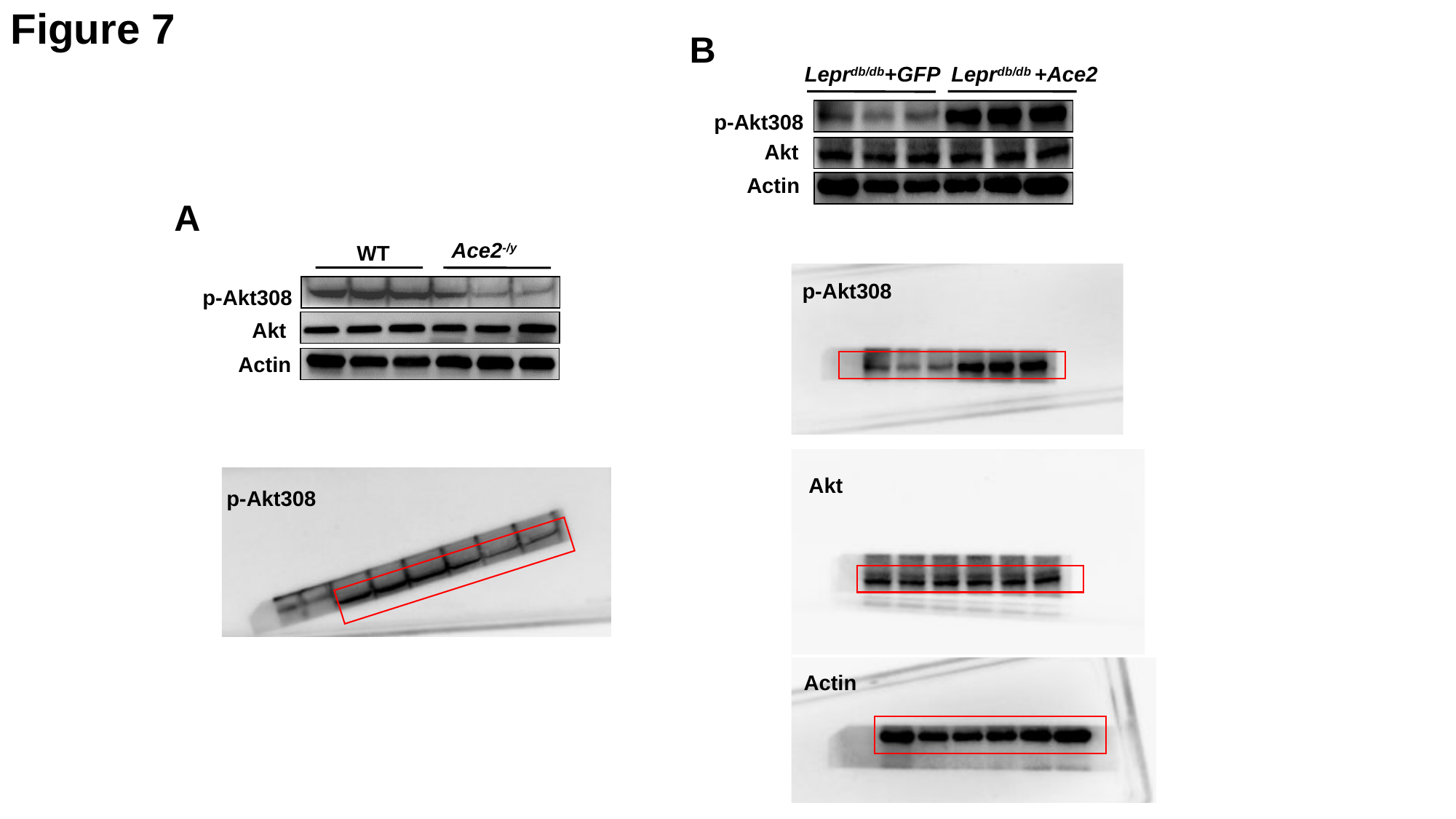

Figure 7
B
Leprdb/db+GFP
Leprdb/db +Ace2
p-Akt308
Akt
Actin
A
WT
Ace2-/y
p-Akt308
Akt
Actin
p-Akt308
Akt
p-Akt308
Actin

## Slide 2
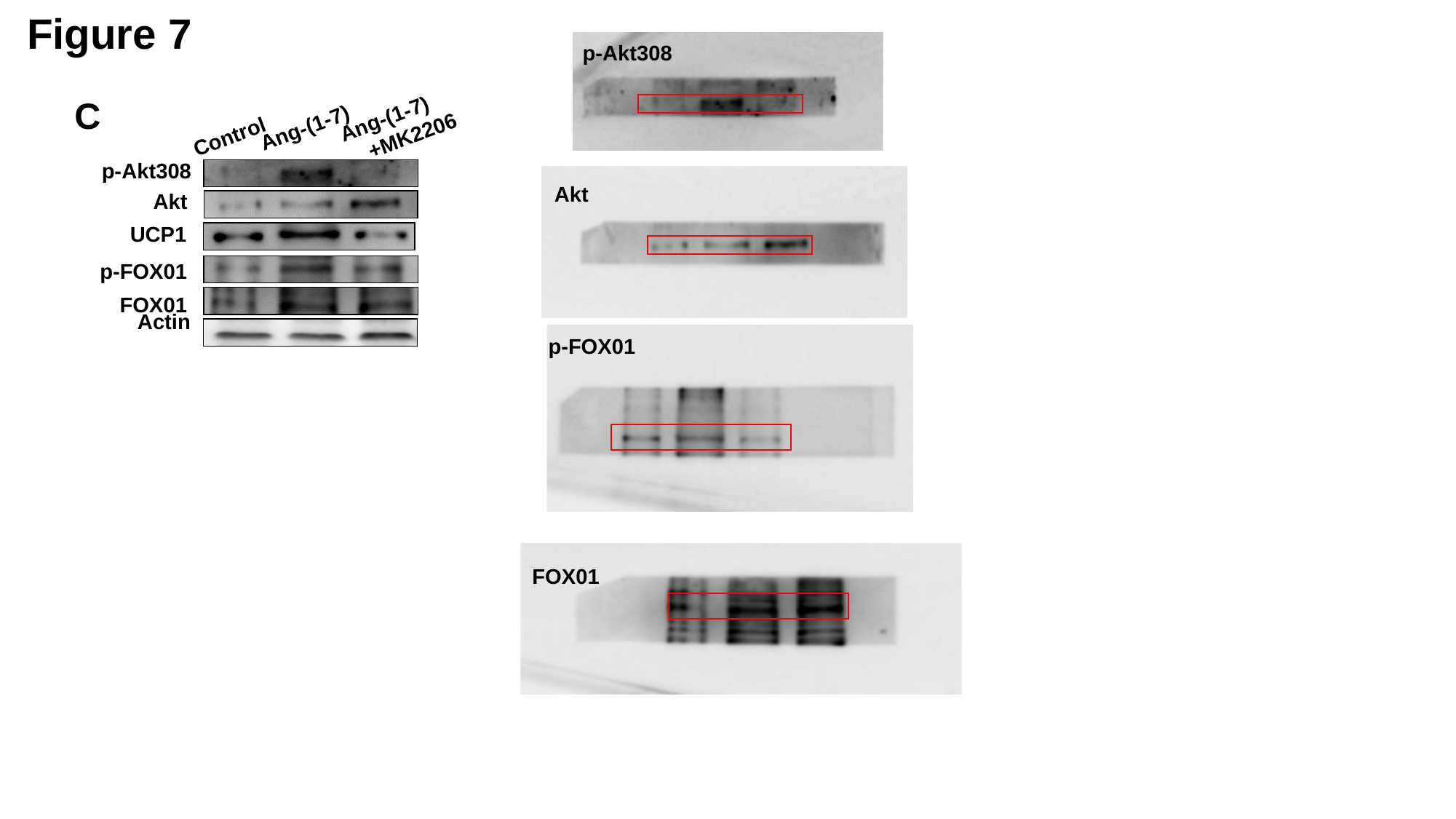

Figure 7
p-Akt308
C
 Ang-(1-7)
+MK2206
Ang-(1-7)
Control
p-Akt308
Akt
Akt
UCP1
p-FOX01
FOX01
Actin
p-FOX01
FOX01

## Slide 3
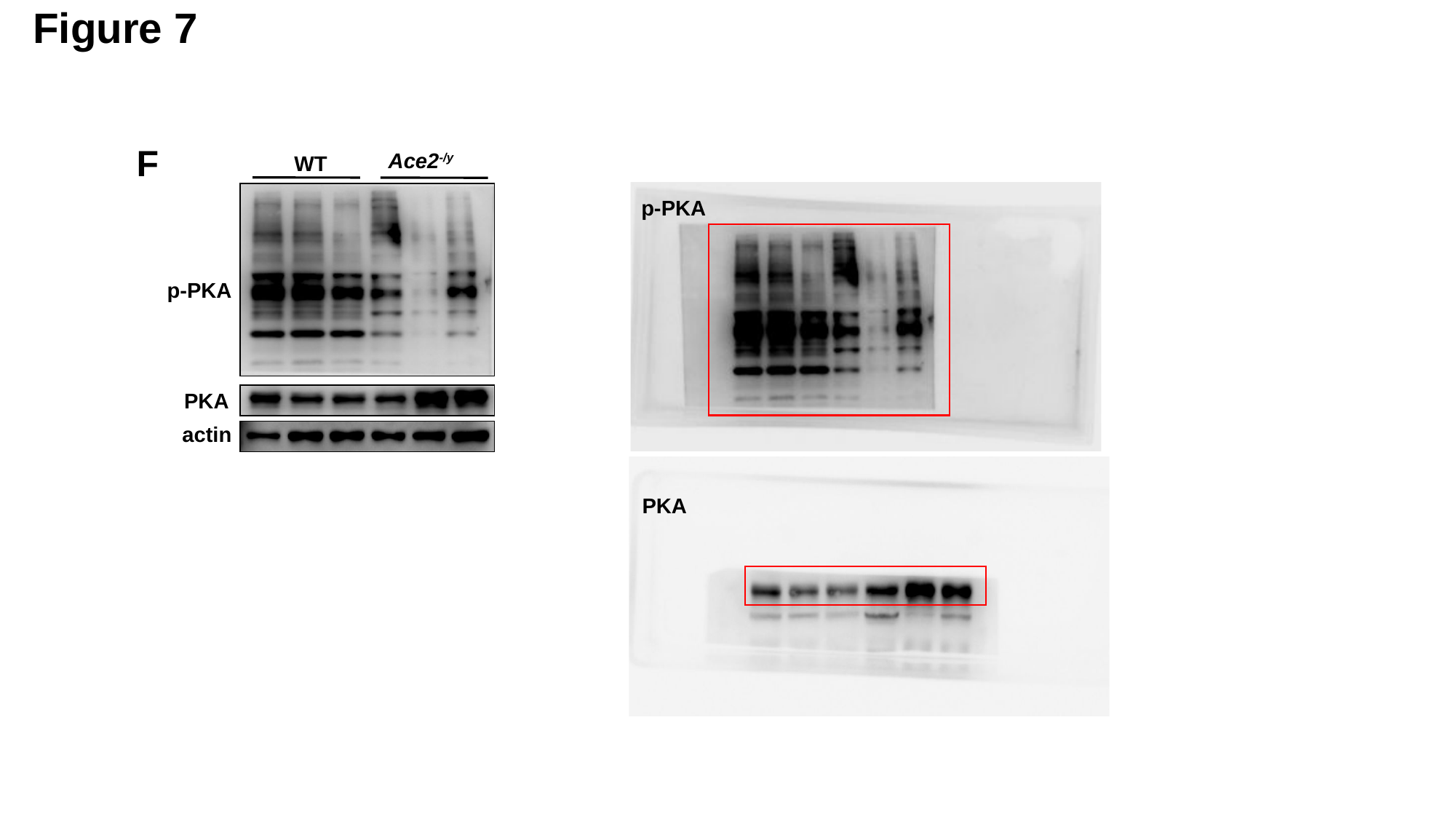

Figure 7
F
WT
Ace2-/y
p-PKA
PKA
p-PKA
PKA
actin

## Slide 4
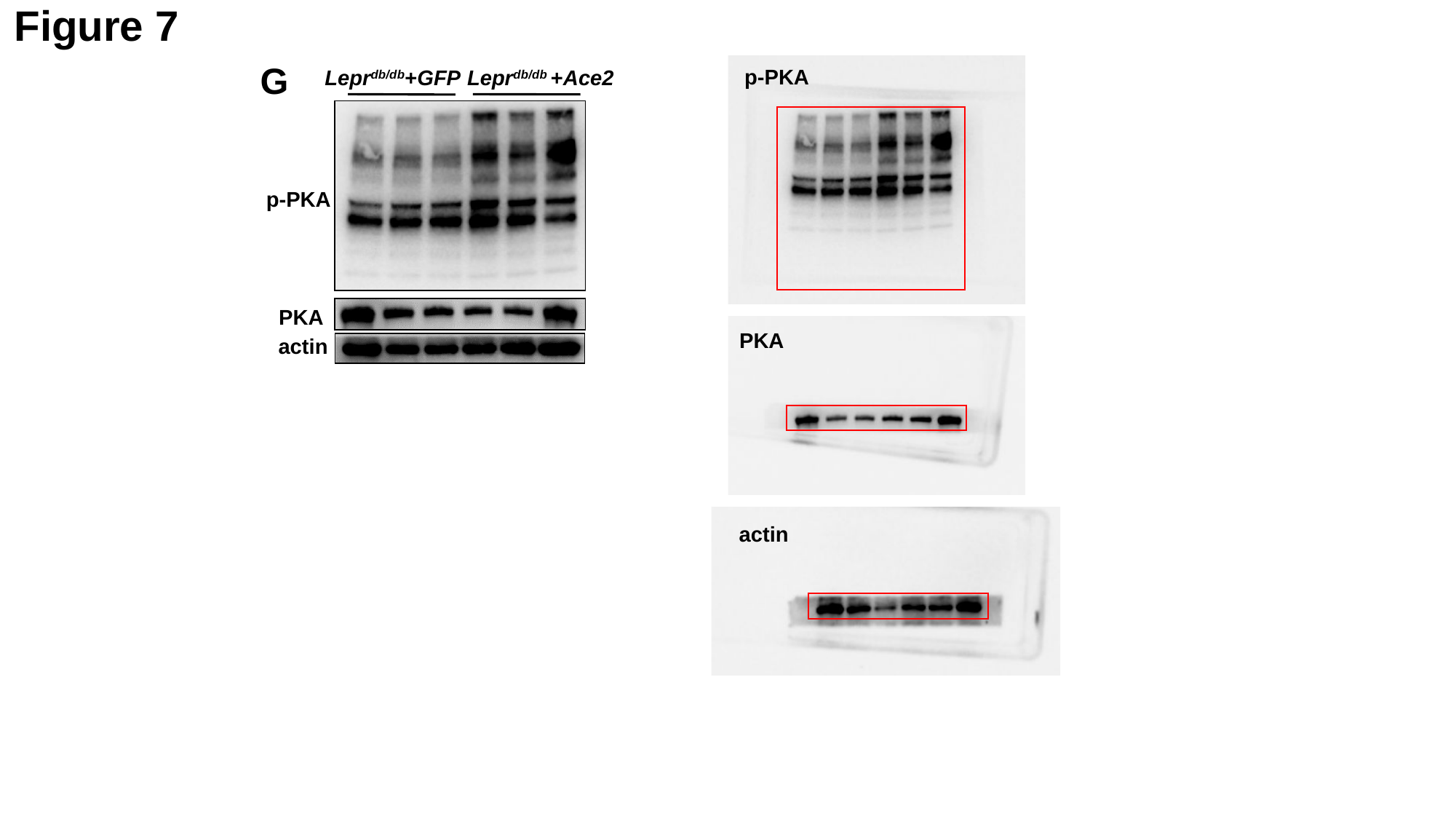

Figure 7
G
p-PKA
Leprdb/db+GFP
Leprdb/db +Ace2
p-PKA
PKA
PKA
actin
actin

## Slide 5
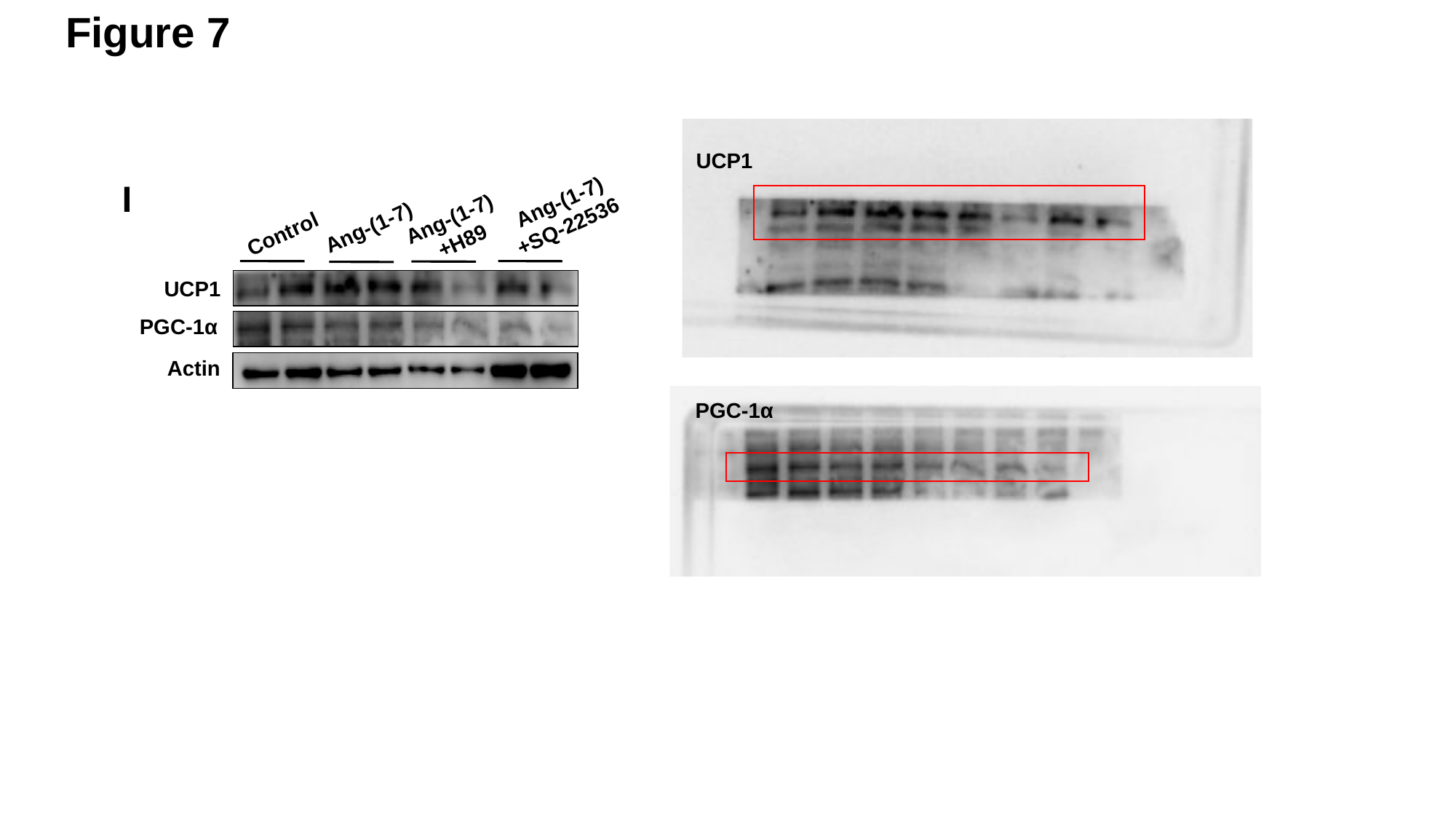

Figure 7
UCP1
I
Ang-(1-7)
+SQ-22536
Ang-(1-7)
 +H89
Ang-(1-7)
Control
UCP1
PGC-1α
Actin
PGC-1α
