# Supplementary material for: ACE2 pathway regulates thermogenesis and energy metabolism
Source: eLife. 2022 Jan 11;11:e72266. doi: 10.7554/eLife.72266 (PMC8776250; doi:10.7554/eLife.72266)
Supplement: Source data 2. [file elife-72266-data2.zip › Source data 2--PowerPoint of gels or blots/Figure 7-figure supplement 2-Ace2 regulate thermogenesis through Akt and PKA -source data 2.pptx]

## Slide 1
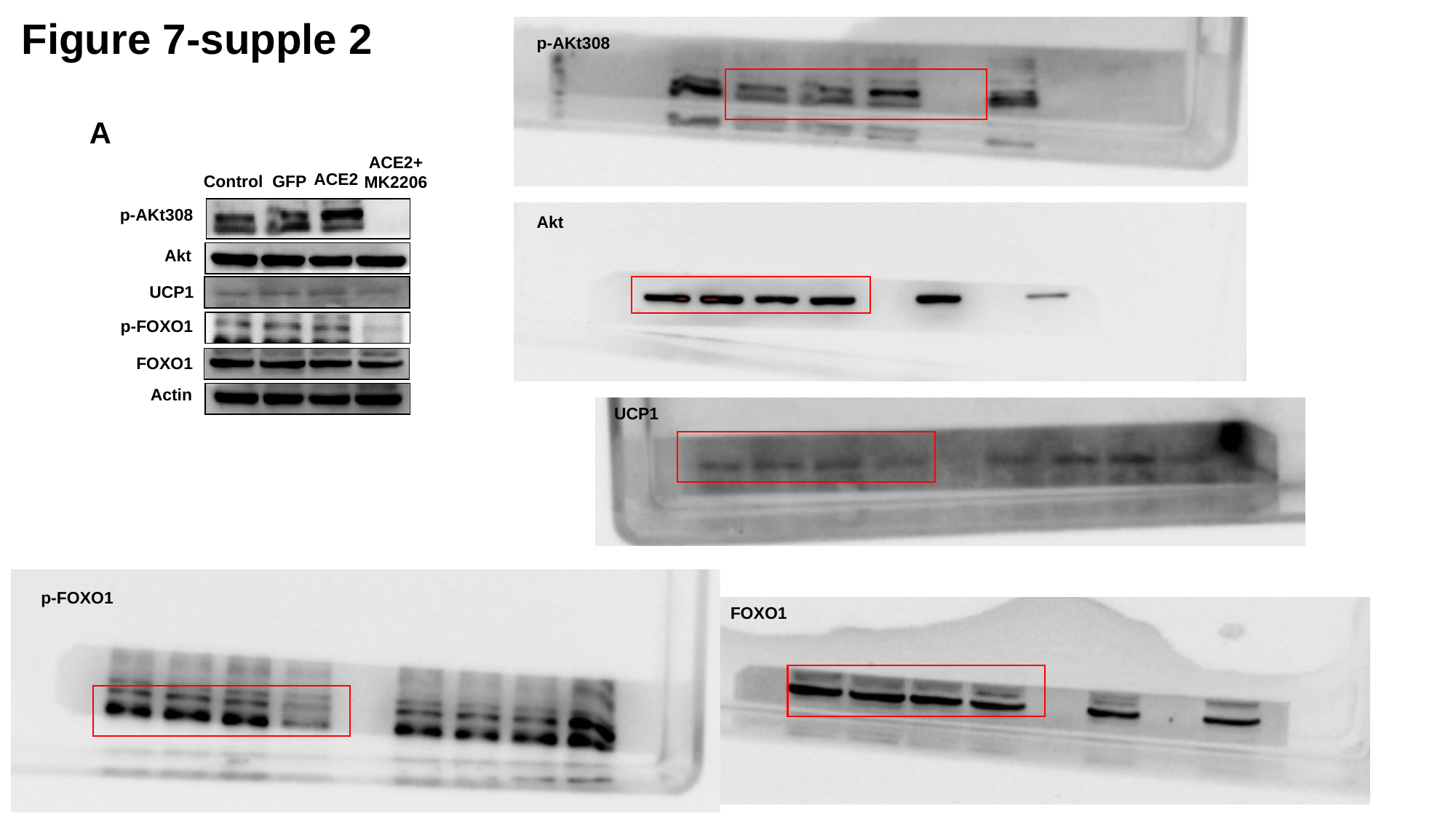

Figure 7-supple 2
p-AKt308
A
ACE2+
MK2206
ACE2
Control
GFP
p-AKt308
Akt
UCP1
p-FOXO1
FOXO1
Actin
Akt
UCP1
p-FOXO1
FOXO1

## Slide 2
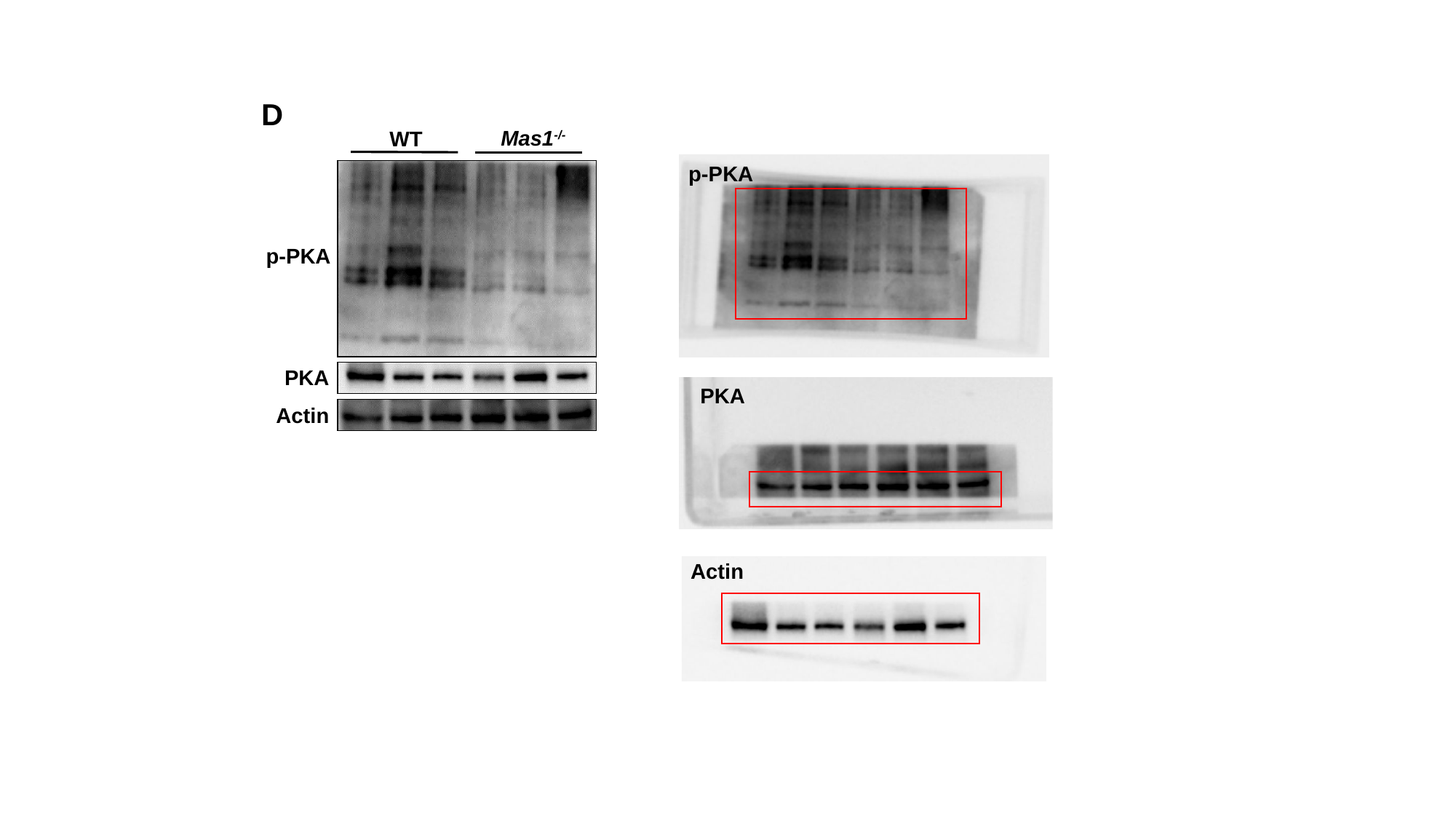

D
Mas1-/-
WT
p-PKA
p-PKA
PKA
PKA
Actin
Actin

## Slide 3
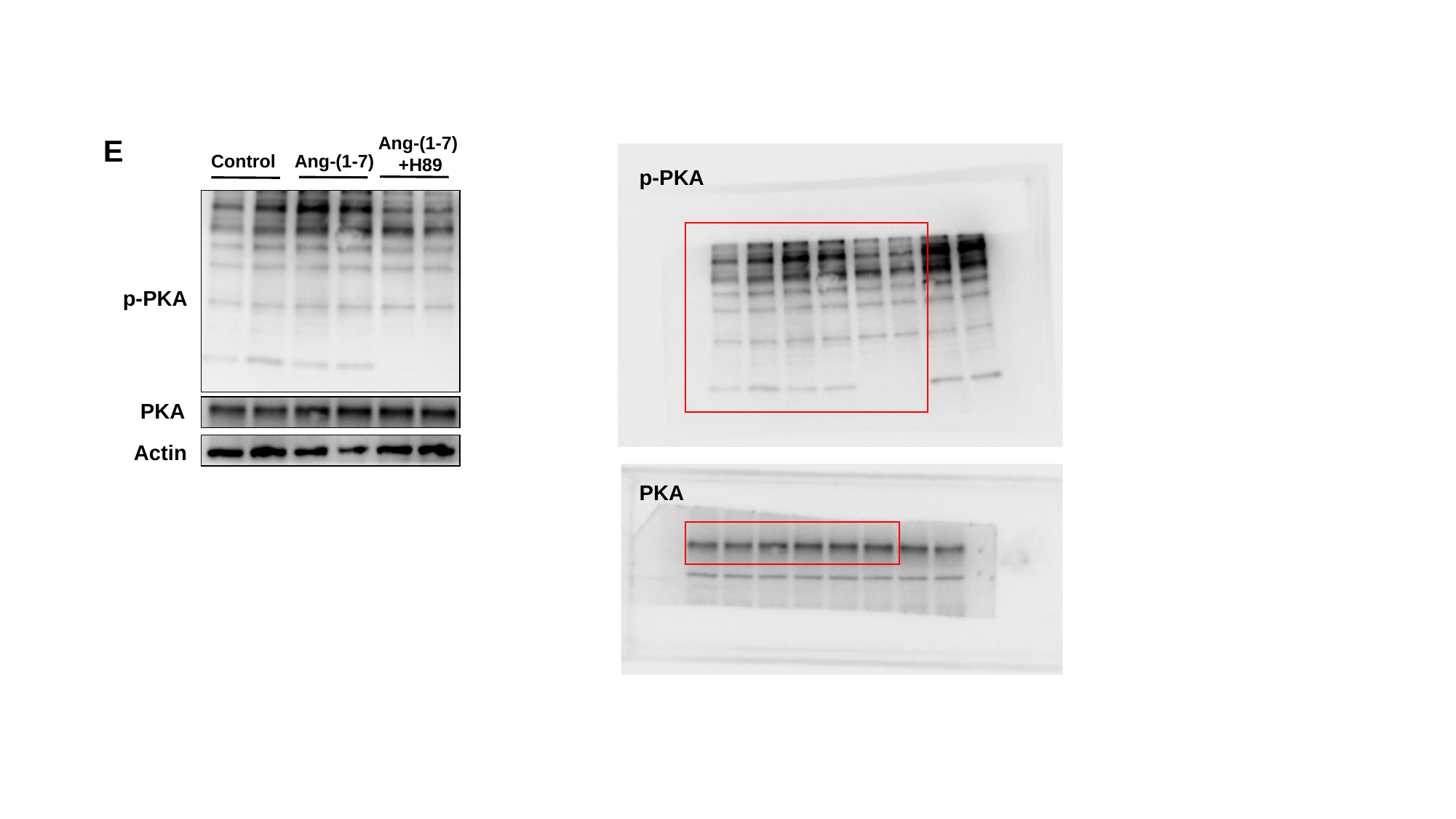

E
Ang-(1-7)
 +H89
Ang-(1-7)
p-PKA
PKA
Control
p-PKA
PKA
Actin
